# Supplementary material for: Defective vascular smooth muscle cell tafazzin impairs mitochondrial function and promotes atherosclerosis in preclinical models
Source: Nat Commun. 2025 Dec 4;16:10909. doi: 10.1038/s41467-025-65873-y (PMC12678788; doi:10.1038/s41467-025-65873-y)
Supplement: Supplementary file 1 — Supplementary Information [file 41467_2025_65873_MOESM1_ESM.pdf]

Supplementary Material

Supplementary Table 1 Primer Sequences

Primer name	Sequence 5’-3’
**Human**	
TAFAZZIN FWD	TGGCATGTCGGAATGAATGAC
TAFAZZIN REV	GCTTCCCGATCAGCACAGT
Beta 2 microglobulin FWD	GAGGCTATCCAGCGTACTCCA
Beta 2 microglobulin REV	CGGCAGGCATACTCATCTTTT
**Mouse**	
Tafazzin FWD	ATGGGCCTAGTTGGCACCTA
Tafazzin REV	AGGGCCTCGGTTCTCAATGA
Il6 FWD	CTCTGGGAAATCGTGGAAAT
Il6 REV	CCAGTTTGGTAGCATCCATC
Mcp1 FWD	AGCTGTAGTTTTTGTCACCAAGC
Mcp1 REV	GTGCTGAAGACCTTAGGGCA
Tnfa FWD	GGTCCCCAAAGGGATGAG
Tnfa REV	CACTTGGTGGTTTGCTACGAC
Il1b FWD	ACAAAATACCTGTGGCCTTGG
Il1b REV	CTTGGGATCCACACTCTCCAG
Ndufs1 FWD	GTGGATGCTGAAGCCTTAGTAGC
Ndufs1 REV	GGAACGTAAGTCTGTACCAGCTC
Ndufb8 FWD	TGTTGCCGGGGTCATATCCTA
Ndufb8 REV	AGCATCGGGTAGTCGCCATA
Nd1 FWD	TCCGAGCATCTTATCCACGC
Nd1 REV	GTATGGTGGTACTCCCGCTG
Nd2 FWD	ATCCTCCTGGCCATCGTACT
Nd2 REV	ATCAGAAGTGGAATGGGGCG
Ndufs8 FWD	GGACAAAGCCTTCATAGCAGCG
Ndufs8 REV	CTGTCCACATCAGAATCCGAGC
Beta 2 microglobulin FWD	TTCTGGTGCTTGTCTCACTGA
Beta 2 microglobulin REV	CAGTATGTTCGGCTTCCCATTC

Supplementary Figures

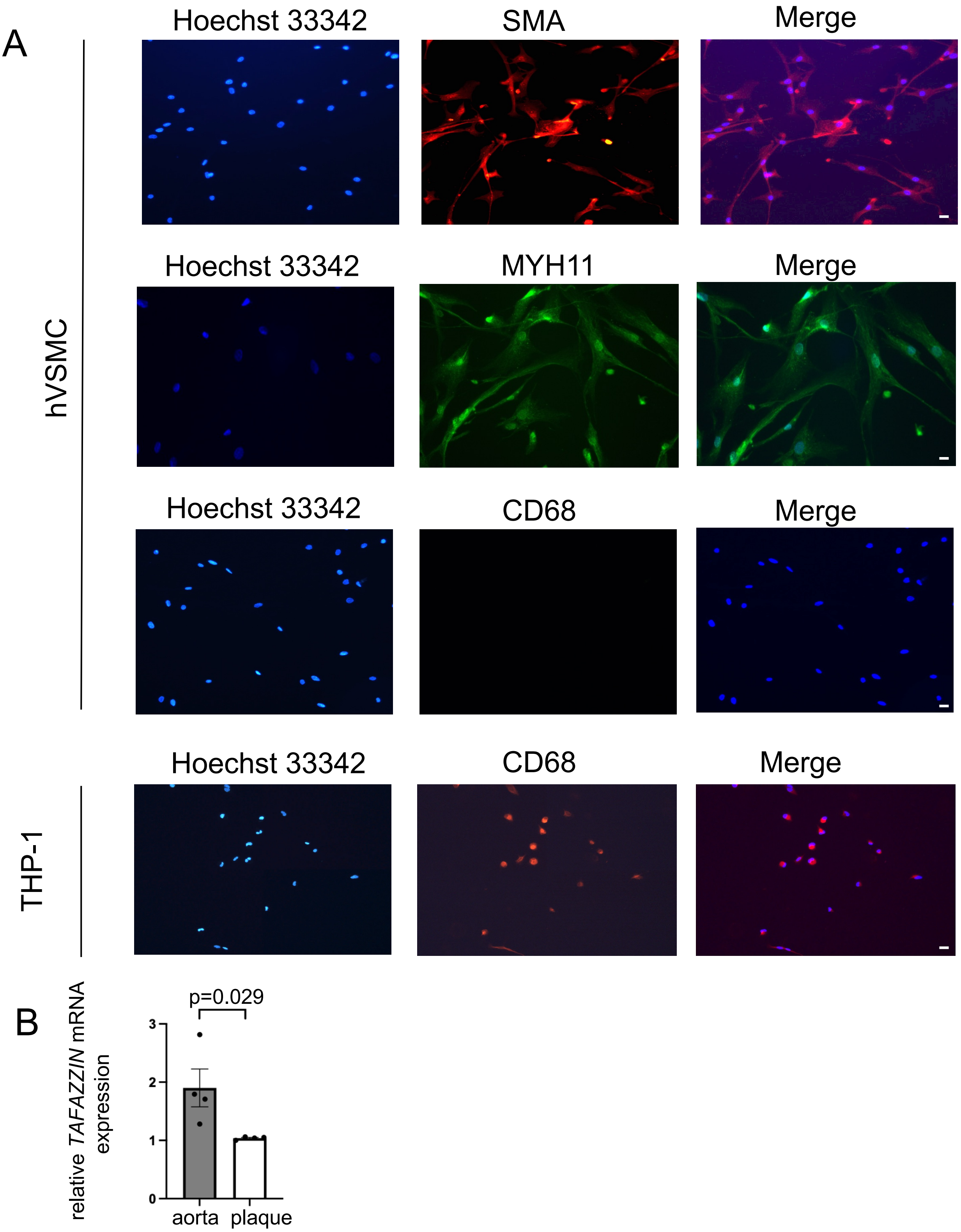

Supplementary Figure 1. Immunocytochemistry and tafazzin expression in VSMCs
A. Isolated human vascular smooth muscle cells (hVSMCs) were cultured in Smooth Muscle Cell Growth Medium, supplemented with 5% Fetal Bovine Serum, and stained for alpha-smooth muscle actin (SMA), smooth muscle myosin heavy chain (MYH11), CD68; Hoechst 33342 used as nuclear stain. The image shown is of hVSMCs cultured from plaque. THP-1 cells were used as a positive control for CD68 staining. Scale bars represent 20 μM. **B** QPCR for *TAFAZZIN* mRNA in human plaque VSMCs or aortic VSMCs. Expression is shown relative to plaque VSMCs, normalized to *B2M* housekeeping gene (n=4, 3 male, 1 female hVSMC donors, two-sided Mann Whitney test). Data are shown as mean ± SEM. Source data are provided as a Source Data file.

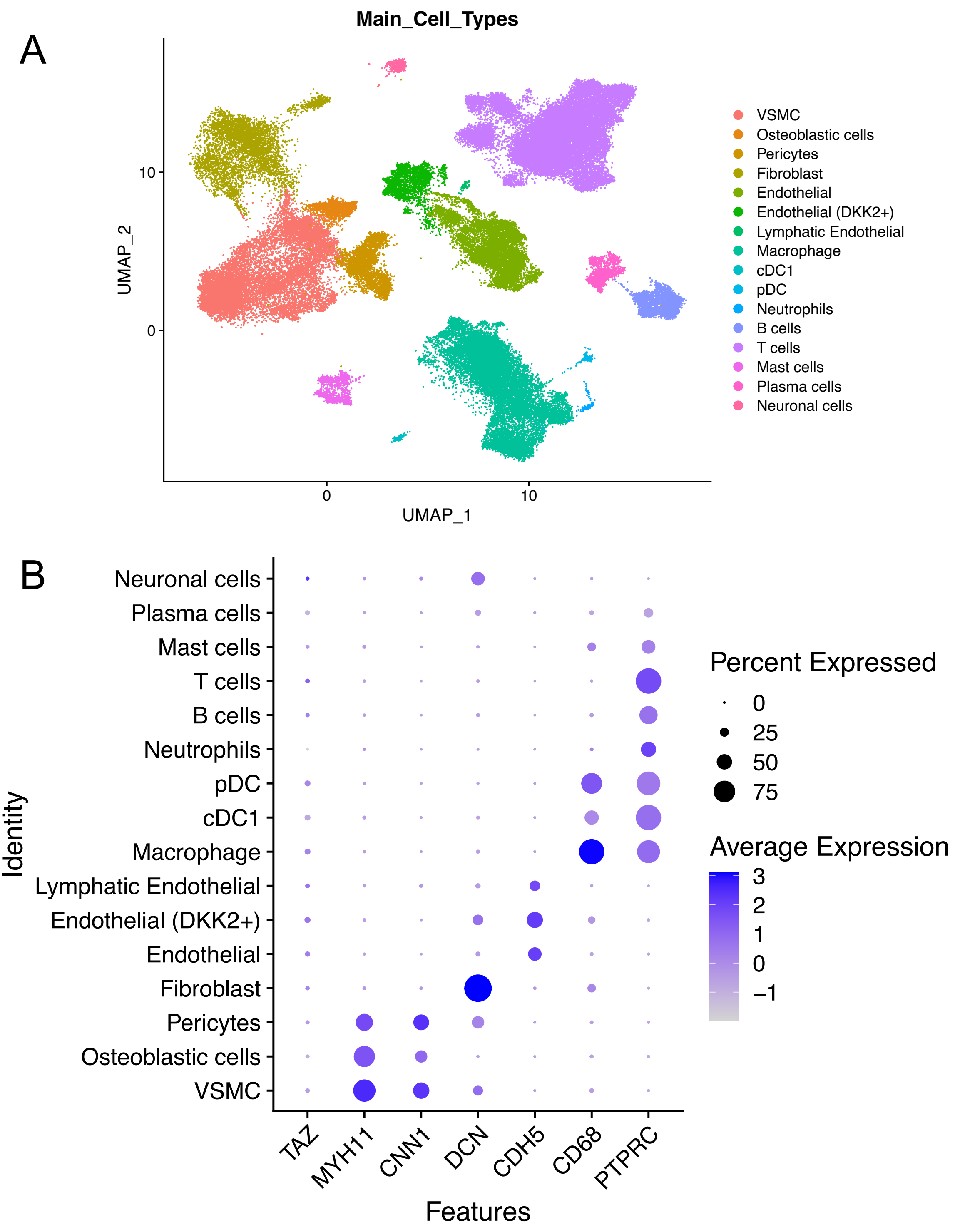

C

Differential TAZ expression	Log2 fold change	p value
VSMCs vs macrophages	-0.598	$1.15x10^{-45}$
VSMCs vs endothelial cells	-0.769	$1.17x10^{-20}$

Supplementary Figure 2. Tafazzin expression is low in plaque VSMCs
Analysis of combined single-cell RNA-seq profiles of carotid (GSE155512, GSE159677) and coronary lesions (GSE131778)(as described in Bleckwehl et al[14]). **A** UMAP (Uniform manifold approximation and projection) of plaque cells showing cell type annotation. **B** Dot plot showing scaled expression levels for *TAFAZZIN* (TAZ) and markers of VSMCs (*MYH11, CNN1*), fibroblasts (*DCN*), endothelial cells (*CDH5*), macrophages (*CD68*) and leukocytes (*PTPRC*). Expression level is shown using a grey-blue color scale. Dot size show the percentage of expressing cells for each cluster. **C** Statistical analysis of TAZ expression in VSMCs compared with macrophages and endothelial cells (Wilcoxon Rank Sum test).

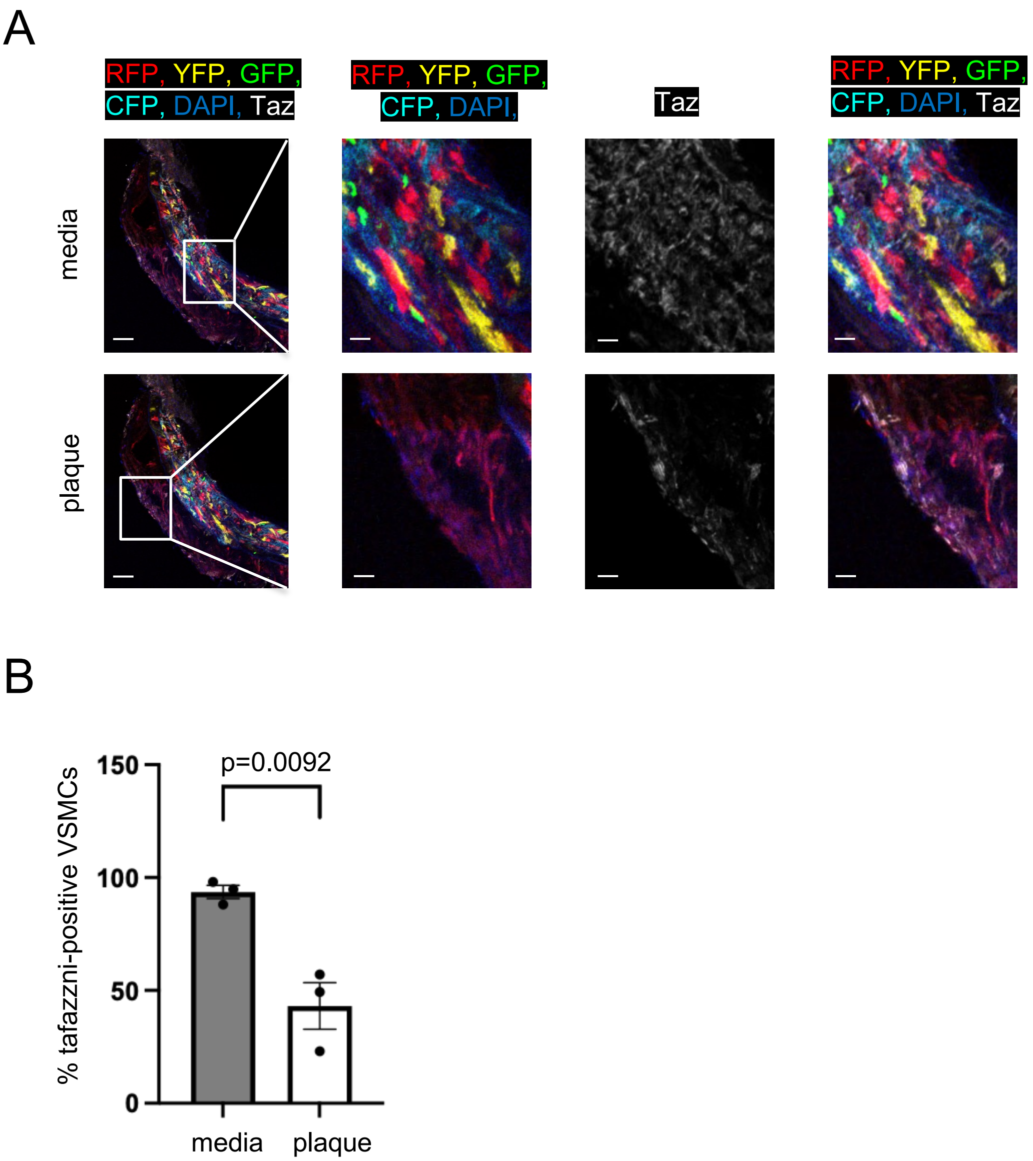

Supplementary Figure 3. Tafazzin expression is decreased in plaque VSMCs
A Confocal images ("max projection" from z-stack) of immunofluorescence staining of plaque and media for DAPI (blue), and tafazzin (Taz)(white) in aortas from Myh11-CreERt2/Rosa26-Confetti/Apoe$^{-/-}$ mice fed high fat diet for 14-23 weeks; the Confetti reporter (red (RFP), yellow (YFP), green (GFP) or cyan (CFP) fluorescent protein) marks VSMCs. Scale bars = 50 μm in low power views, 20 μm in magnified views of outlined areas. **B** Quantification of % Confetti positive VSMCs expressing tafazzin in the media or plaque of atherosclerotic arteries (n=3 male mice per group, two-sided unpaired t test). Data are shown as mean ± SEM. Source data are provided as a Source Data file.

A

Position 421-428 of Taz 3’ UTR wild type 5’ ..GCUGCUCACUACCUCCUCAGGGA..

| | | | | | |

hsa-mir-125a-5p 3’ AGUGUCCAAUUUCCCAGAGUCCCU

B

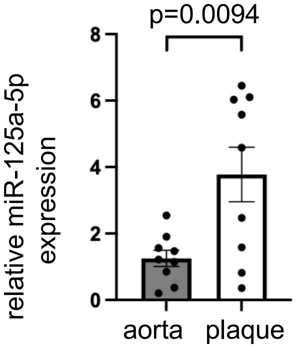

Supplementary Figure 4. MicroRNA 125a-5p (miR-125a-5p) is increased in plaque tissues
A Putative binding of miR-125a-5p within *TAFAZZIN* (Taz) 3’ UTR indicated by TargetScan database[20]. **B** Quantitative PCR for miR-125a-5p in human plaques and aortic tissues. Expression is shown relative to aorta, normalized to U6 small nuclear RNA (*RNU6-1)* (n=9 samples per group, 6 male (M), 3 female (F) donors, two-sided unpaired t test). Data are shown as mean ± SEM. Source data are provided as a Source Data file.

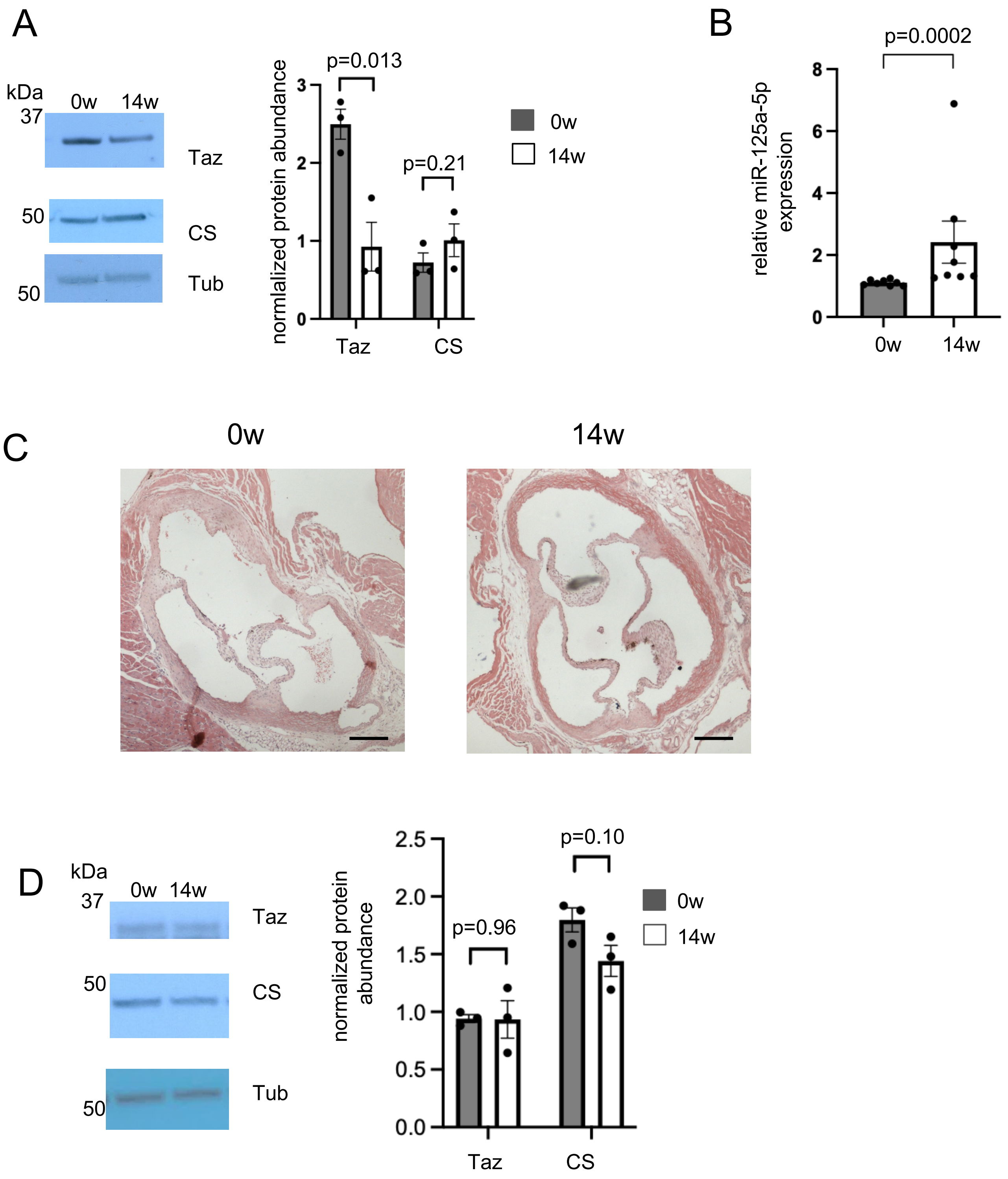

Supplementary Figure 5. Effects of high fat feeding in *Apoe*$^{-/-}$ and *Apoe*$^{+/+}$ mice.
A Representative western blot of tafazzin (Taz) and citrate synthase (CS) in aortas from 6w old Apolipoprotein E-deficient mice (*Apoe*$^{-/-}$) fed a high fat diet for 0 weeks (0w) or 14 weeks (14w) with quantification (n=3, 2 male (M), 1 female (F), two-sided unpaired t test). Tub = tubulin. **B** Quantitative PCR for miR-125a-5p in Apoe$^{-/-}$ mice fed a high fat diet for 0 or 14 weeks. Expression is shown relative to 0 week high fat diet group, normalized to *Rnu6* (n=8, 3M, 5F, two-sided Mann Whitney test). **C** Aortic root sections stained with haematoxylin and eosin from C57BL/6 Apolipoprotein E wild-type mice (*Apoe*$^{+/+}$) mice fed high fat diet for 0 or 14 weeks. Scale bars represent 500 μm. **D** Representative western blot of tafazzin (Taz) and citrate synthase (CS) in aortas from *Apoe*$^{+/+}$ mice fed high fat diet for 0 or 14 weeks with quantification (n=3, 2M, 1F, two-sided unpaired t test). For **A,B,D** n= number of mice per group. For **A** and **D**: Data normalized to citrate synthase for tafazzin or tubulin for citrate synthase. Data are shown as mean ± SEM. Source data are provided as a Source Data file.

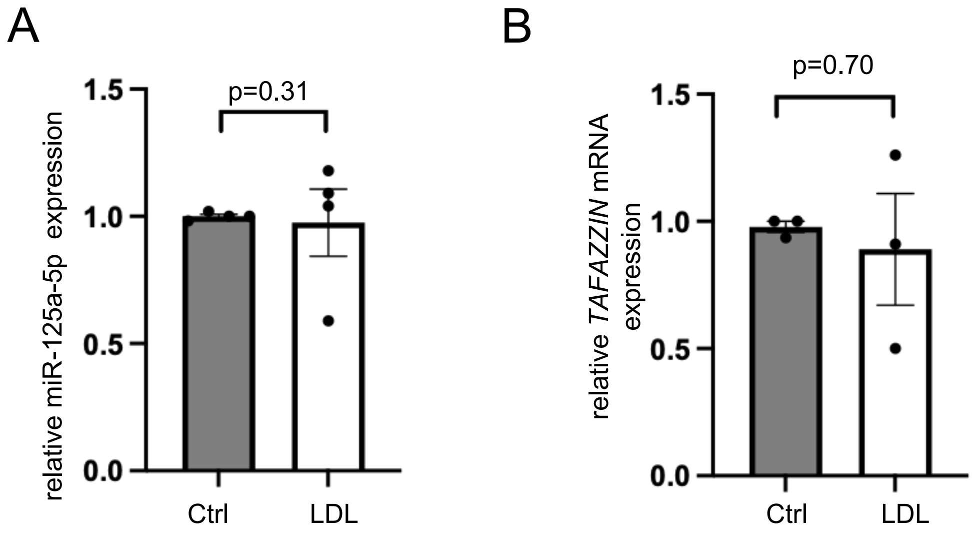

Supplementary Figure 6. Effect of LDL treatment on microRNA 125-5p and tafazzin expression
Quantitative PCR for microRNA 125a-5p (miR-125a-5p) (**A**) and *TAFAZZIN* mRNA (**B**) in control hVSMCs (ctrl) or hVSMCs treated with native low density lipoprotein (LDL). Expression is shown relative to control, normalized to *RNU6-1* (**A**) or *B2M* housekeeping gene (**B**). Data are shown as mean ± SEM from 4 (**A**) and 3 (**B**) independent experiments. VSMCs from 2 male (M), 2 female (F) donors for **A**, 1M, 2F, for **B, t**wo-sided Mann Whitney test. Source data are provided as a Source Data file.

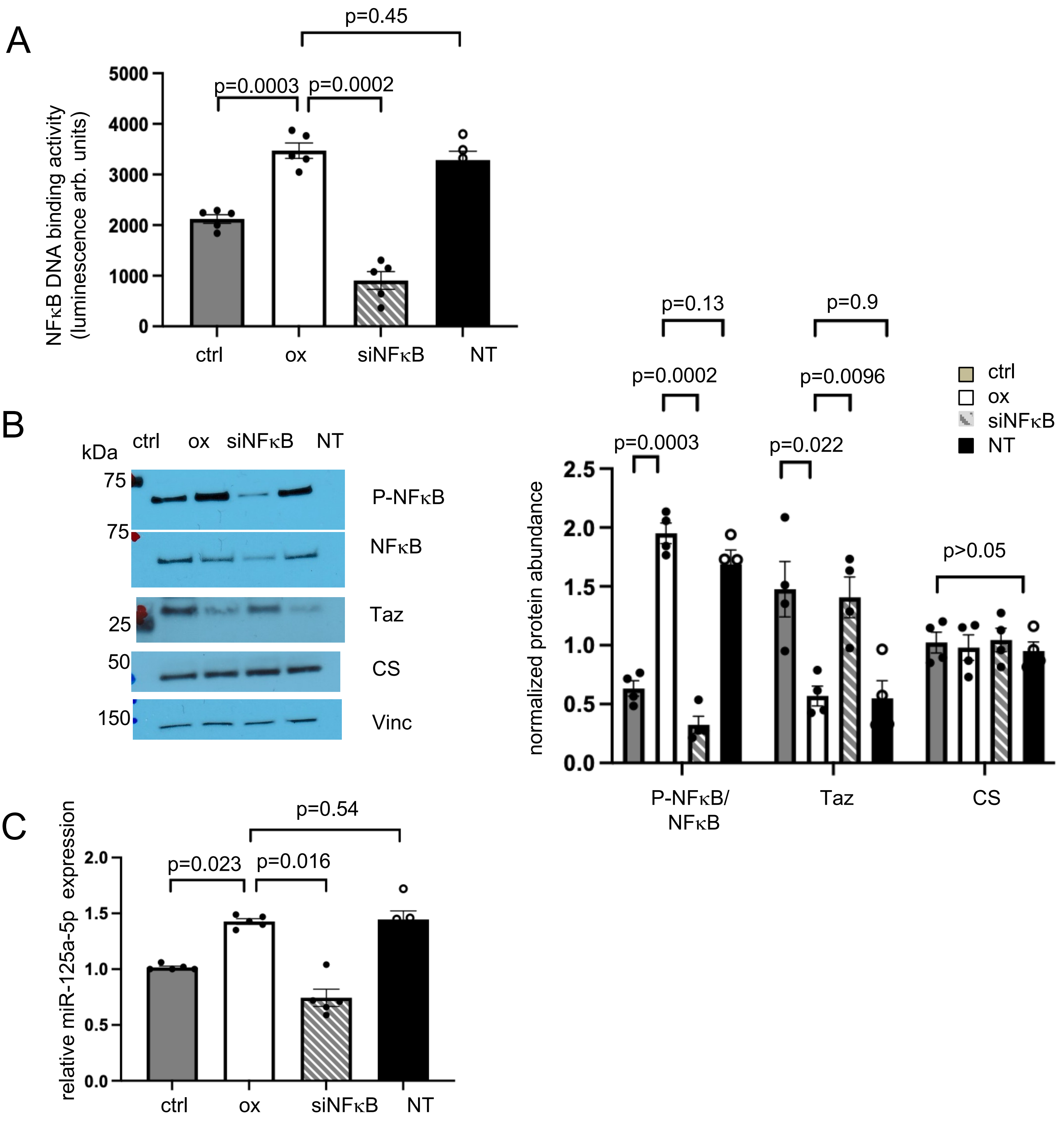

Supplementary Figure 7. Oxidized LDL upregulates microRNA 125a-5p (miR-125a-5p) in a NFκB dependent manner.

A NFκB DNA binding activity determined by ELISA using TransAM NFκB p65 Transcription Factor Assay in control hVSMCs (ctrl) or hVSMCs treated with oxidized LDL (ox), oxLDL+ siRNA against NFκB (si NFκB) or oxLDL+ non-targeting control siRNA (NT)(n=5, 3 male (M), 2 female (F) VSMC donors, 1-way ANOVA, post hoc Bonferroni-Holm). arb. units = arbitrary units. **B** Representative western blot for phosphorylated NFκB (P- NFκB) p65, total NFκB p65 (NFκB), tafazzin (Taz), citrate synthase (CS) in control hVSMCs (ctrl) or hVSMCs treated with oxidized LDL (ox), oxLDL+ siRNA against NFκB (siNFκB) or oxLDL+ non-targeting control siRNA (NT) with quantification. Vinc=vinculin. Data normalized to citrate synthase for tafazzin or vinculin for citrate synthase (n=4, 2M, 2F, 1-way ANOVA, post hoc Bonferroni-Holm). **C** Quantitative PCR for miR-125a-5p in control hVSMCs (ctrl) or hVSMCs treated with oxidized LDL (ox), oxLDL+ siRNA against NFκB (siNFκB) or oxLDL+ non-targeting control siRNA (NT). Expression is shown relative to control, normalized to *RNU6-1* (n=5, 3M, 2F, Kruskal-Wallis test, post-hoc Bonferroni-Holm). Data are shown as mean ± SEM with multiplicity adjusted p values. Source data are provided as a Source Data file.

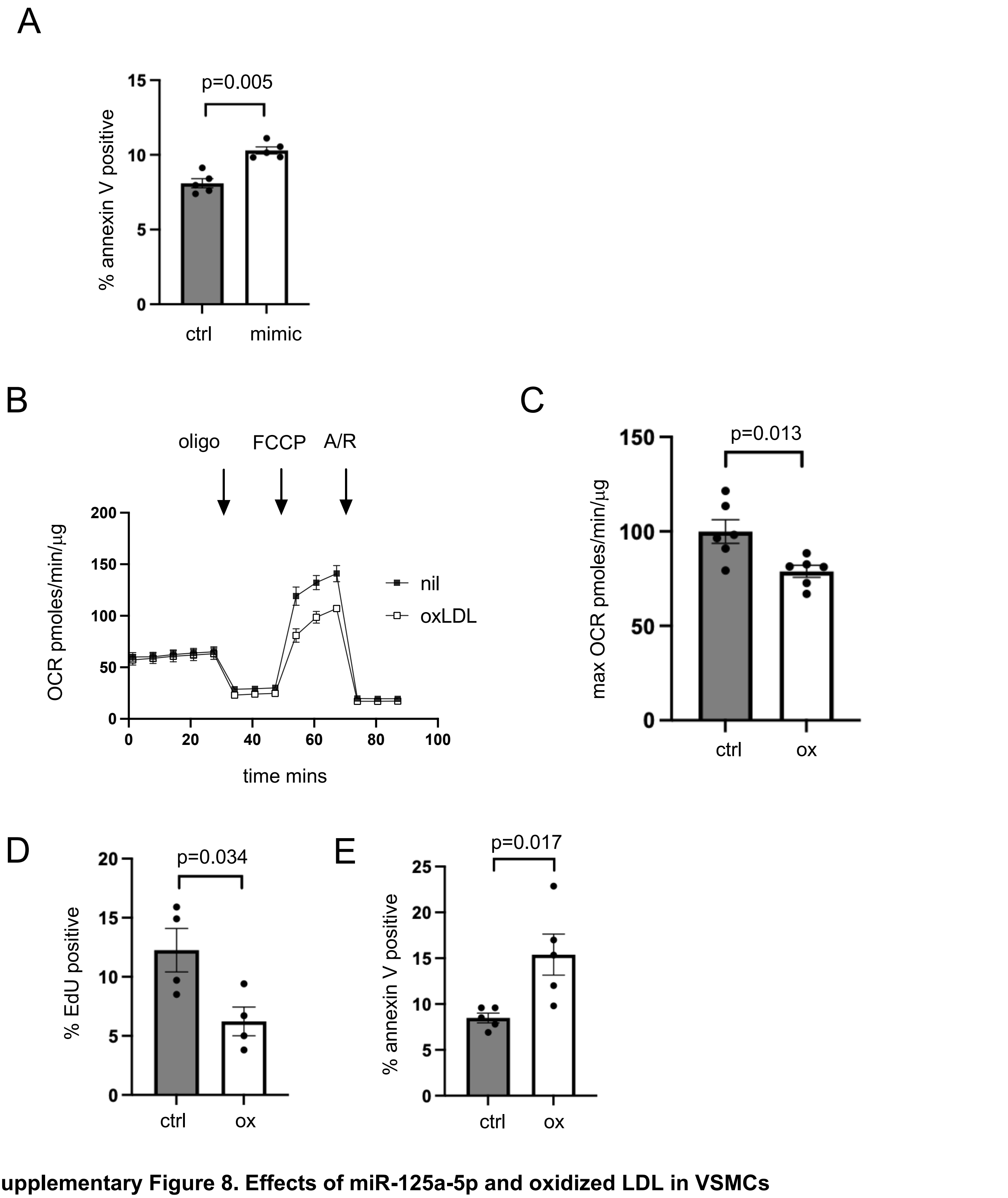

Supplementary Figure 8. Effects of miR-125a-5p and oxidized LDL in VSMCs
A Percentage of cells positive for annexin V in hVSMCs transfected with non-targeting control (ctrl) or miR-125a-5p mimic (n=5, 3 male (M), 2 female (F) VSMC donors, two-sided unpaired t test). **B** Representative Seahorse profiles of oxygen consumption rate (OCR) with sequential addition of oligomycin (oligo), FCCP and antimycin/rotenone (A/R) in control hVSMCs (ctrl) or hVSMCs treated with oxidized low density lipoprotein (ox)(n=3, 2M, 1F). **C-E: C** Maximal OCR after FCCP (n=6, 3M, 3F, two-sided unpaired t test). **D** percentage of cells positive for EdU (n=4, 2M, 2F, two-sided unpaired t test) and **E** percentage of cells positive for annexin V (n=5, 3M, 2F, two-sided unpaired t test) in control hVSMCs (ctrl) or hVSMCs treated with oxidized low density lipoprotein (ox). For **A-E** n=number of independent experiments. Data are shown as mean ± SEM. Source data are provided as a Source Data file.

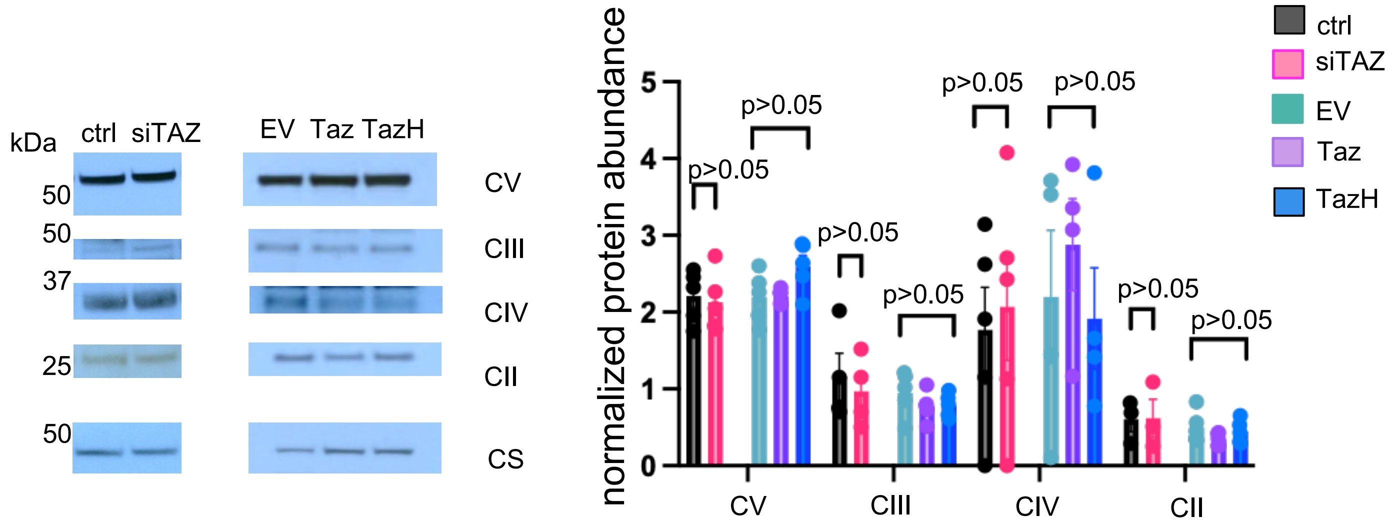

Supplementary Figure 9. Respiratory complex subunit abundance in *TAFAZZIN* silenced VSMCs and VSMCs overexpressing tafazzin or Taz^{H69Q}

Representative western blot for respiratory complexes II to V subunits in hVSMCs transfected with non-targeting control (ctrl) or siRNA against *TAFAZZIN* (siTAZ) and in mVSMCs overexpressing empty vector (EV), tafazzin (Taz) or transacylase mutant tafazzin (Taz^{H69Q} or TazH) with quantification. CS= citrate synthase. Data normalized to citrate synthase (n=5 independent experiments, 2 male, 3 female VSMC donors; two-sided Mann Whitney for siTAZ vs control comparisons; Kruskal-Wallis for EV, Taz and TazH VSMC comparisons.) Data are shown as mean ± SEM. Source data are provided as a Source Data file.

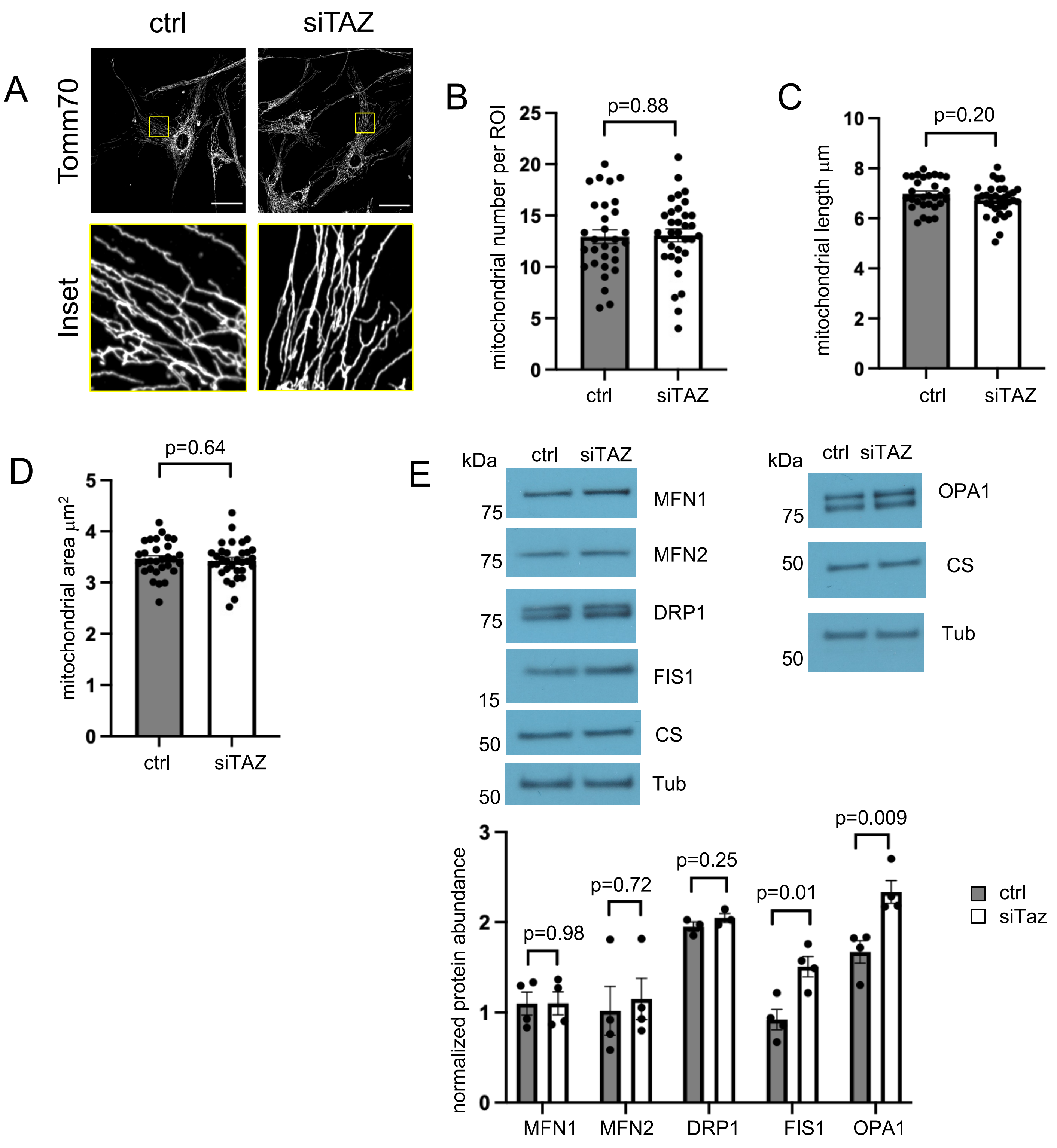

Supplementary Figure 10. Mitochondrial morphology in *TAFAZZIN* silenced VSMCs
A Confocal imaging of mitochondria labelled with Tomm70 (translocase of outer mitochondrial membrane 70) antibody in hVSMCs transfected with control siRNA (ctrl) or siRNA against *TAFAZZIN* (siTAZ). Scale bar =50 μm. Mitochondrial morphology was quantified as mitochondrial number per region of interest (ROI) (**B**), mitochondrial length (**C**) and mitochondrial area (**D**) in ctrl or siTAZ hVSMCs. (**B,D**: two-sided unpaired t test; **C**: two-sided Mann Whitney test; data are shown as mean ± SEM from 3 independent experiments. VSMCs were female-derived). **E** Representative western blots for MFN1, MFN2, DRP1, FIS1, OPA1 in ctrl or siTAZ hVSMCs with quantification. CS= citrate synthase, Tub = tubulin. Data normalized to citrate synthase (n=4 independent experiments, 1 male, 3 female VSMC donors, two-sided unpaired t test). Data are shown as mean ± SEM. Source data are provided as a Source Data file.

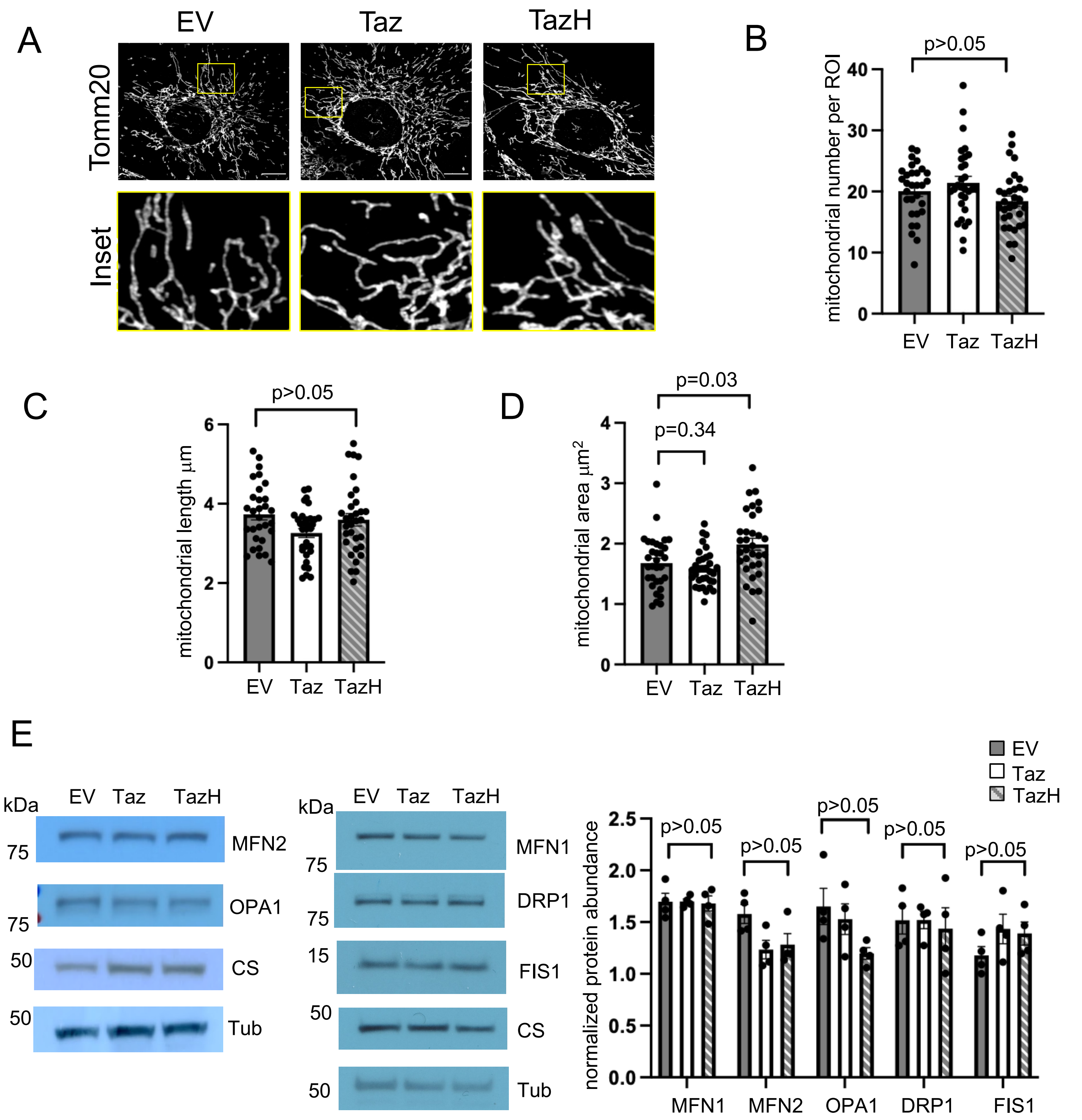

Supplementary Figure 11. Mitochondrial morphology in VSMCs overexpressing tafazzin or Taz^{H69Q}
A Confocal imaging of mitochondria labelled with Tomm20 antibody in mVSMCs overexpressing empty vector (EV), tafazzin (Taz) or transacylase mutant tafazzin (Taz^{H69Q} or TazH). Scale bar = 10 µm. Mitochondrial morphology quantified as mitochondrial number per ROI (**B**), mitochondrial length (**C**) and mitochondrial area (**D**) in EV, Taz and TazH mVSMCs. **B-D**: 1-way ANOVA with Bonferroni-Holm post hoc. Data are shown as mean ± SEM from 3 independent experiments. VSMCs were female-derived. **E** Representative western blots for MFN1, MFN2, OPA1, DRP1, FIS1 in EV, Taz and TazH mVSMCs with quantification. CS= citrate synthase, Tub = tubulin. Data normalized to citrate synthase (n=4 independent experiments, 1 male, 3 female VSMC donors;1-way ANOVA). Data are shown as mean ± SEM. Source data are provided as a Source Data file.

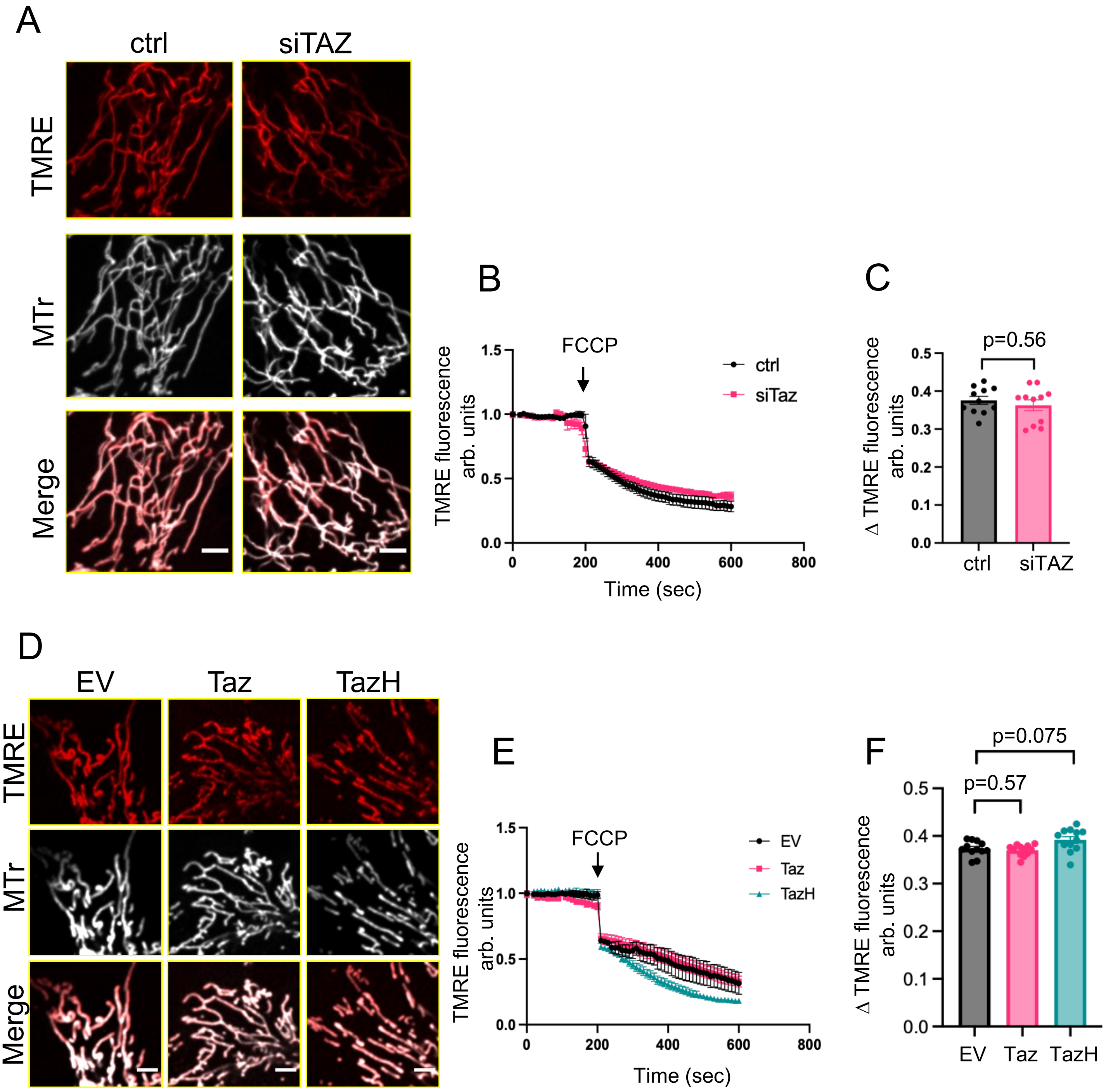

Supplementary Figure 12. Mitochondrial membrane potential in *TAFAZZIN* silenced VSMCs and VSMCs overexpressing tafazzin or Taz^{H69Q}

A Snapshot of live cell confocal imaging of mitochondria stained with tetramethyl rhodamine ethyl ester (TMRE) and Mitotracker Deep Red (MTr) in VSMCs transfected with control siRNA (ctrl) or siRNA against *TAFAZZIN* (siTAZ) at t=0 sec. Scale bar =5 μm. **B** Normalized TMRE fluorescence intensity pre and post application of carbonyl cyanide 4-(trifluoromethoxy)phenylhydrazone (FCCP) and **C** FCCP-induced change in TMRE fluorescence in control and siTAZ VSMCs (two-sided Mann Whitney test). **D** Snapshot of live cell confocal imaging of mitochondria stained with tetramethyl rhodamine ethyl ester (TMRE) and Mitotracker Deep Red (MTr) in VSMCs overexpressing empty vector (EV), tafazzin (Taz) or transacylase mutant tafazzin (Taz^{H69Q} or TazH) at t=0 sec. Scale bar = 5 μm. **E** Normalized TMRE fluorescence pre and post application of FCCP and **F** FCCP-induced change in TMRE fluorescence in EV, Taz and TazH VSMCs (1-way ANOVA with Bonferroni-Holm post hoc). arb. units =arbitrary units. Data are shown as mean ± SEM from 3 independent experiments. VSMCs were from female donors. Source data are provided as a Source Data file.

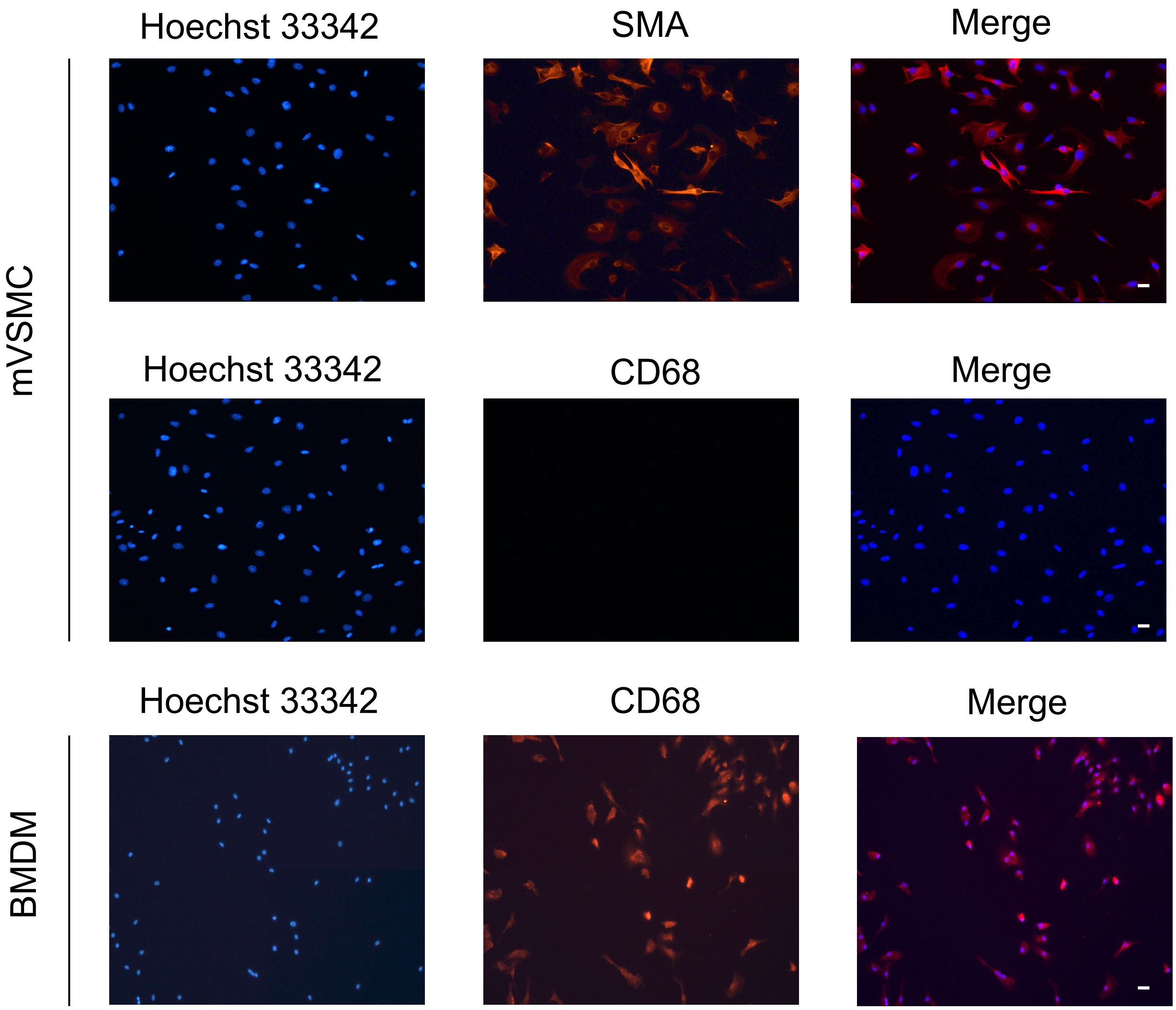

Supplementary Figure 13. VSMCs stained for alpha-smooth muscle actin and CD68
Isolated mouse vascular smooth muscle cells (mVSMCs) were stained for alpha–smooth muscle actin (SMA) and CD68; Hoechst 33342 used as nuclear stain. Bone marrow-derived macrophages (BMDMs) were used as a positive control for CD68 staining. Scale bars represent 15 μM.

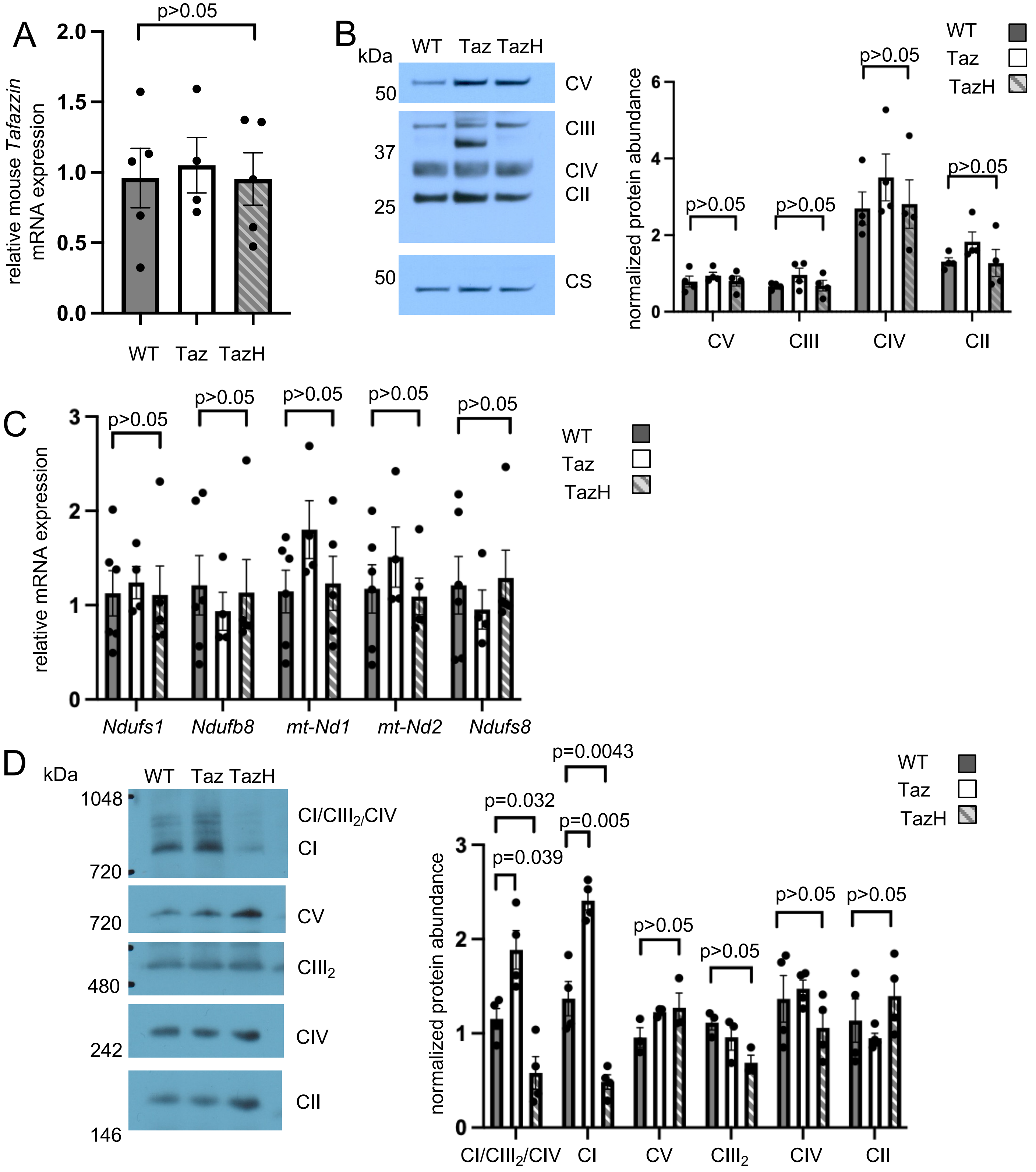

Supplementary Figure 14. Characterization of *Sm22a-Taz* and *Sm22a-Taz^{H69Q}* VSMCs

A QPCR for mouse *Tafazzin* mRNA in VSMCs from wild type (WT), *Sm22a-Taz* (Taz) and *Sm22a-Taz^{H69Q}* (TazH) mice. Expression is shown relative to wild type mice, normalized to *B2m* housekeeping gene (WT, TazH n=5, 2 male (M), 3 female (F) VSMC donors; Taz n=4, 1M, 3F; 1-way ANOVA). **B** Representative western blot with quantification for respiratory complexes II to V subunits in WT, Taz and TazH VSMCs. CS= citrate synthase. Data normalized to citrate synthase (n=4, 2M, 2F, 1-way ANOVA). **C** QPCR for complex I subunits *Ndufs1, Ndufb8, mt-Nd1, mt-Nd2, Ndufs8* mRNA expression in WT, Taz and TazH VSMCs. Expression is shown relative to wild type mice, normalized to *B2m* housekeeping gene (WT n=6, 3M, 3F; Taz n=4, 2M, 2F; TazH n=5, 2M, 3F; Kruskal-Wallis). **D** Blue native polyacrylamide gel electrophoresis and immunoblot analysis of assembled respiratory complexes I to V in WT, Taz and TazH VSMCs permeabilized in1% digitonin. CI/$CIII_2$/CIV =complex I, III, IV supercomplex, $CIII_2$= complex III homodimer. Data normalized to complex IV for complex II, and to complex II for all other complexes (n=4, 2M, 2F, 1-way ANOVA, Bonferroni Holm post hoc). For **A-D** n=number of VSMC isolates. Data are shown as mean ± SEM with multiplicity adjusted p values. Source data are provided as a Source Data file.

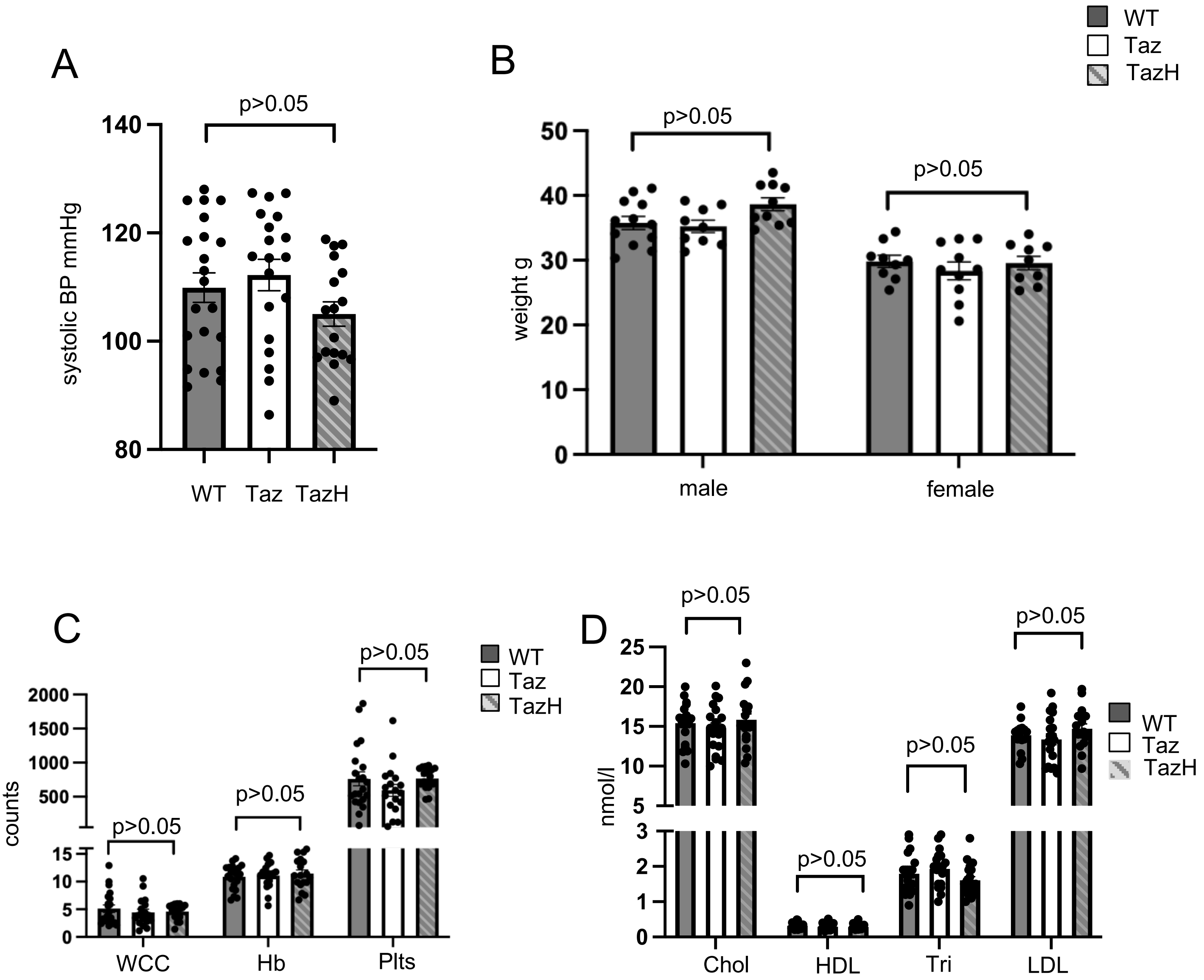

Supplementary Figure 15. *Sm22a-Taz/Apoe$^{-/-}$* and *Sm22a-Taz^{H69Q} /Apoe$^{-/-}$* mice show unchanged blood pressure, body weight, full blood count and lipid profile.
A Systolic blood pressure in wild type *Apoe$^{-/-}$* (WT), *Sm22a-Taz/Apoe$^{-/-}$* (Taz) and *Sm22a-Taz^{H69Q} /Apoe$^{-/-}$* (TazH) mice undergoing high fat diet feeding for 14 weeks (WT n=21, 12 male (M), 9 female (F); Taz n=19, 9M, 10F; TazH n=17, 9M, 8F; 1-way ANOVA.). **B** Body weights in experimental mice (WT n= 21, 12M, 9F; Taz n=19, 9M, 10F; TazH n=19, 10M, 9F; 1-way ANOVA). **C** Full blood count in experimental mice: WCC = white cell count, Hb = haemoglobin, Plts= platelets. Counts are expressed as $10^3/mm^3$ for WCC and platelets, g/dl for haemoglobin (WT n=21, 12M, 9F; Taz n=18, 8M, 10F; TazH n=18, 9M, 9F; Kruskal-Wallis). **D** Lipid profile in wild type *Apoe$^{-/-}$* (WT), *Sm22a-Taz/Apoe$^{-/-}$* (Taz) and *Sm22a-Taz^{H69Q} /Apoe$^{-/-}$* (TazH) after 14 weeks high fat diet (WT n= 21, 12M, 9F; Taz n=19, 9M, 10F; TazH n=19, 10M, 9F; 1-way ANOVA). Chol= cholesterol, Tri =triglycerides. For **A-D** n=number of mice per group. Data shown as mean ± SEM. Source data are provided as a Source Data file.

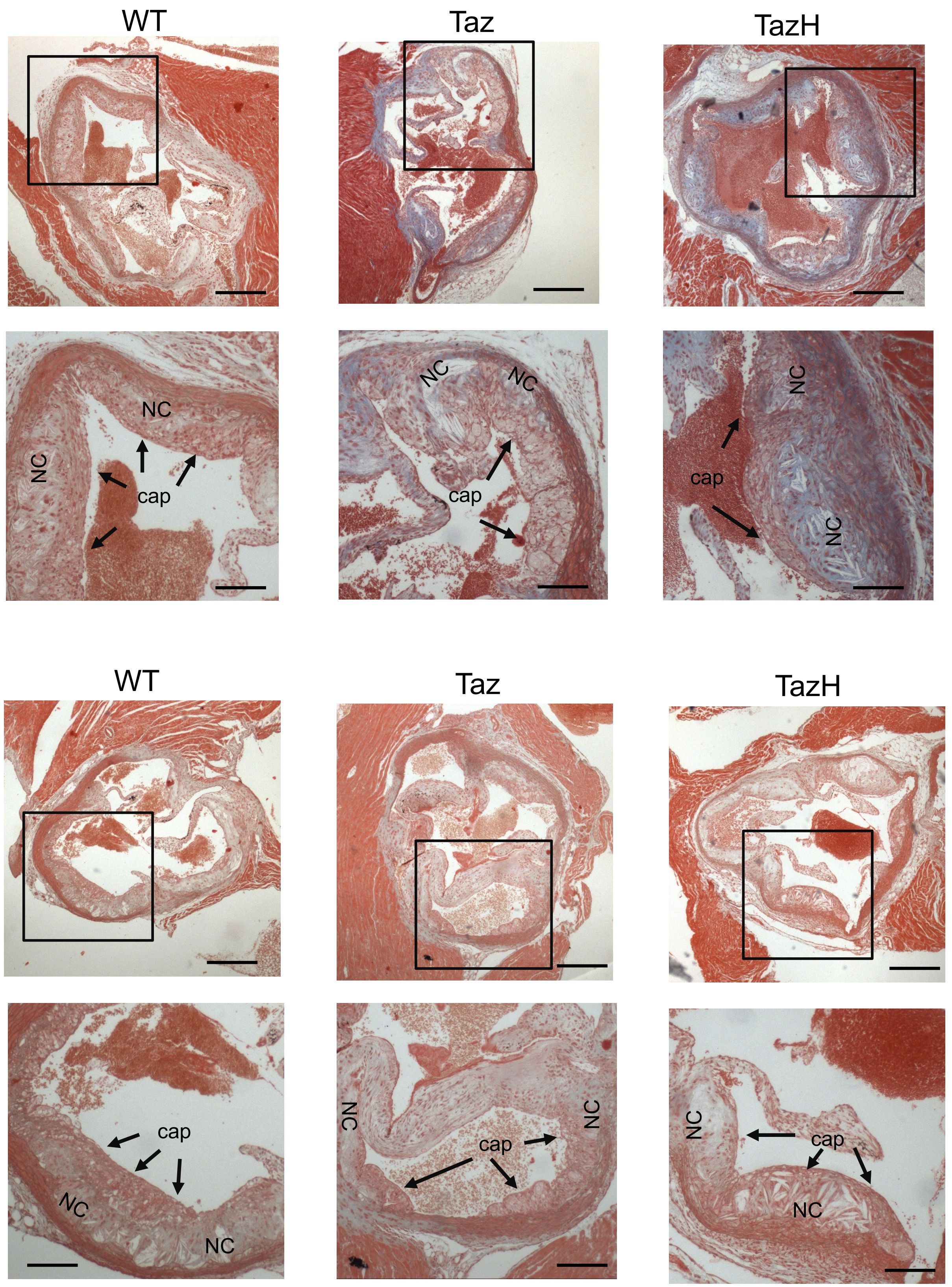

Supplementary Figure 16. Analysis of plaque cap and core in wild type *Apoe$^{-/-}$*, *Sm22a-Taz/Apoe$^{-/-}$* and *Sm22a-Taz^{H69Q}/Apoe$^{-/-}$* mice

Aortic root sections of wild type Apoe$^{-/-}$ (WT), *Sm22a-Taz/Apoe$^{-/-}$*(Taz) and *Sm22a-Taz^{H69Q}/Apoe$^{-/-}$* (TazH) mice after 14 weeks high fat diet stained with Masson's Trichrome. Plaque cap (cap) was defined as the area rich in VSMCs (red) overlying the cholesterol rich, acellular core (NC) as indicated. Scale bars represent 500 μm in low power images, 200 μm in higher power images.

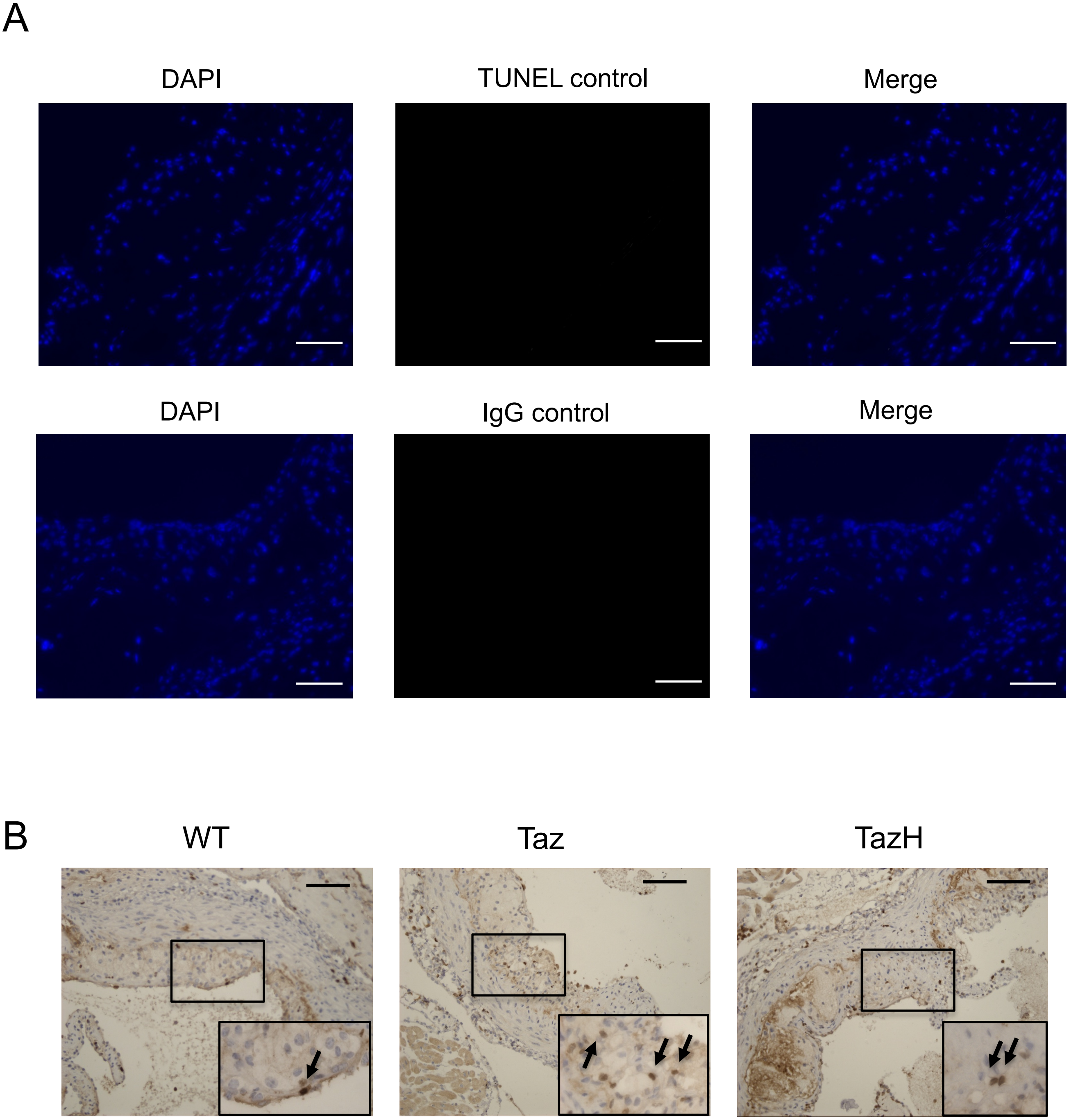

Supplementary Figure 17. Analysis of plaque composition in wild type Apoe$^{-/-}$, *Sm22a-Taz/Apoe$^{-/-}$* and *Sm22a-Taz^{H69Q}/Apoe$^{-/-}$* mice

A. Control aortic root sections of wild type *Apoe$^{-/-}$* mice after 14 weeks high fat diet. For the TUNEL control, TUNEL staining (white channel) was performed with the terminal deoxynucleotidyl transferase reaction omitted. For the immunohistochemistry control (lower panel), mouse IgG control antibody was used followed by incubation with Alexa Fluor 488-labelled secondary (green channel). Nuclei were stained with DAPI (blue). Scale bars represent 100 μm. **B** Aortic root sections of wild type *Apoe$^{-/-}$* (WT), *Sm22a-Taz/Apoe$^{-/-}$*(Taz) and *Sm22a-Taz^{H69Q}/Apoe$^{-/-}$* (TazH) mice after 14 weeks high fat diet stained with Ki67. Scale bars represent 100 μm.

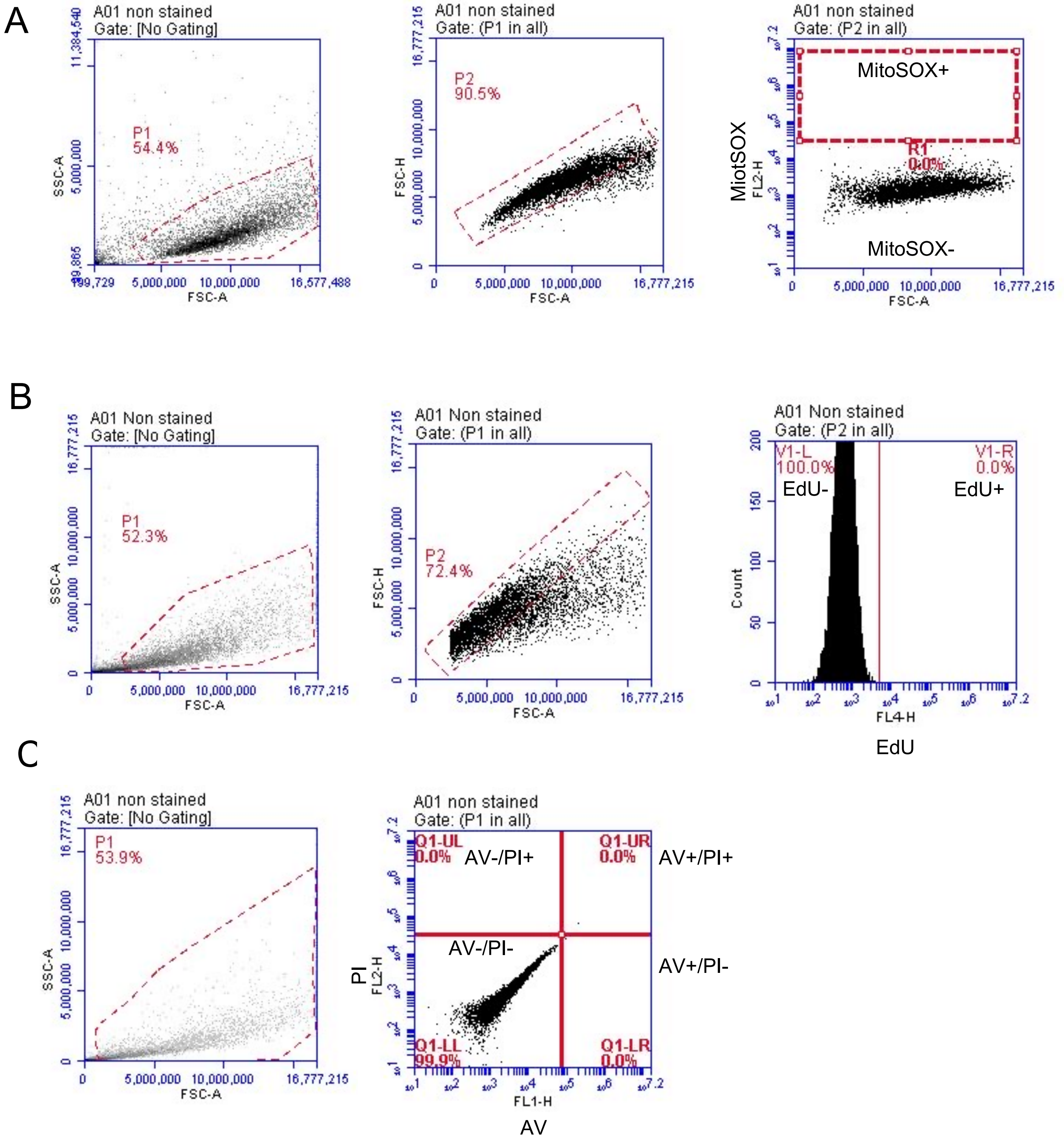

Supplementary Figure 18. Gating strategies for flow cytometric analysis

A. For MitoSOX analysis cells were selected based on FSC/SSC plots, and single cells identified on FSC-A/FSC-H plots. The gate for mitoSOX positive cells was determined based on non stained cells **B.** For EdU analysis cells were selected based on FSC/SSC plots, and single cells identified on FSC-A/FSC-H plots. The gate for EdU positive cells was determined based on non stained cells. **C** For annexin V analysis, FSC-A/FSC-H plots were used to identify all cells but exclude debris. The gate for positive cells was determined based on non stained cells.

Full unedited blots for westerns presented in Supplementary Figures

Sample used in the manuscript

Sample used for quantification

Full unedited blots for Supplementary Figure 5A

n 1

vinculin

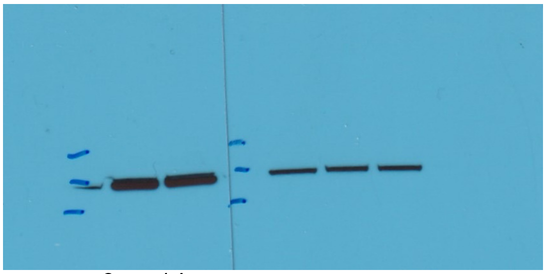

Cut between 75kDa and 100 kDa

tafazzin

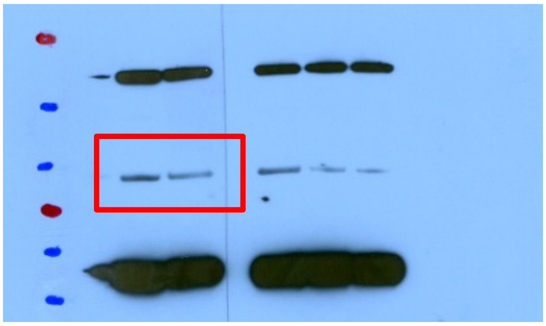

tubulin

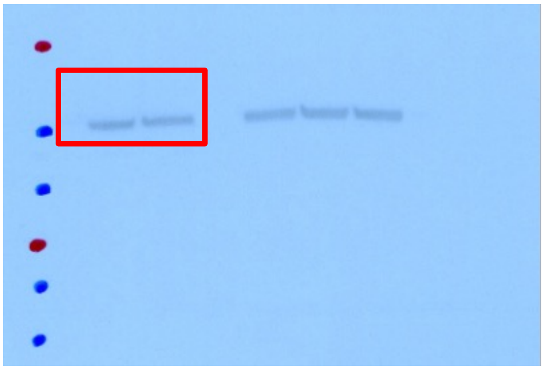

citrate synthase

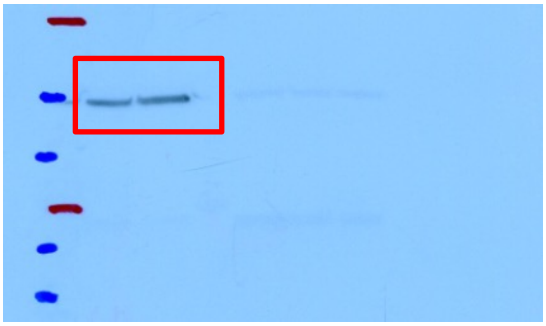

Full unedited blots for Supplementary Figure 5A

n 2

vinculin

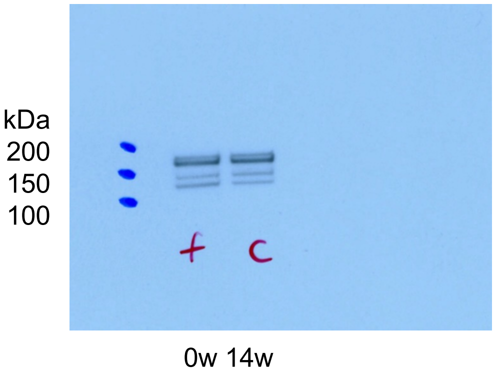

Cut between 75kDa and 100 kDa

tafazzin

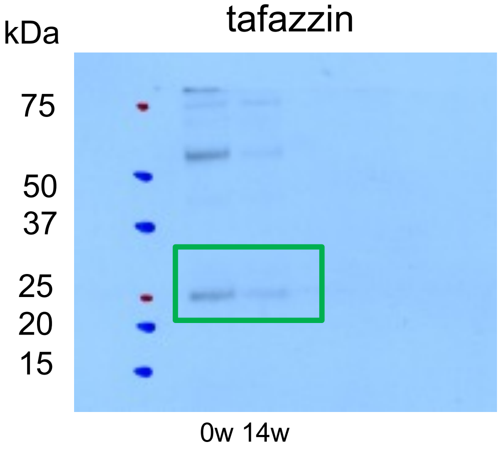

citrate synthase

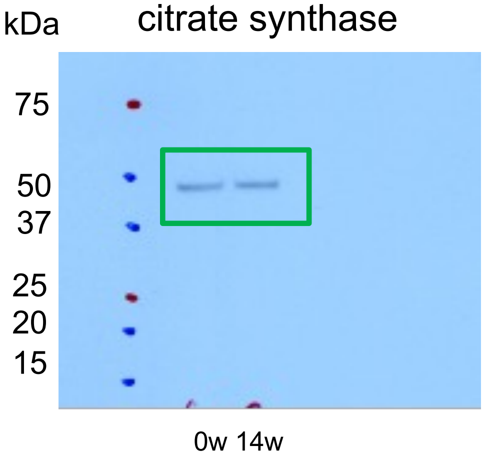

tubulin

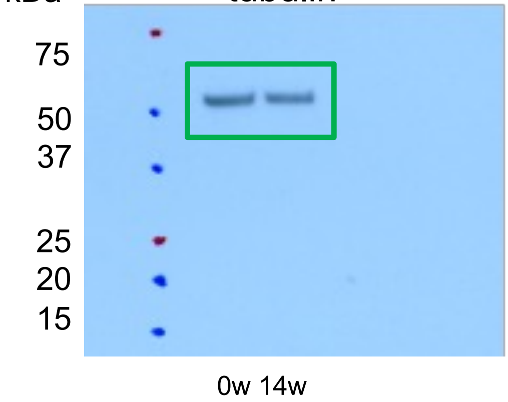

Full unedited blot for Supplementary Figure 5A

n 3

vinculin

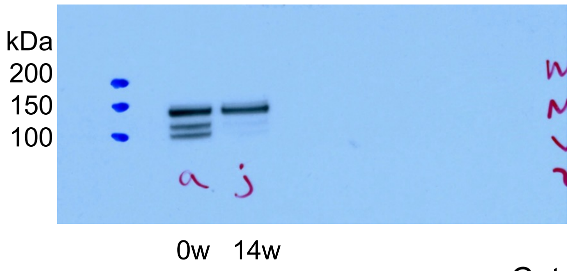

tafazzin

Cut between 75kDa and 100 kDa

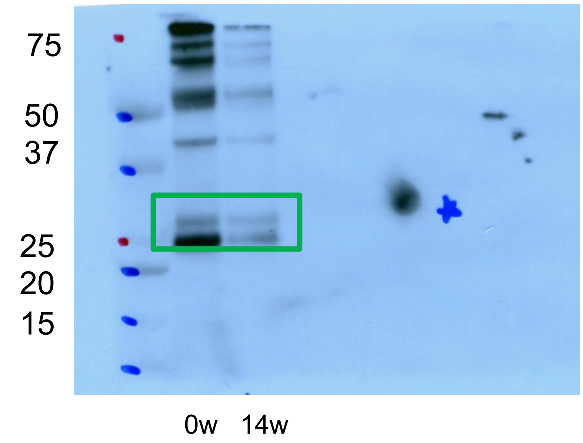

Citrate synthase

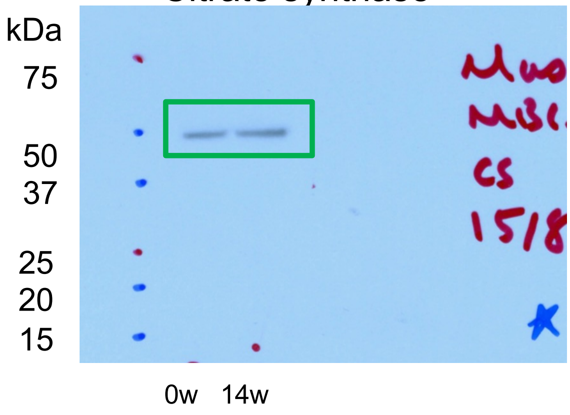

tubulin

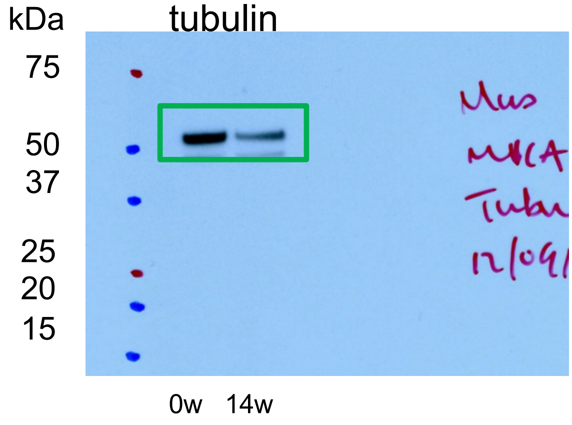

Full unedited blot for Supplementary Figure 5D

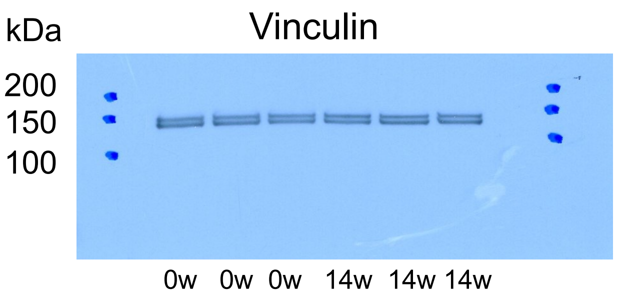

Cut between 100 and 75kDa

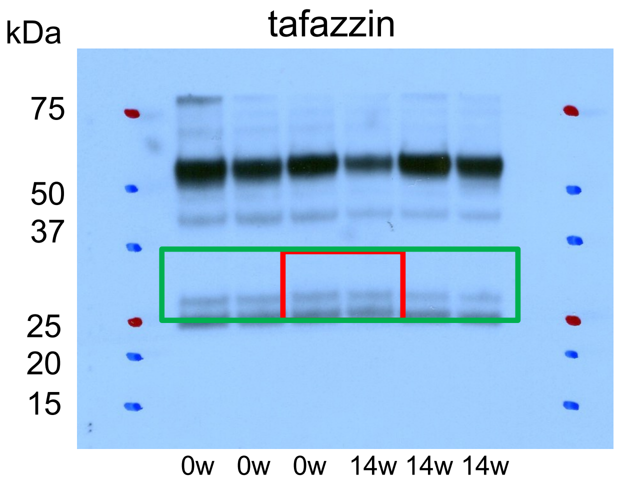

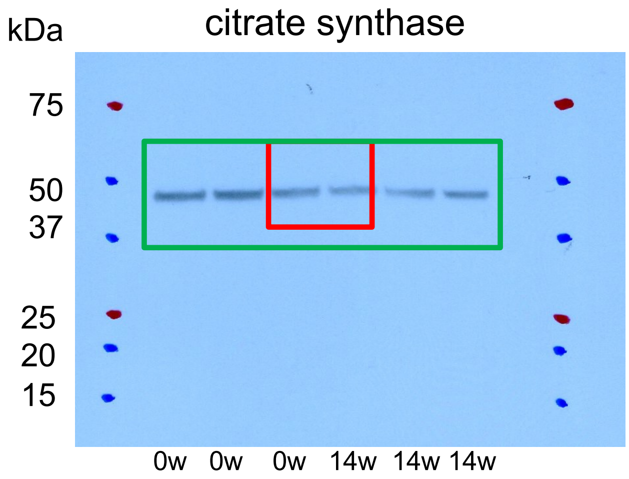

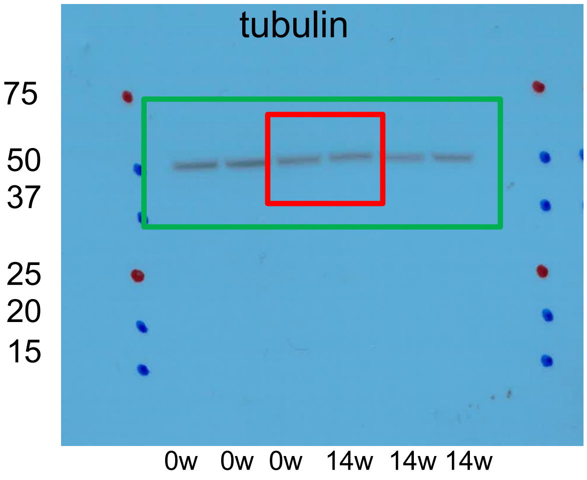

Full unedited blot for Supplementary Figure 7

n 1

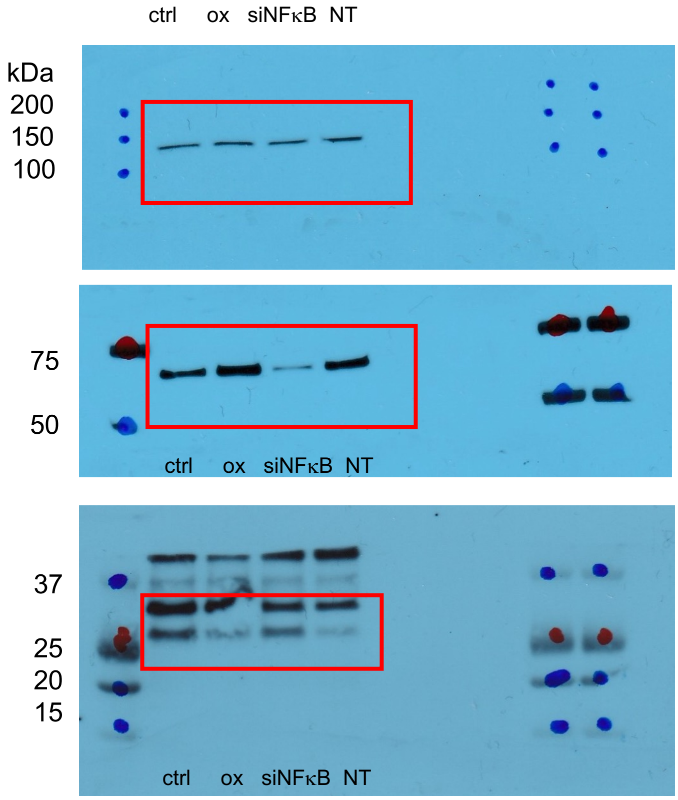

Vinculin

Cut between 100 and 75kDA markers

P-NFKB

Cut between 37 and 50 kDA markers

Taz

Cut membrane: same section as P-NFKB

NFKB

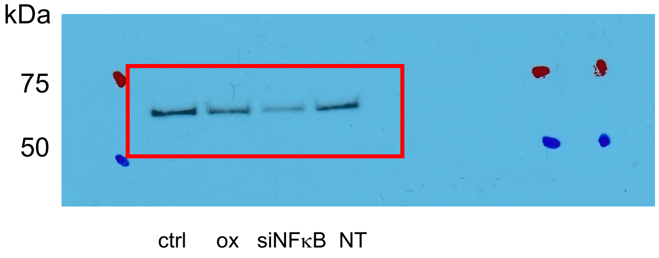

CS

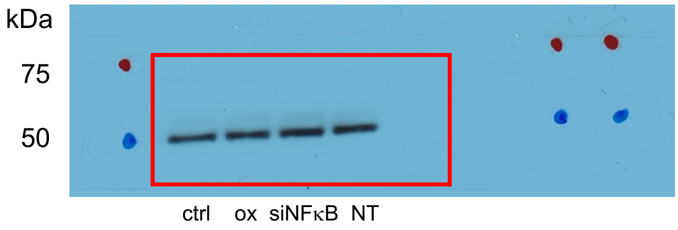

Full unedited blots for Supplementary Figure 7

n 2

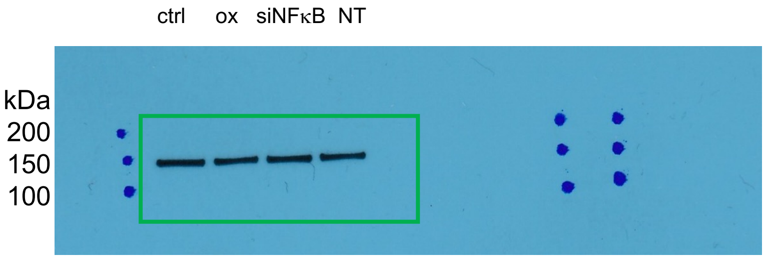

Vinculin

Cut between 100 and 75kDA markers

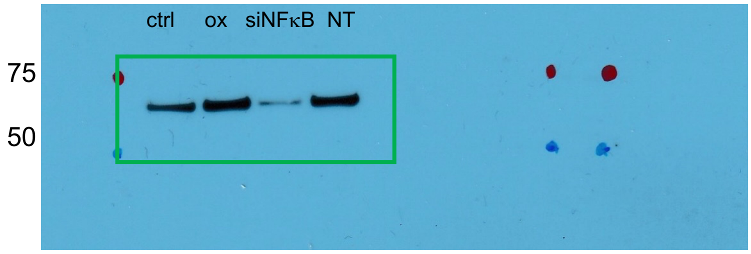

P-NFKB

Cut at 37kD marker

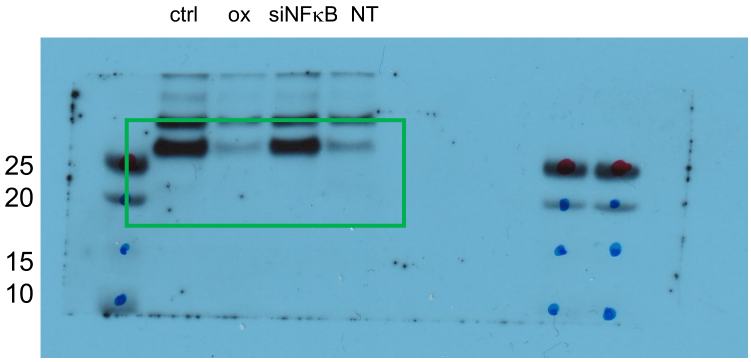

Taz

Cut membrane: same section as P-NFKB

NFKB

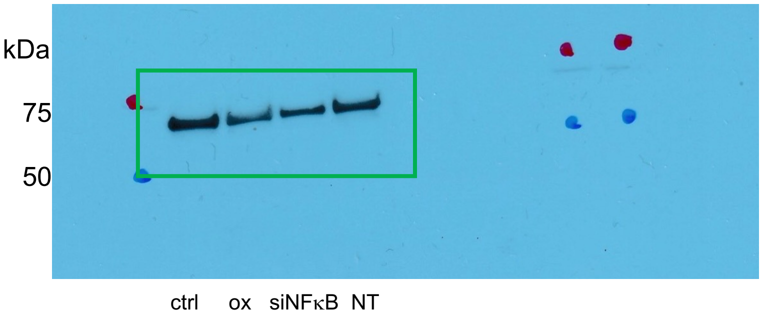

Citrate synthase

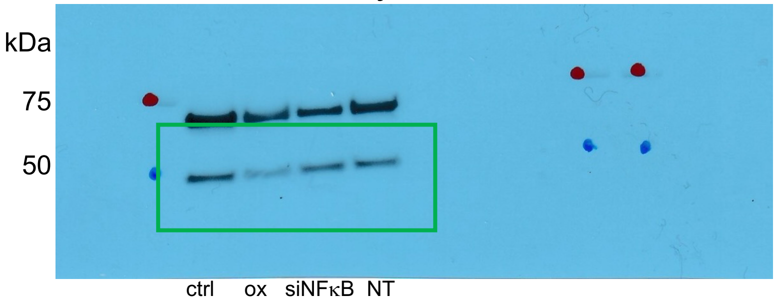

Full unedited blots for Supplementary Figure 7

n 3

Vinculin

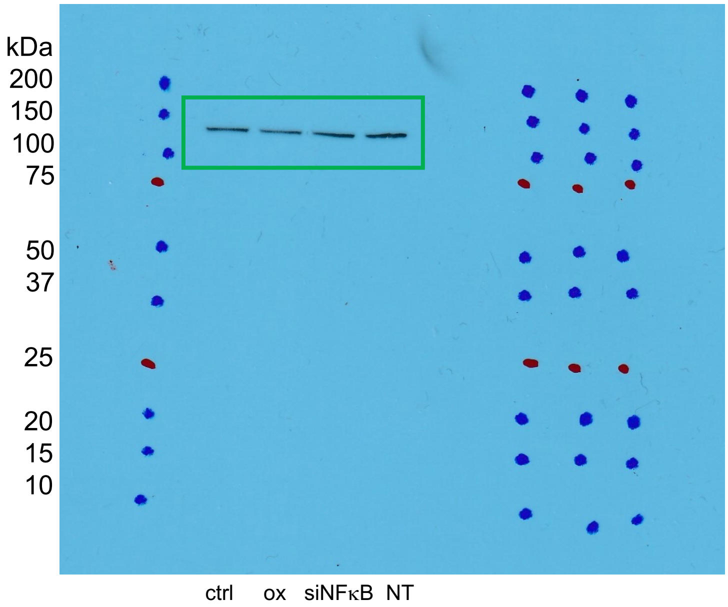

Cut membrane: same section as P-NFKB

NFKB

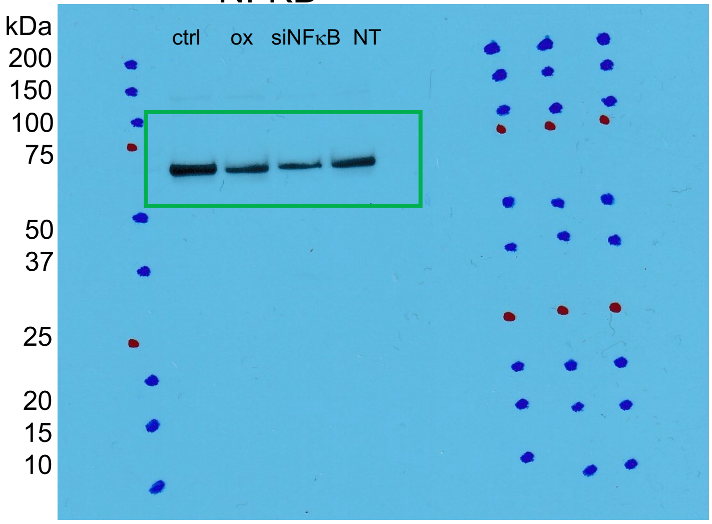

kDa Citrate synthase

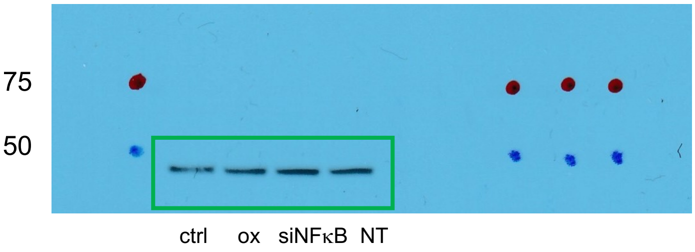

Cut between 100 and 75 kDA markers

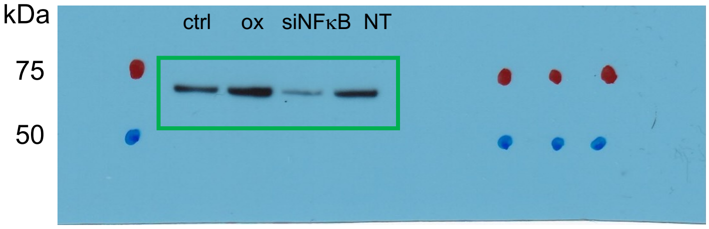

P-NFKB

Cut between 50 and 37 kDA markers

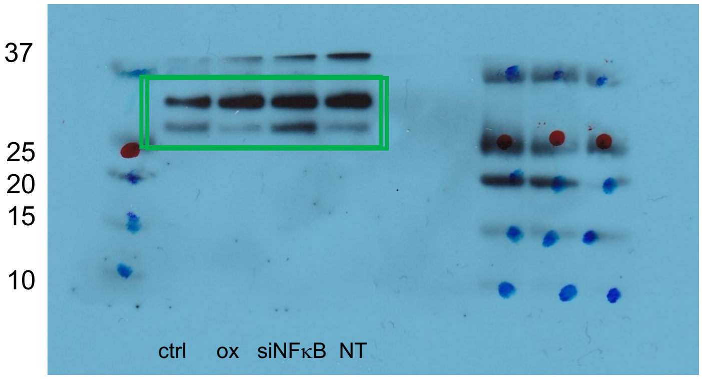

Taz

Full unedited blots for Supplementary Figure 7

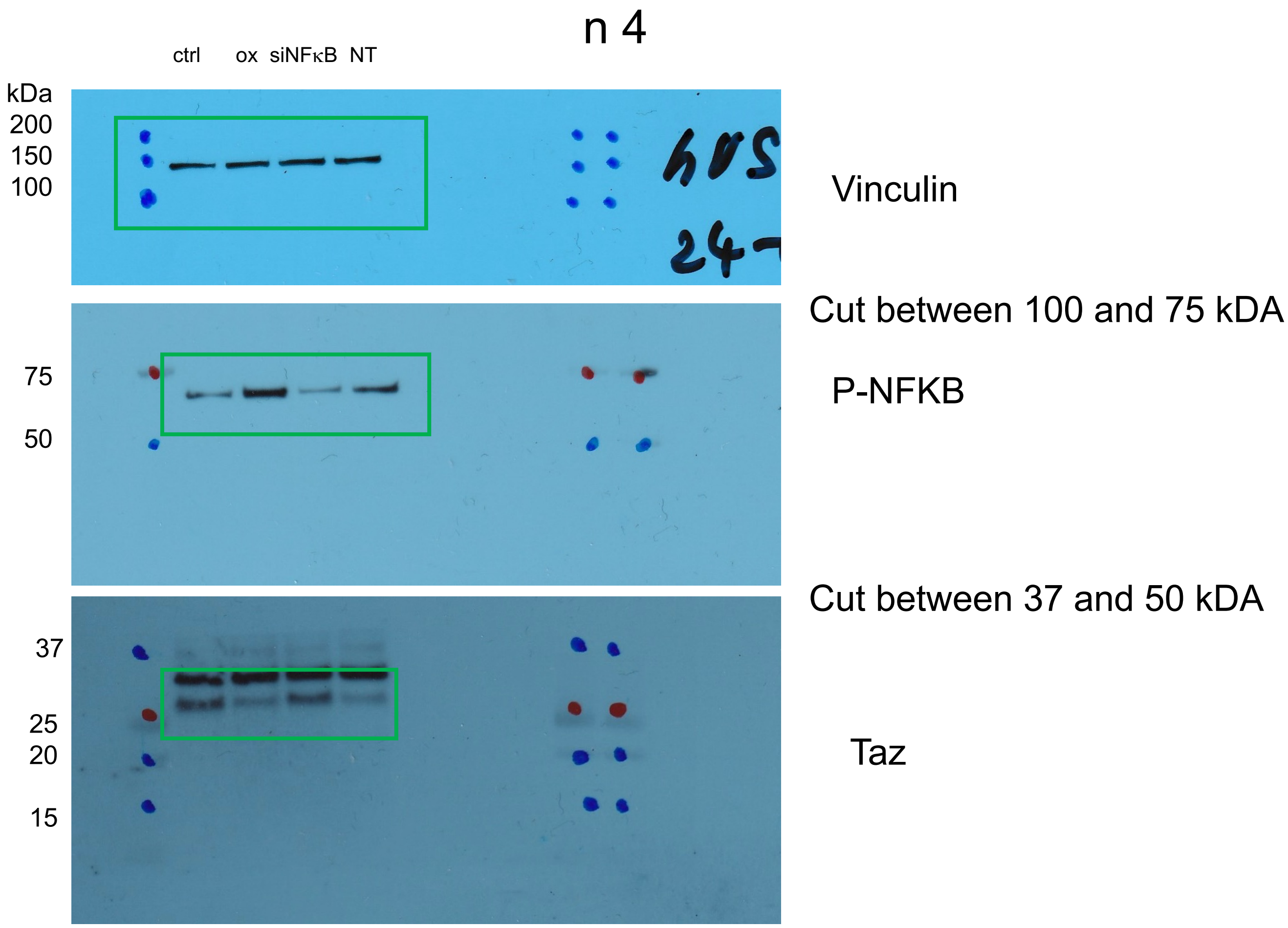

Cut membrane: same section as P-NFKB

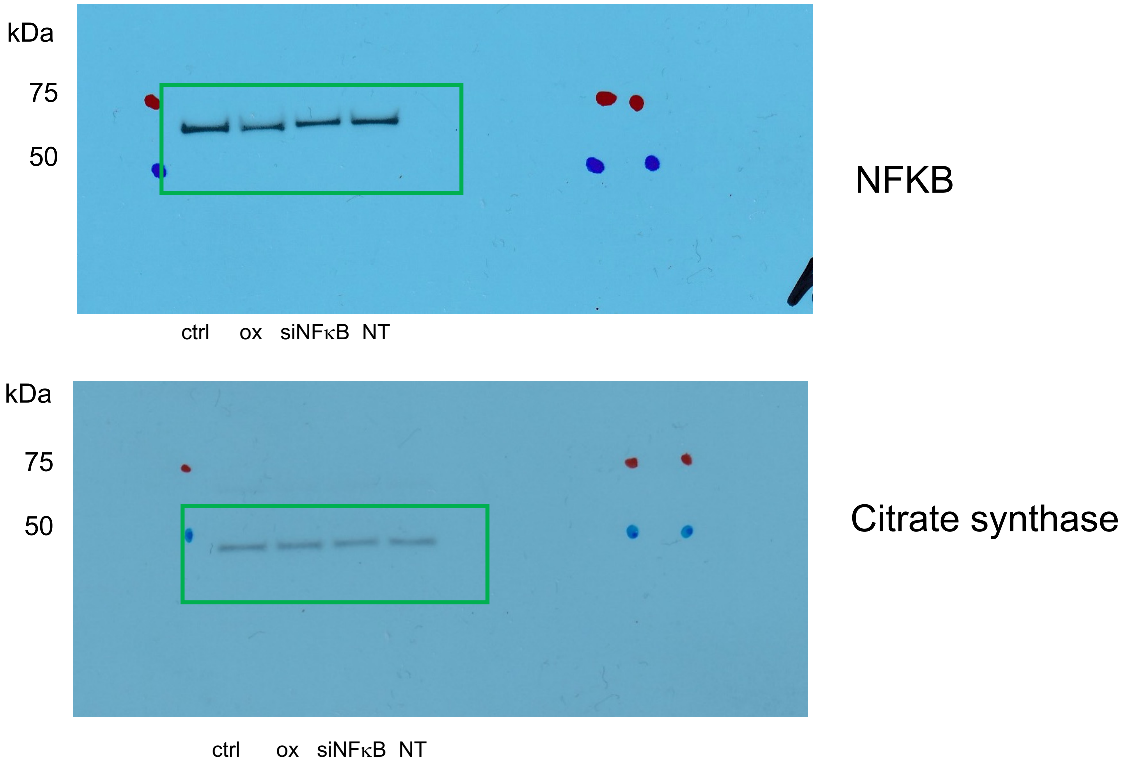

Full unedited blot for Supplementary Figure 9 left hand panel
n 1-2

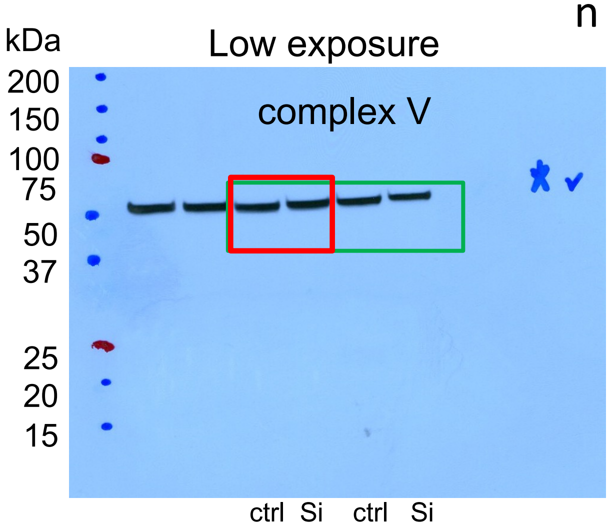

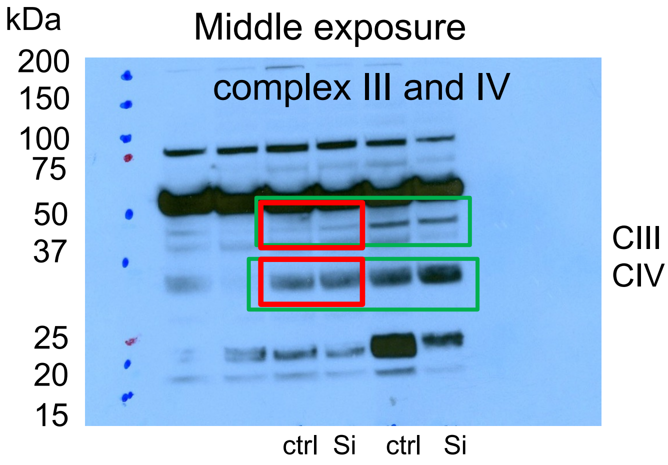

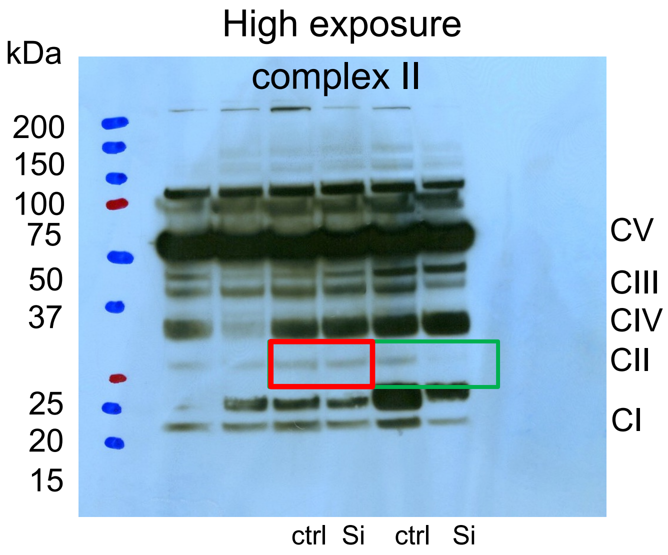

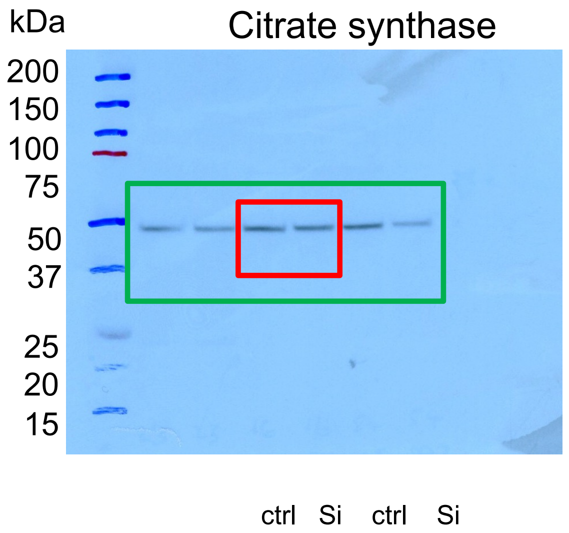

ctrl= control, Si= siRNA targeted against tafazzin

Same membrane as used for Figure 4A left hand panel (n1-2)

Full unedited blot for Supplementary Figure 9 left hand panel

n 3-4

Higher exposure complex III and IV

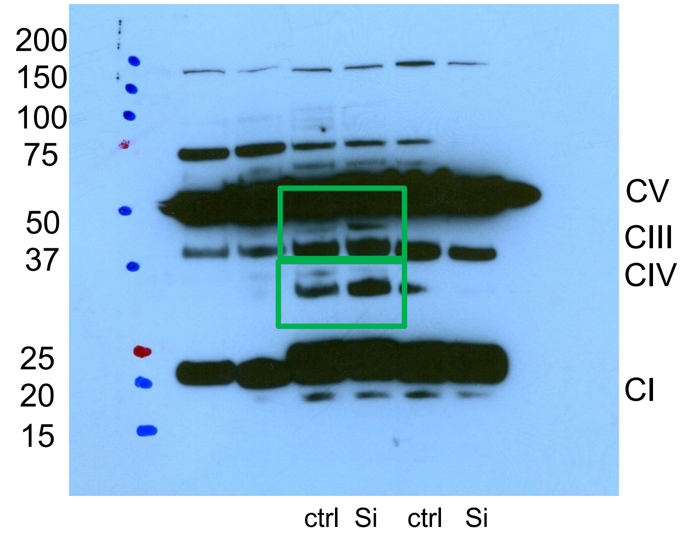

Cut between 75 and 100 & between 25 and 37

Low exposure complex V

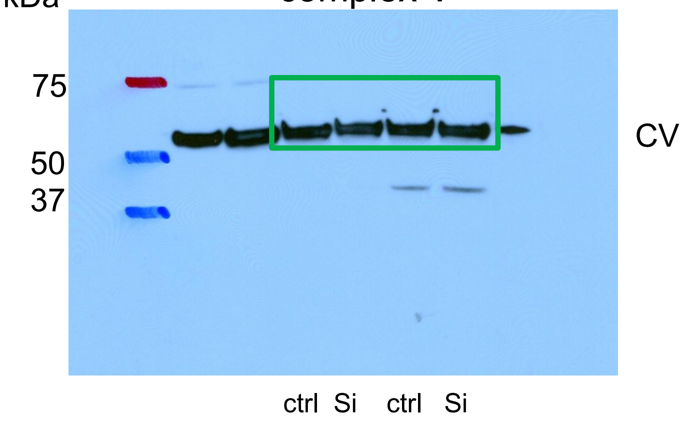

Citrate synthase

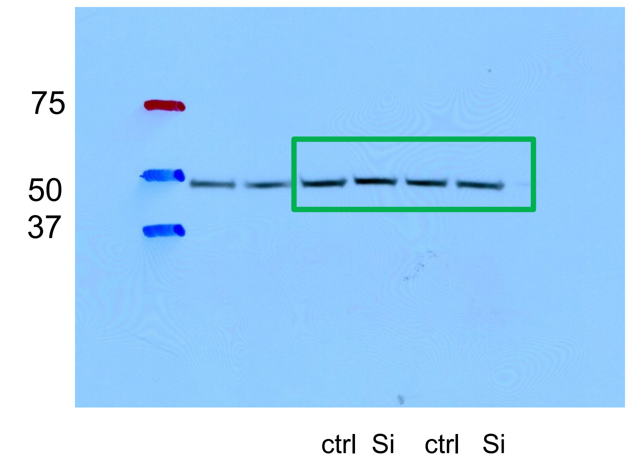

ctrl= control, Si= siRNA targeted against tafazzin

Same membrane as used for Figure 4A left hand panel (n3-4)

Full unedited blot for Supplementary Figure 9 left hand panel
n 5

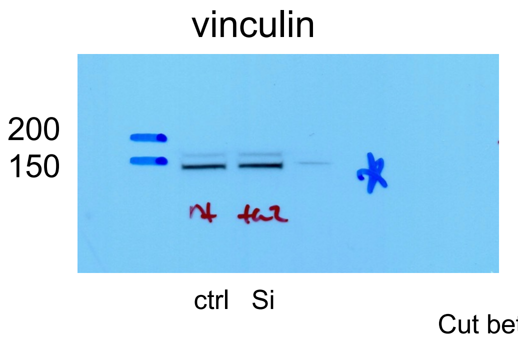

Cut between 100 and 150 kDa

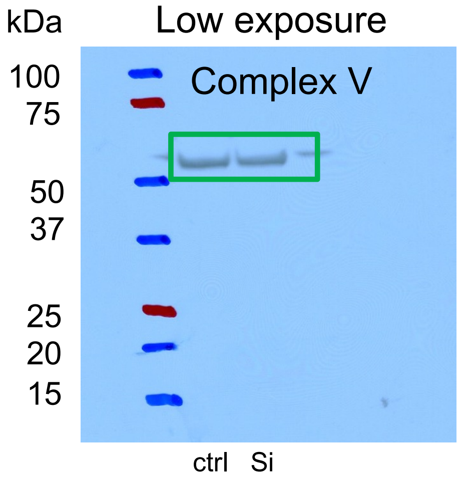

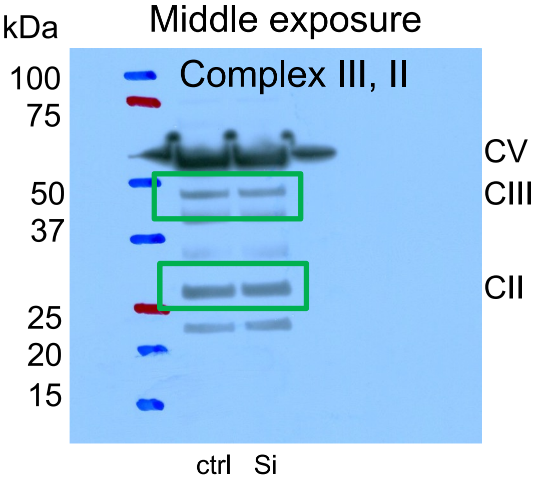

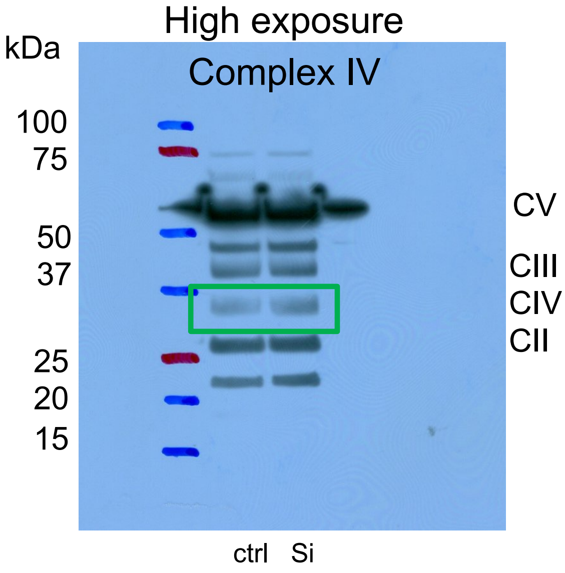

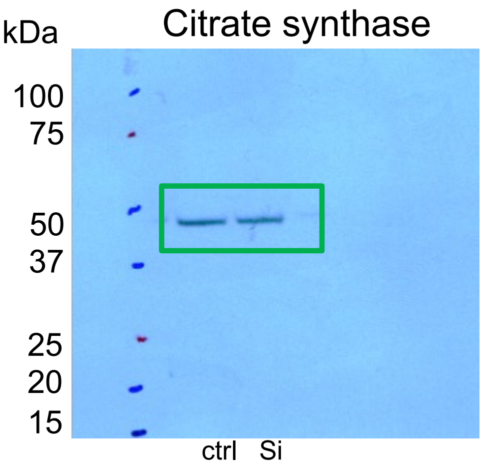

ctrl= control, Si= siRNA targeted against tafazzin

Same membrane as used for Figure 4A left hand panel (n5)

Full unedited blot for Supplementary Figure 9 right hand panel

Group 1

Low exposure
Complex V

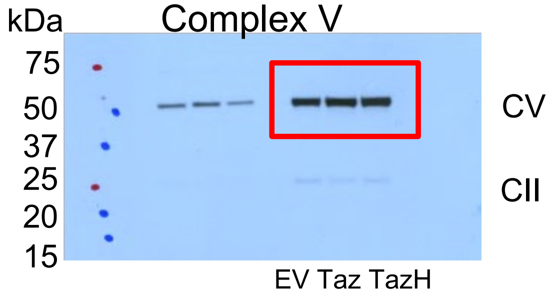

Middle exposure
Complex II

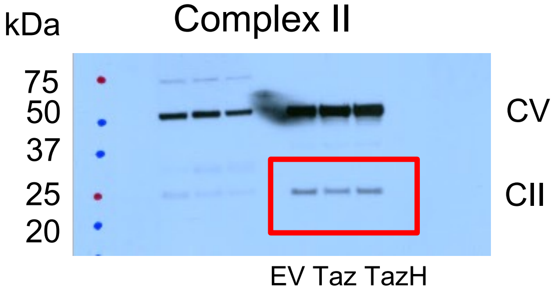

Higher exposure
Complex III

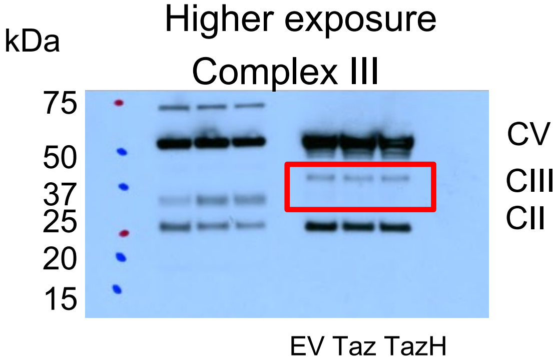

High exposure
Complex IV

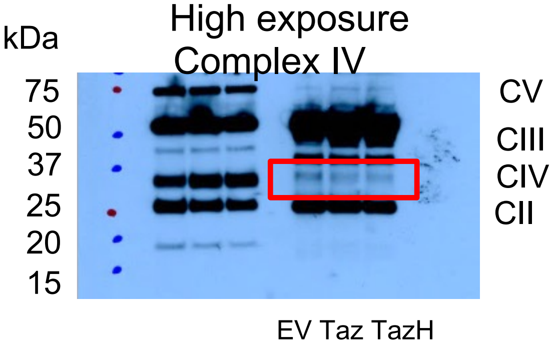

vinculin

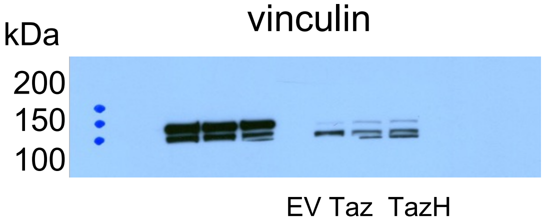

Cut between 75 and 100 kDa

Citrate synthase

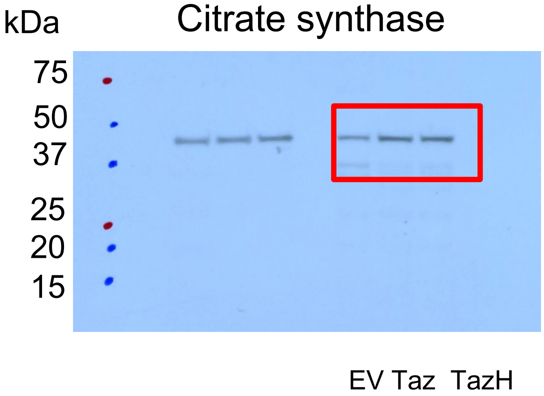

Same membrane as used for Figure 4A right hand panel (group1)

Full unedited blot for Supplementary Figure 9 right hand panel

Group 2

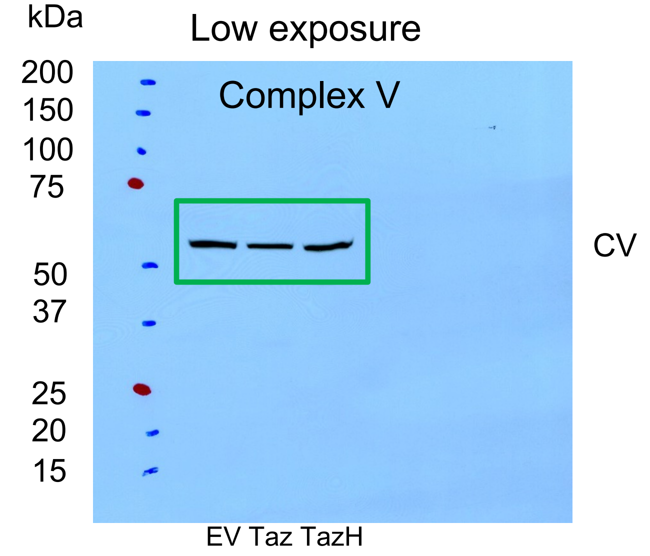

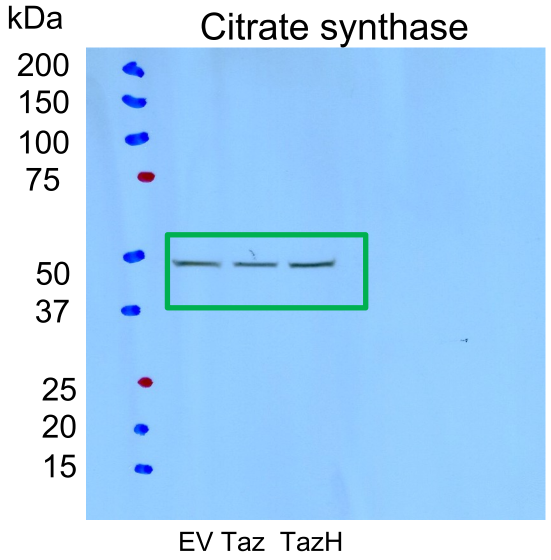

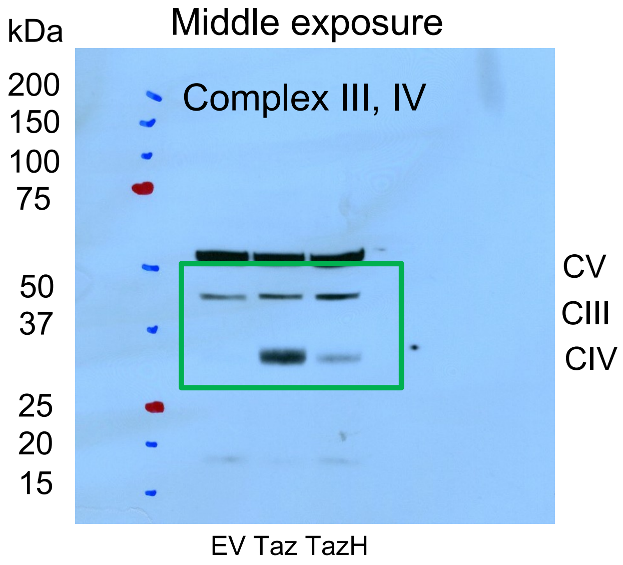

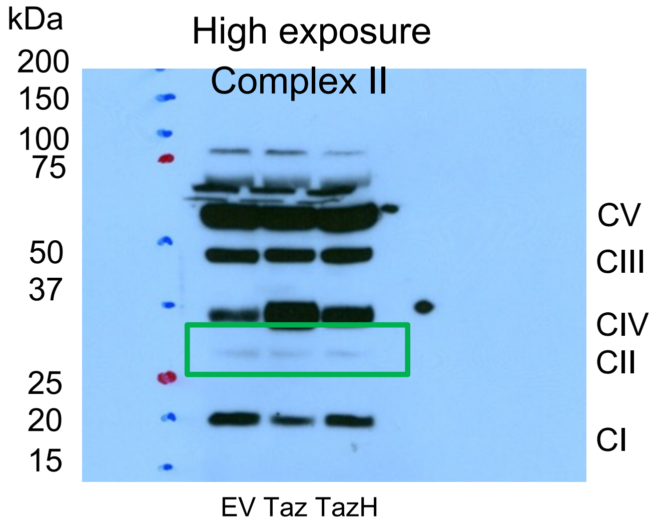

Same membrane as used for Figure 4A right hand panel (group 2)

Full unedited blot for Supplementary Figure 9 right hand panel

Group 3

Low exposure

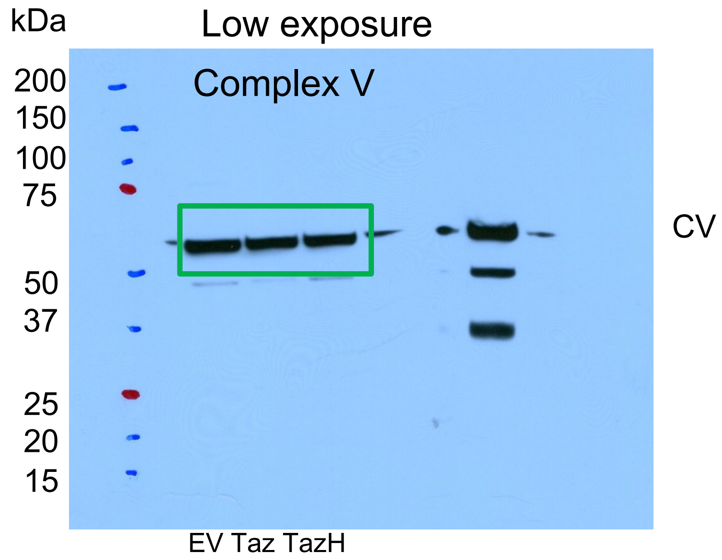

Citrate synthase

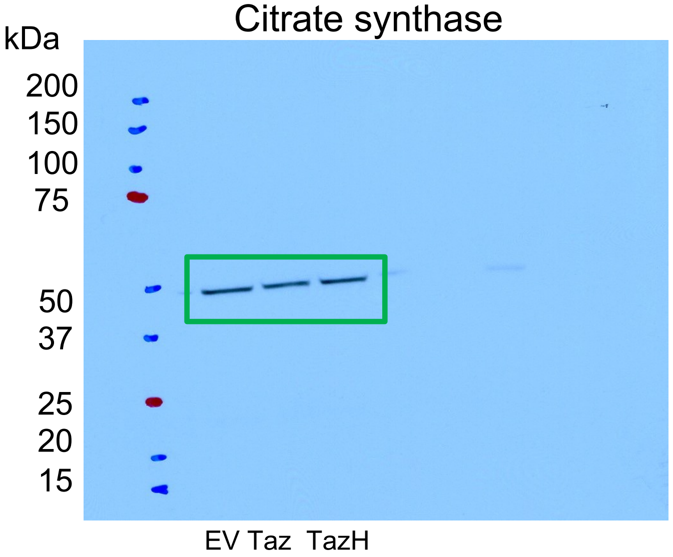

Middle exposure

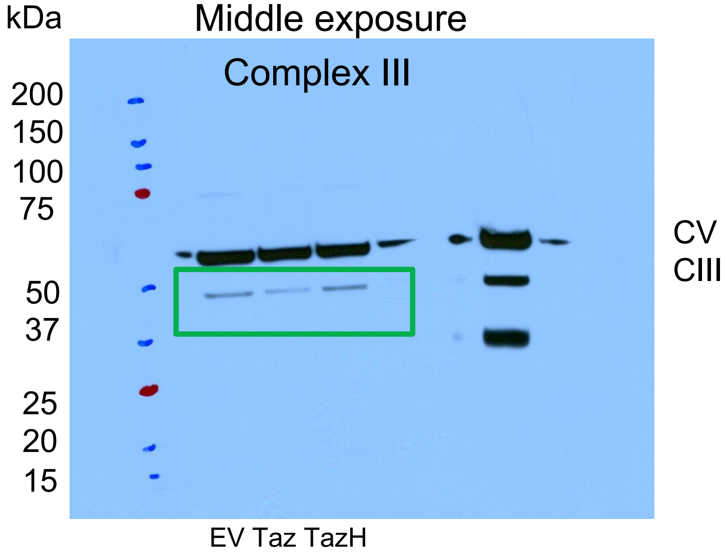

High exposure

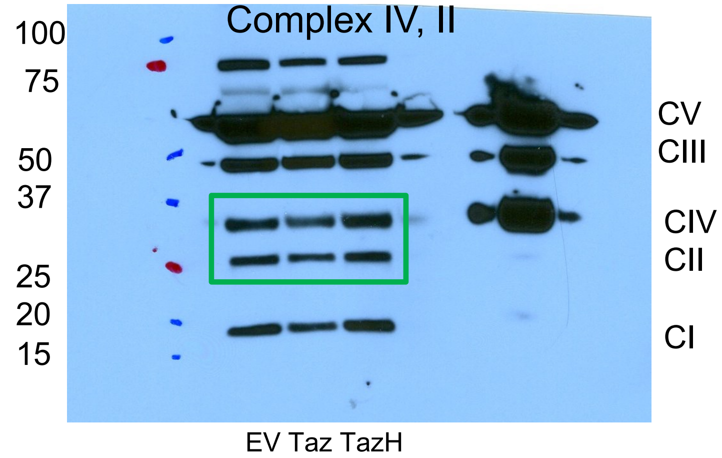

Same membrane as used for Figure 4A right hand panel (group 3)

Full unedited blot for Supplementary Figure 9 right hand panel

Group 4

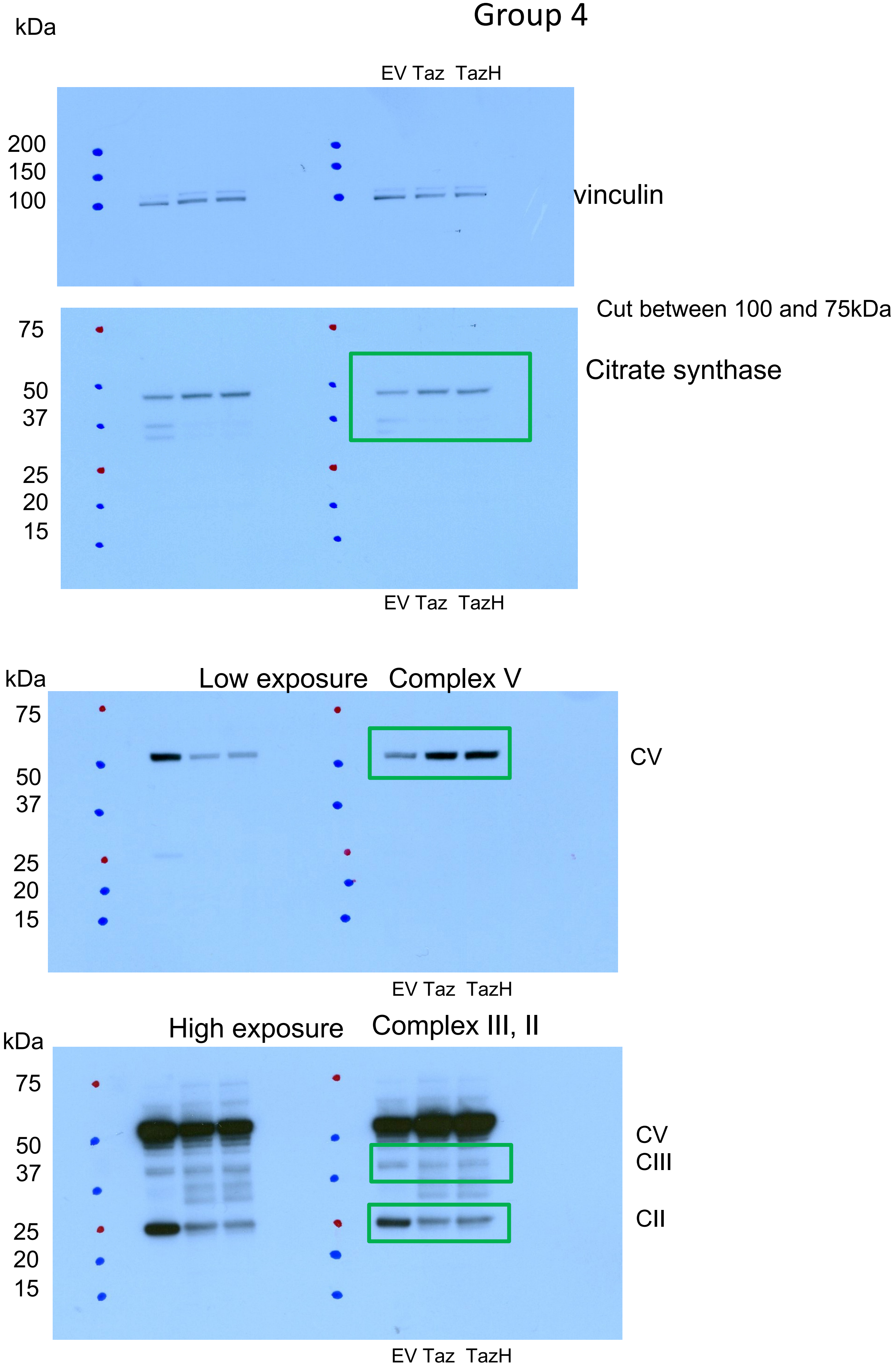

Same membrane as used for Figure 4A right hand panel (group 4)

Full unedited blot for Supplementary Figure 9 right hand panel

Group 5

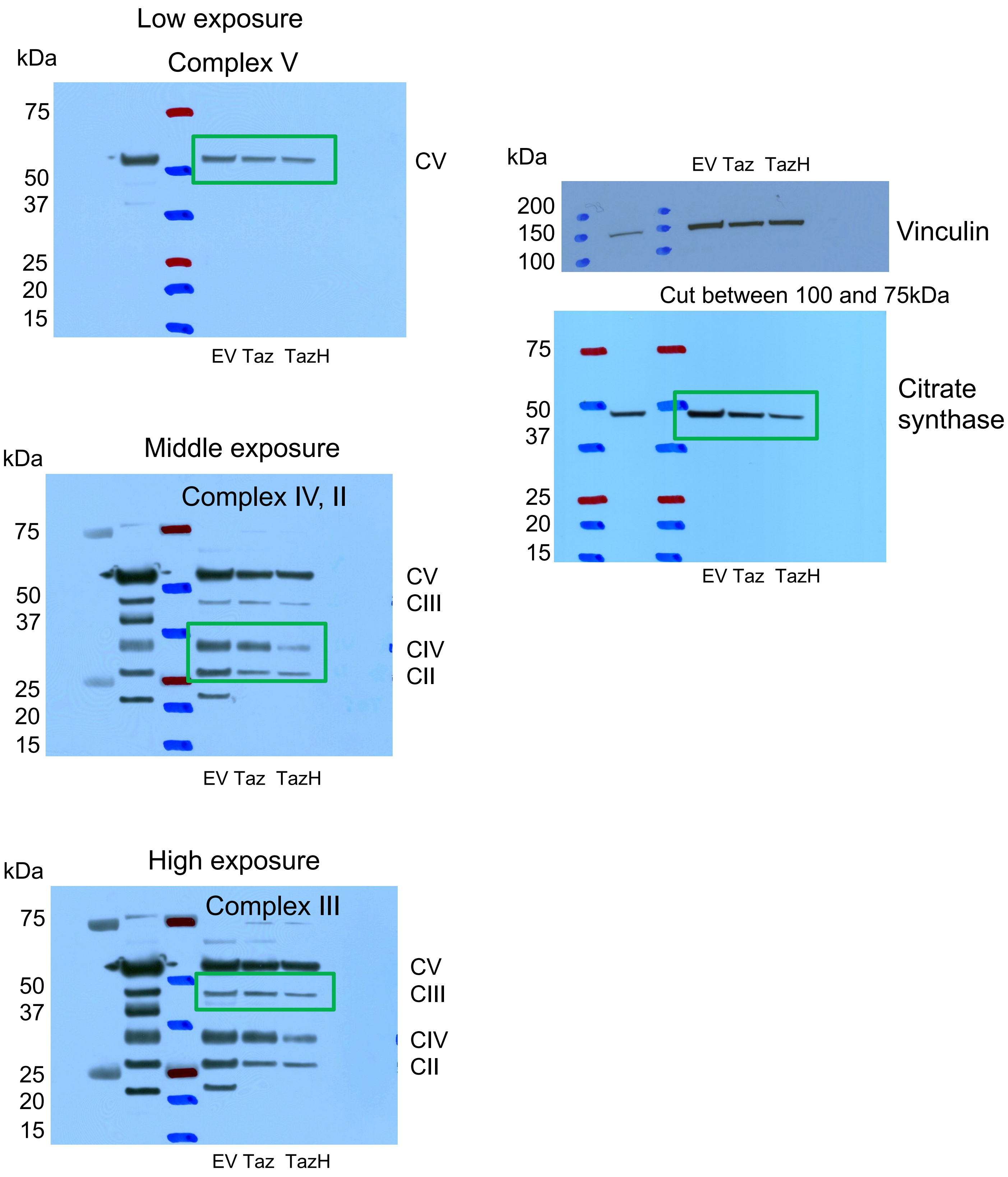

Same membrane as used for Figure 4A right hand panel (group 5)

Full unedited blots for Supplementary Figure 11
Group 1

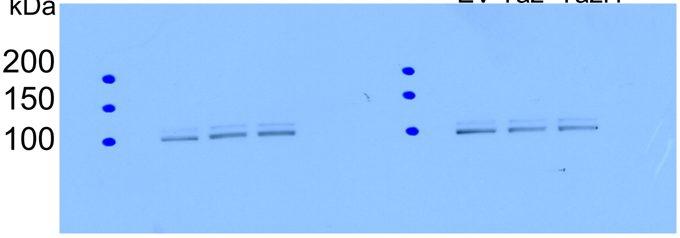

vinculin

Cut between 100 and 75kDa

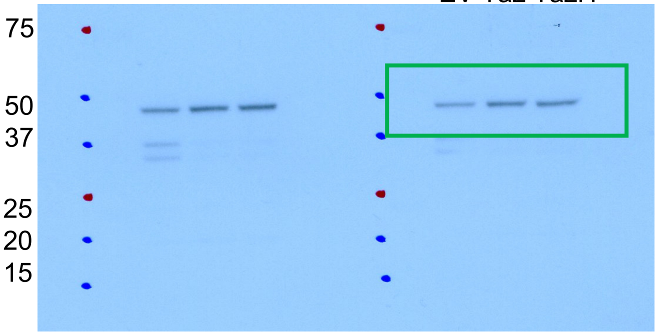

Citrate synthase

MFN2

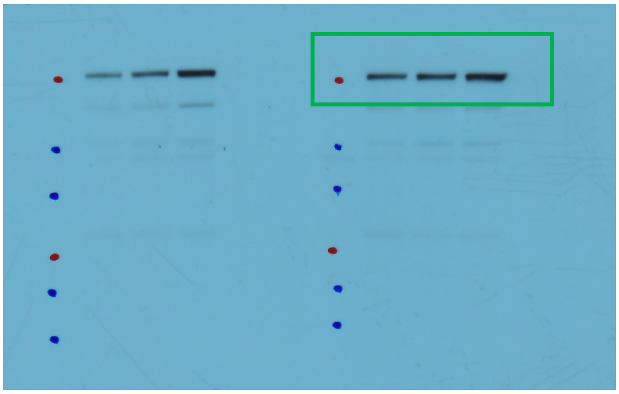

EV Taz TazH

Opa1

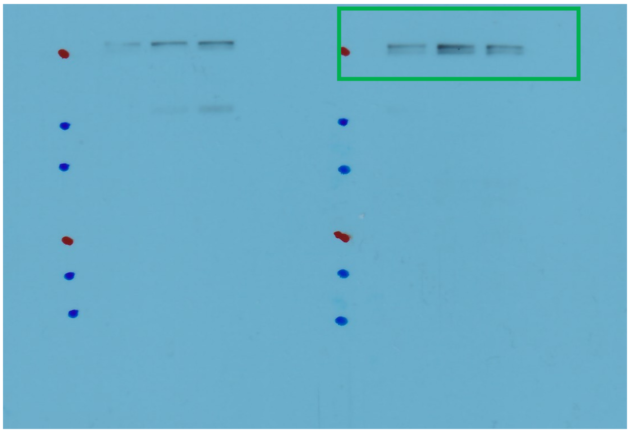

EV Taz TazH

Same membrane as used for Figure 4A right hand panel (group 4) and Supplemental Figure 9 right hand panel (group 4)

Full unedited blots for Supplementary Figure 11

Group 2

vinculin

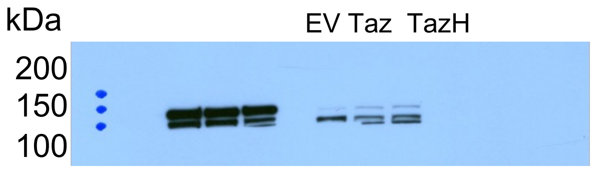

Cut at 100kDa

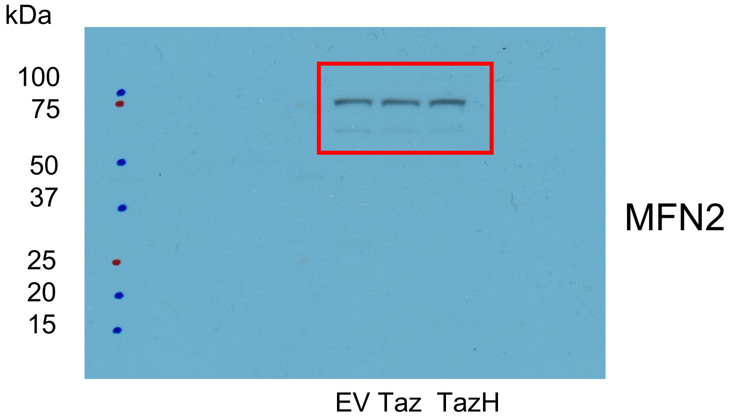

Citrate synthase

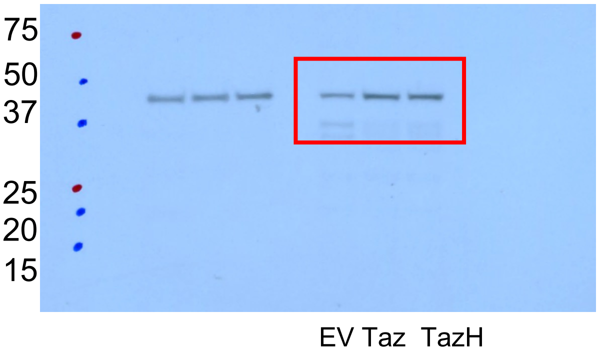

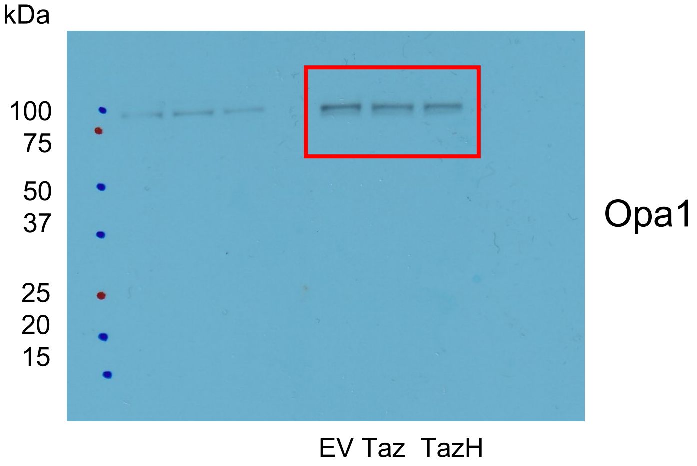

Same membrane as used for Figure 4A right hand panel (group1) and Supplemental Figure 9 (right hand panel, group1)

Full unedited blots for Supplementary Figure 11

Groups 1& 2:same samples ran on new gel

Drp1

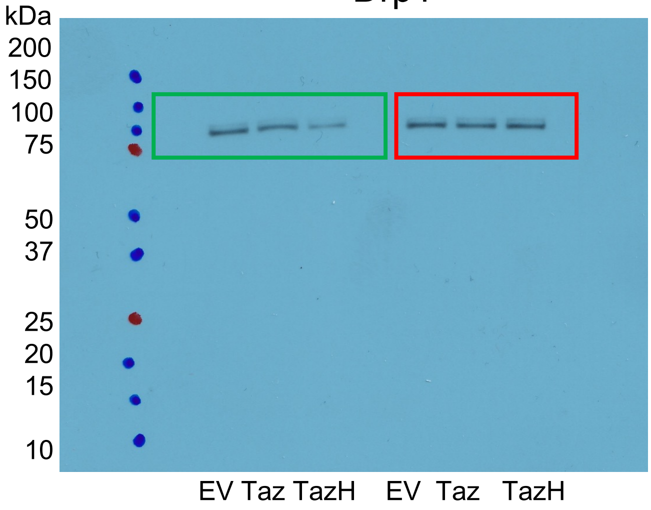

Tubulin

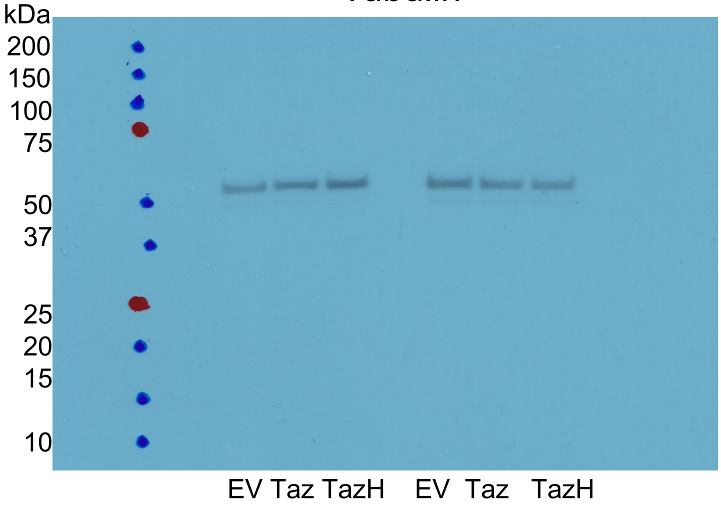

Fis1

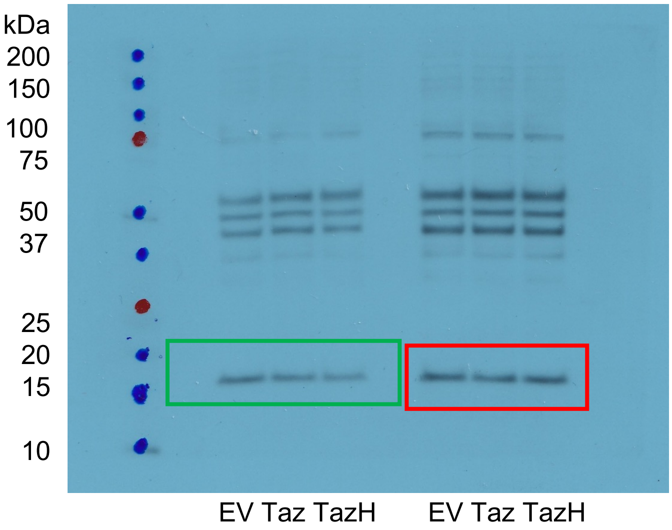

MFN1

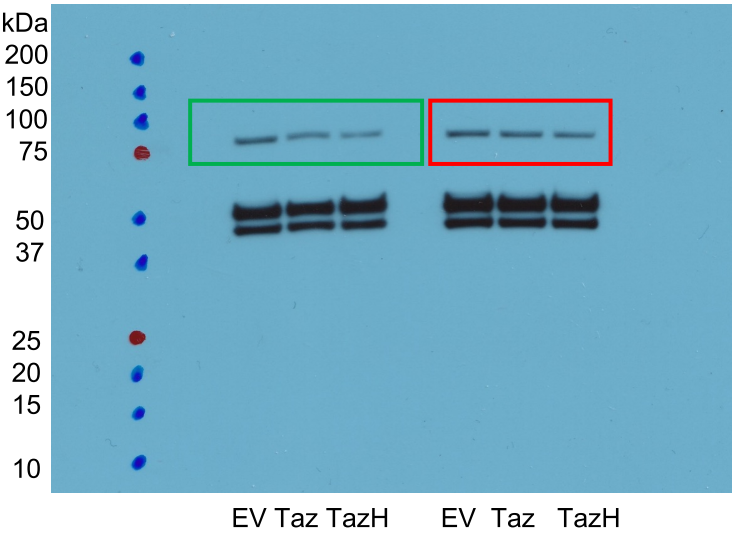

Citrate synthase

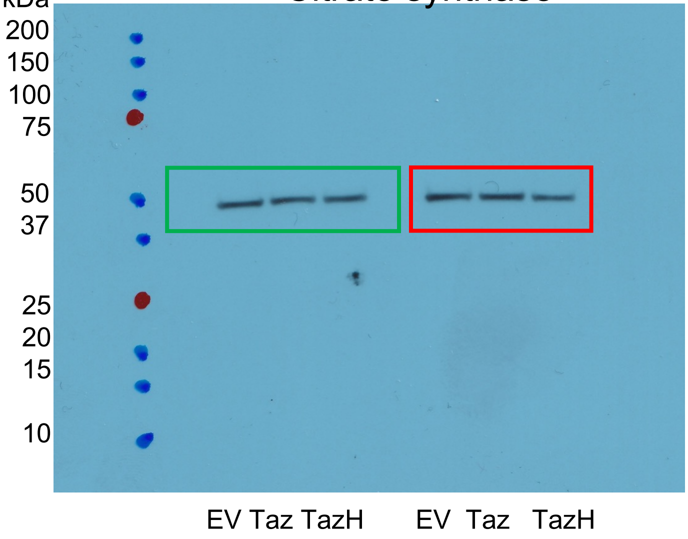

Full unedited blots for Supplementary Figure 11

Group 3

MFN1

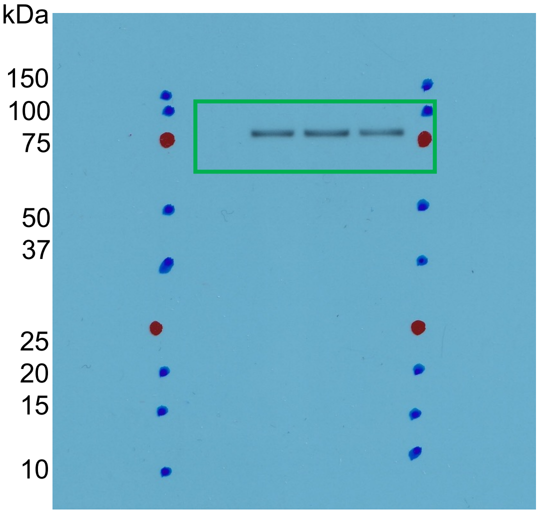

Citrate synthase

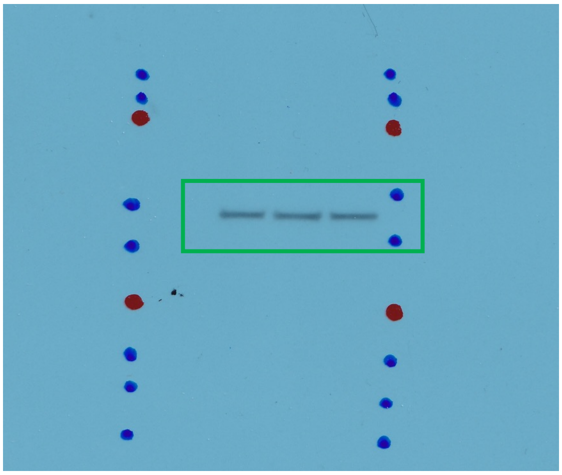

Drp1

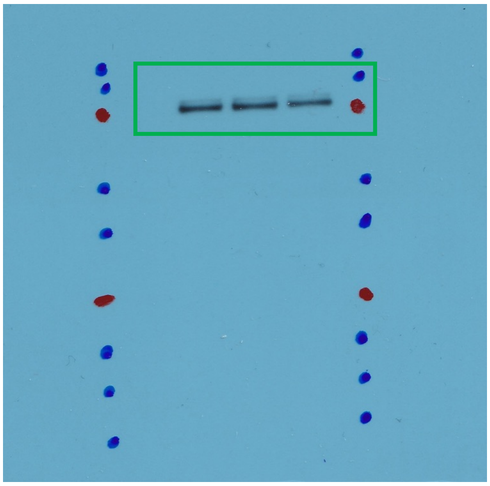

Tubulin

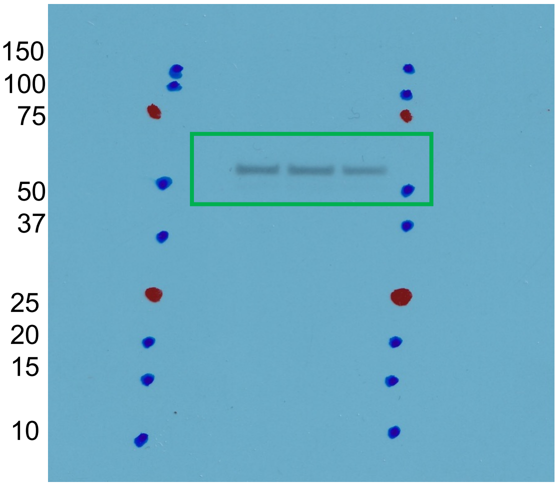

Fis1

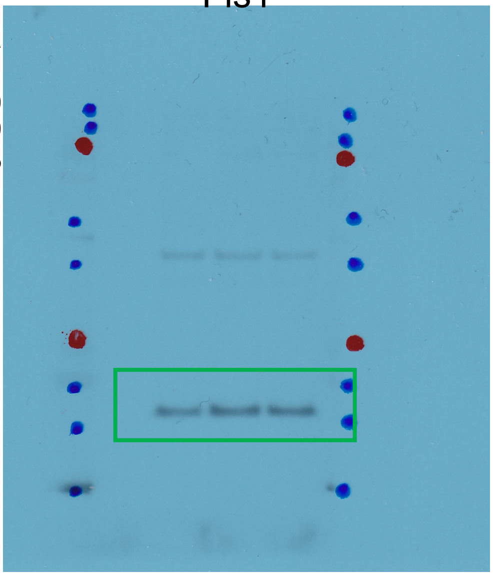

Full unedited blots for Supplementary Figure 11

Group 3:same samples ran on new gel

MFN2

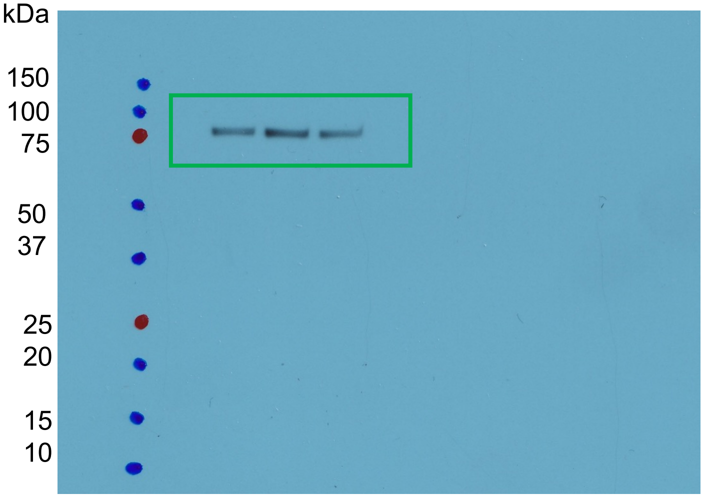

Opa1

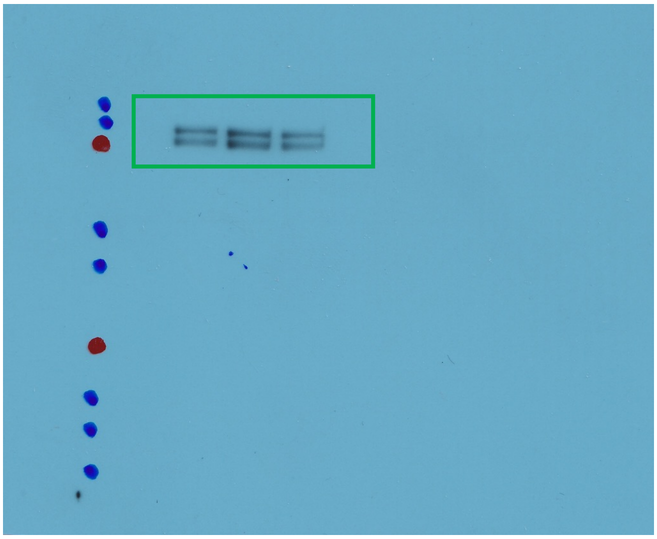

Tubulin

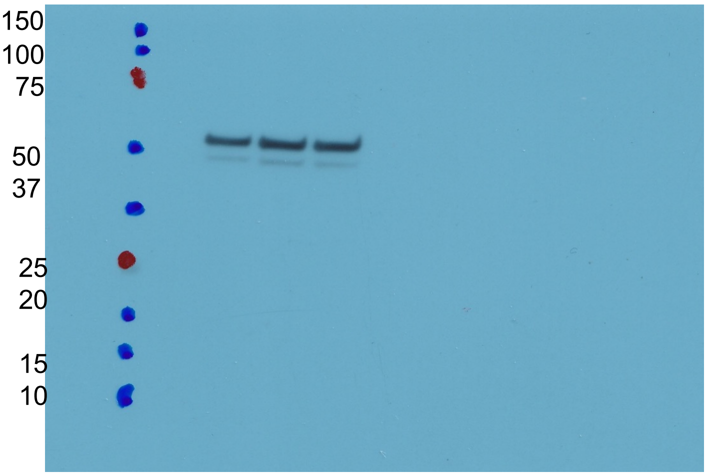

Citrate Synthase

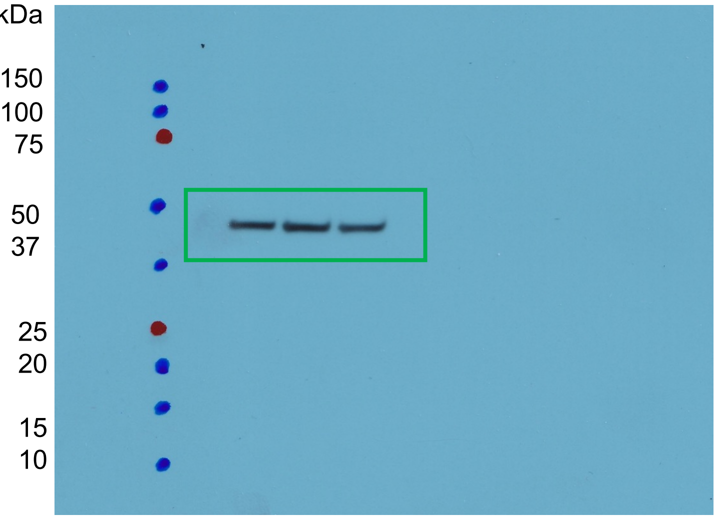

Full unedited blots for Supplementary Figure 11

Group 4

MFN1

kDa
200
150
100
75
50
37
25
20
15
10

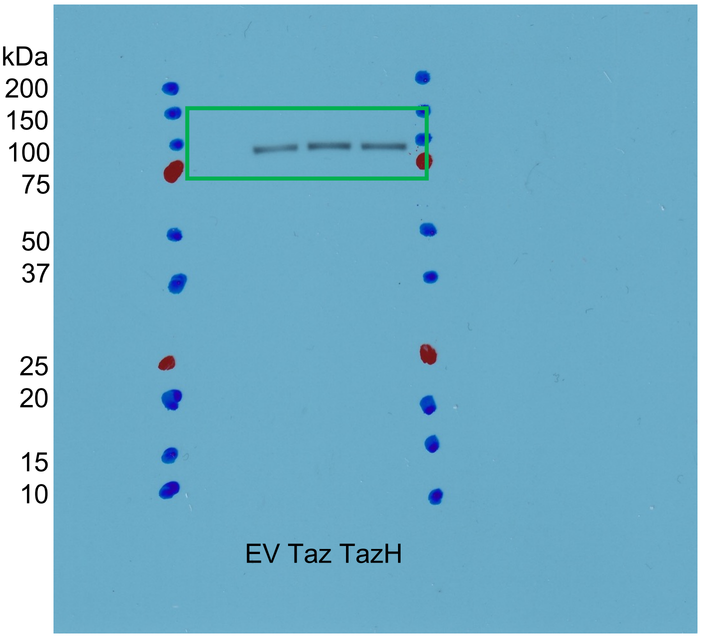

CS

kDa
200
150
100
75
50
37

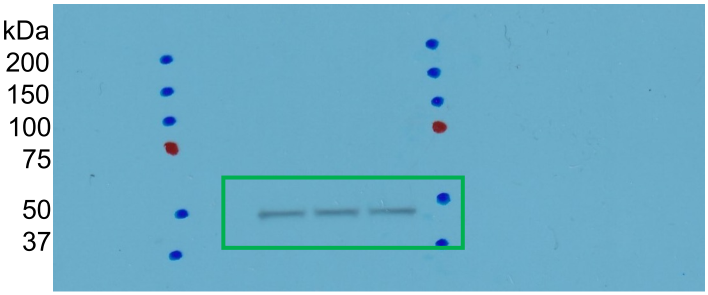

EV Taz TazH

Cut at 37kDa marker

kDa
37
25
20
15
10

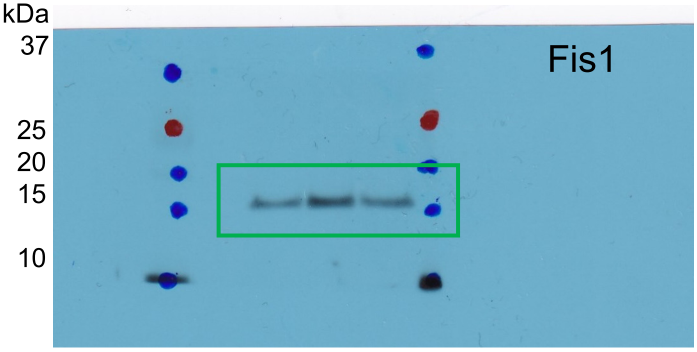

EV Taz TazH

Drp1

kDa
200
150
100
75
50
37
25
20
15
10

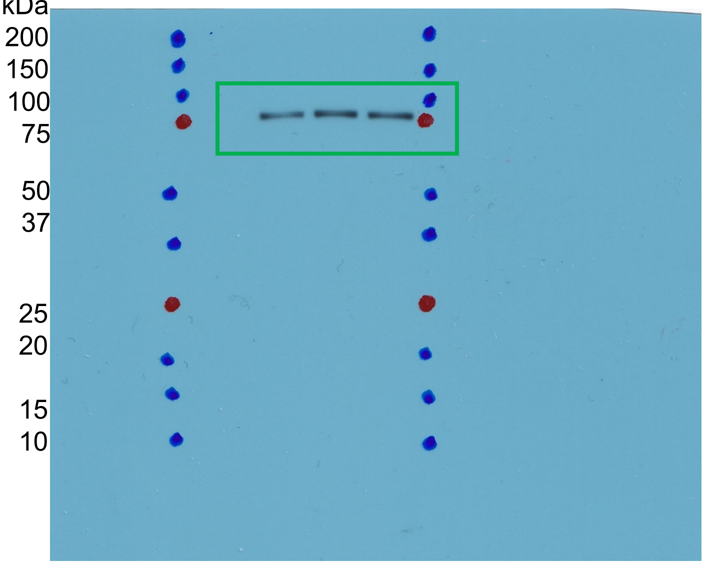

EV Taz TazH

Tubulin

kDa
200
150
100
75
50
37

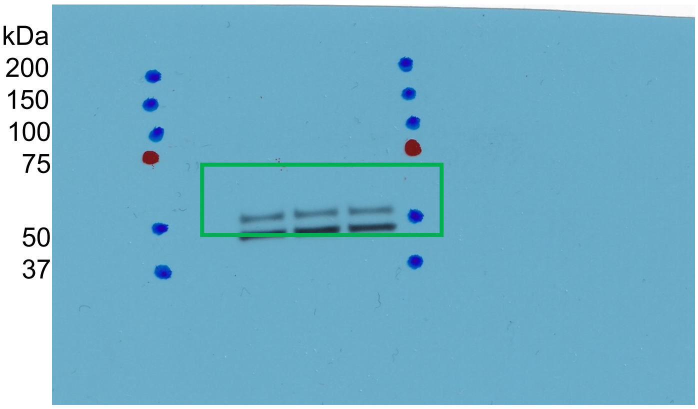

EV Taz TazH

Full unedited blots for Supplementary Figure 11

Group 4:same samples ran on new gel

MFN2

kDa
200
150
100
75
50
37
25
20
15
10

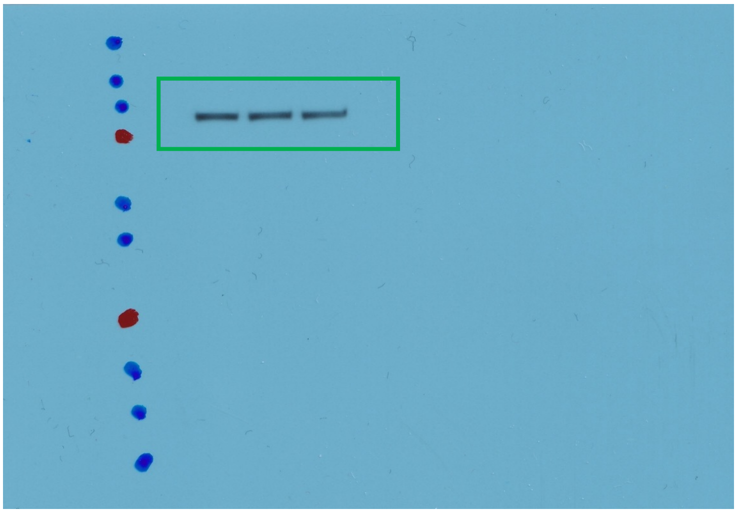

EV Taz TazH

Opa1

kDa
200
150
100
75
50
37
25
20
15
10

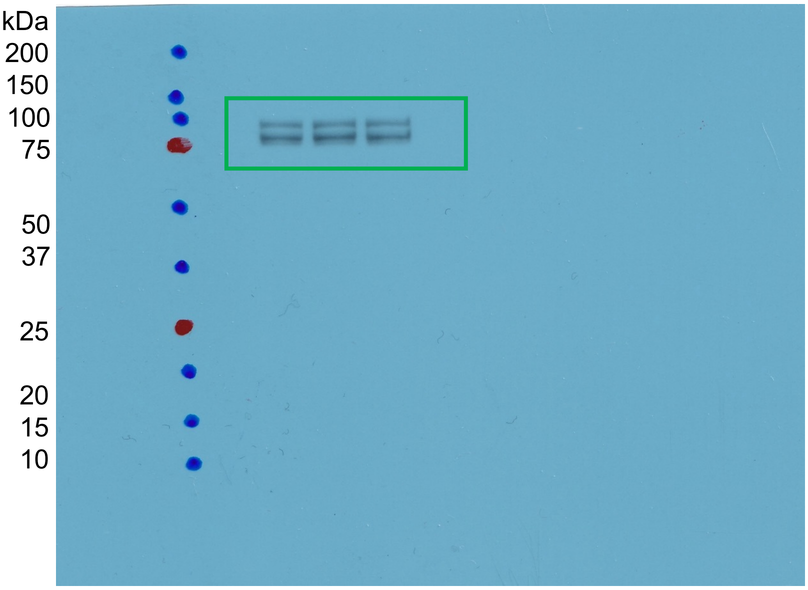

EV Taz TazH

Cut above 37kDa marker

CS

kDa
200
150
100
75
50
37

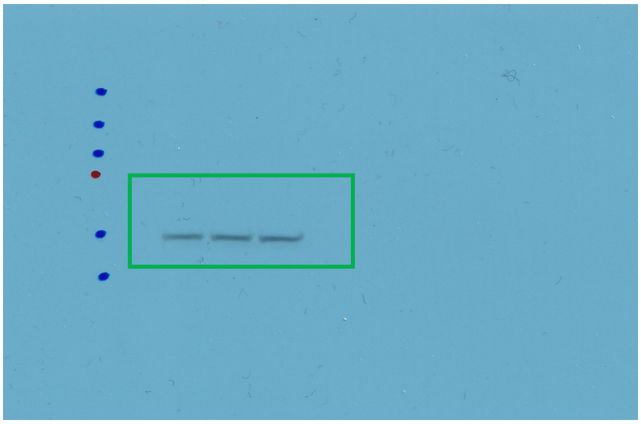

EV Taz TazH

Tubulin

kDa
200
150
100
75
50
37

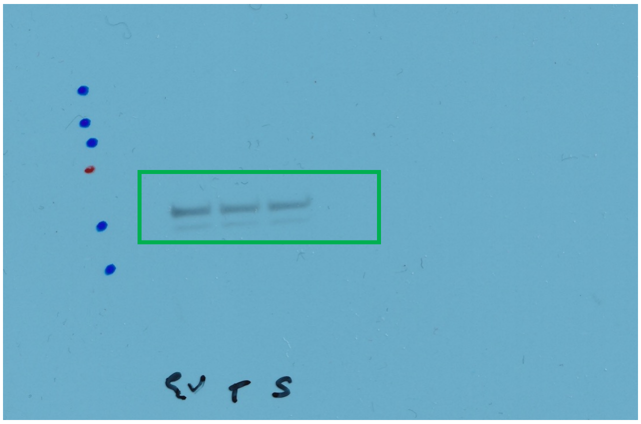

EV Taz TazH

Full unedited blots for Supplementary Figure 14B n 1

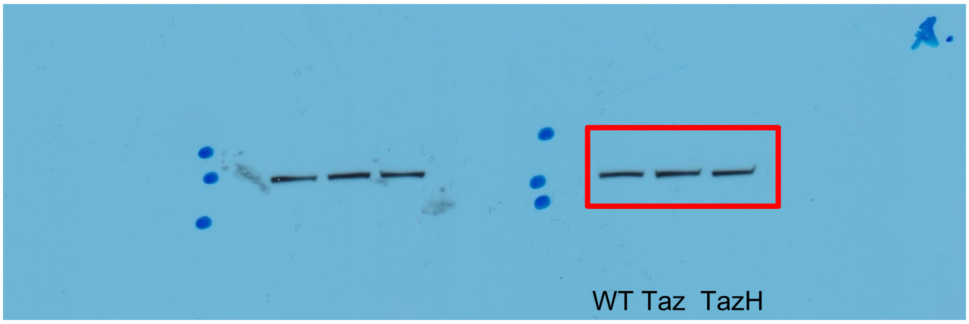

Cut between 75 and 100 kDa

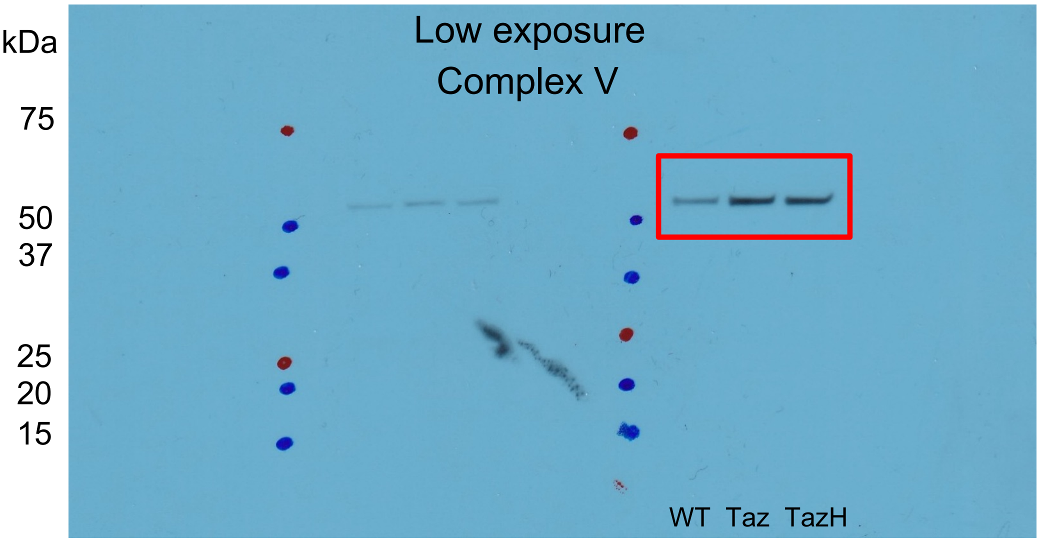

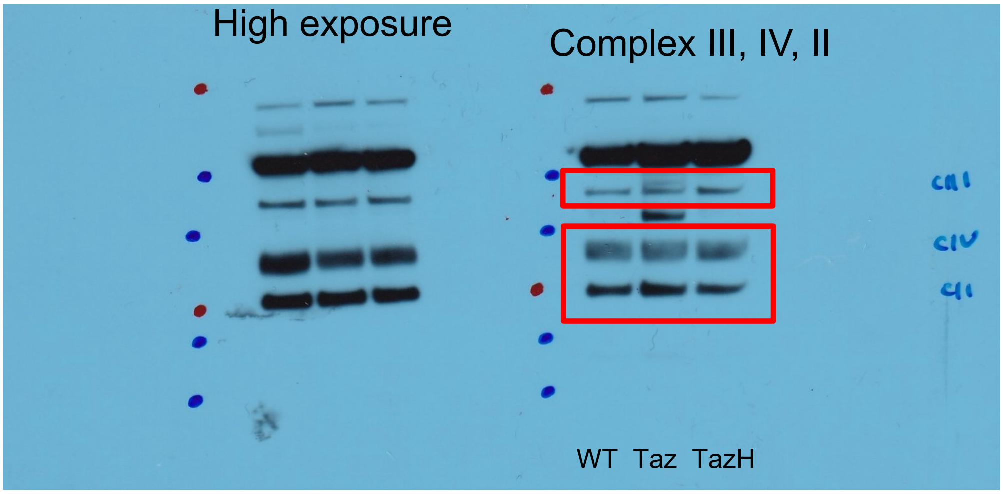

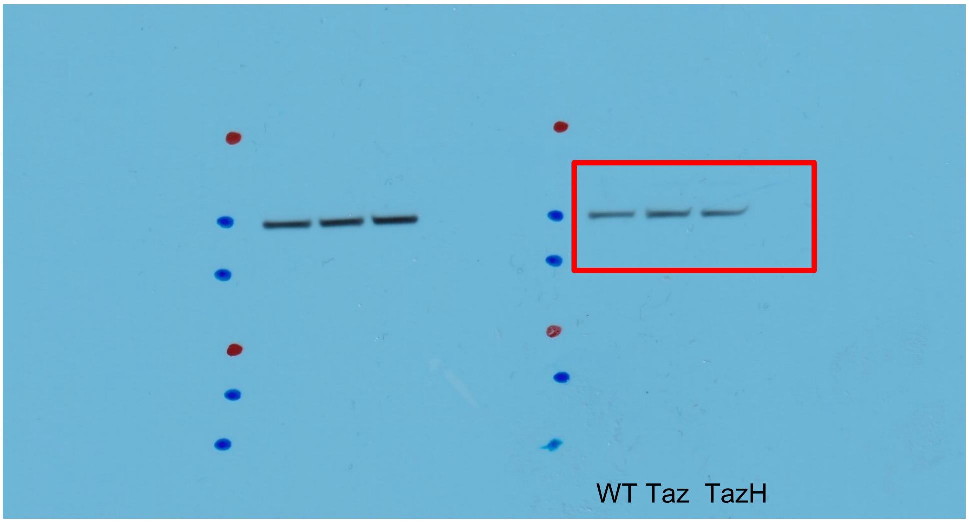

Same membrane as used for Figure 5D (n1)

Full unedited blots for Supplementary Figure 14B

n 2

Vinculin

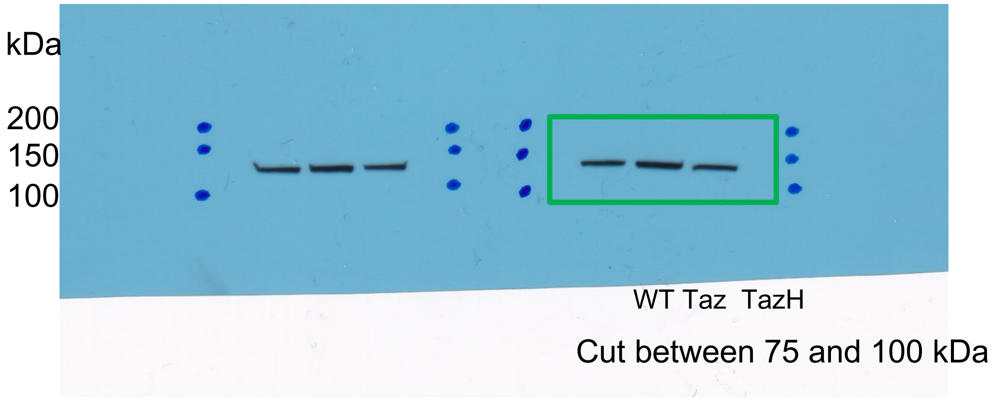

Low exposure

Complex V

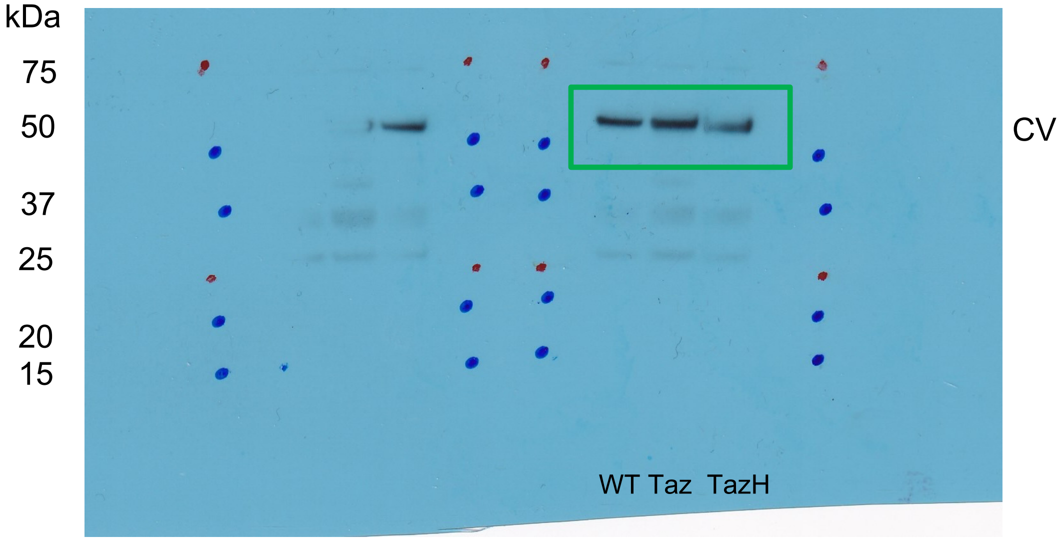

Middle exposure

Complex IV, II

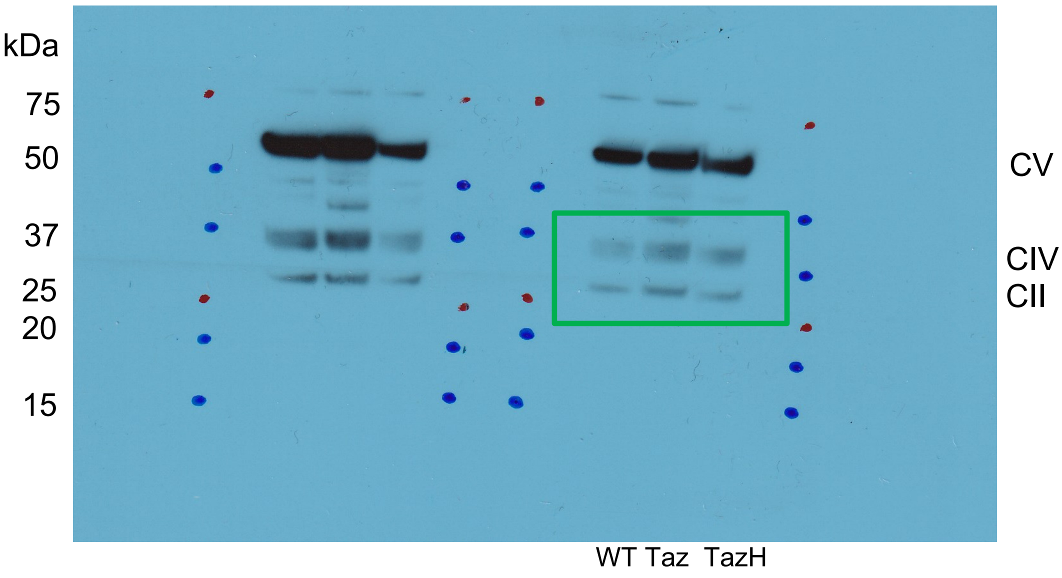

Same membrane as used for Figure 5D (n2)

Full unedited blots for Supplementary Figure 14B
n 2 ctd

Cut between 75 and 100 kDa

High exposure
Complex III

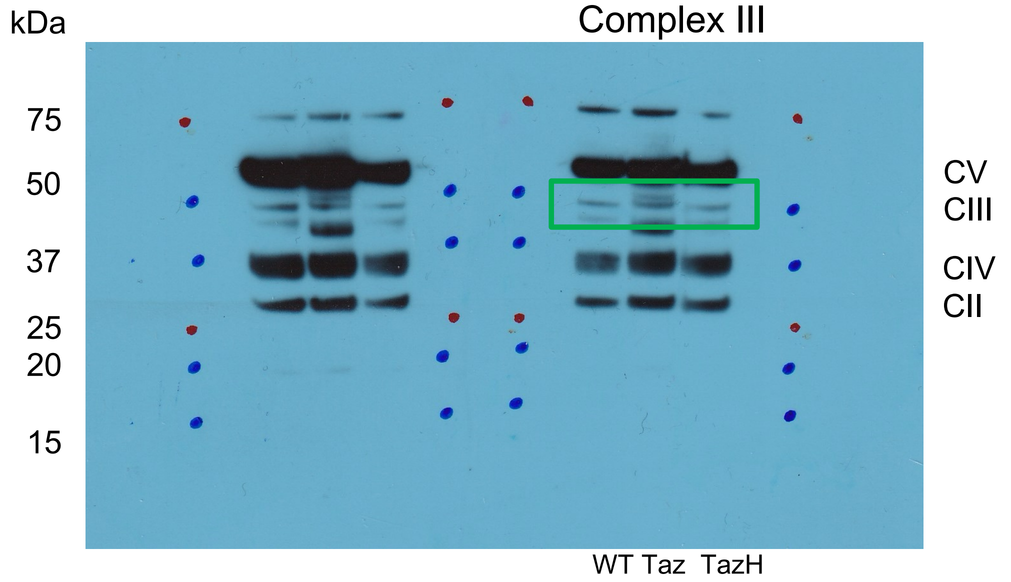

citrate synthase

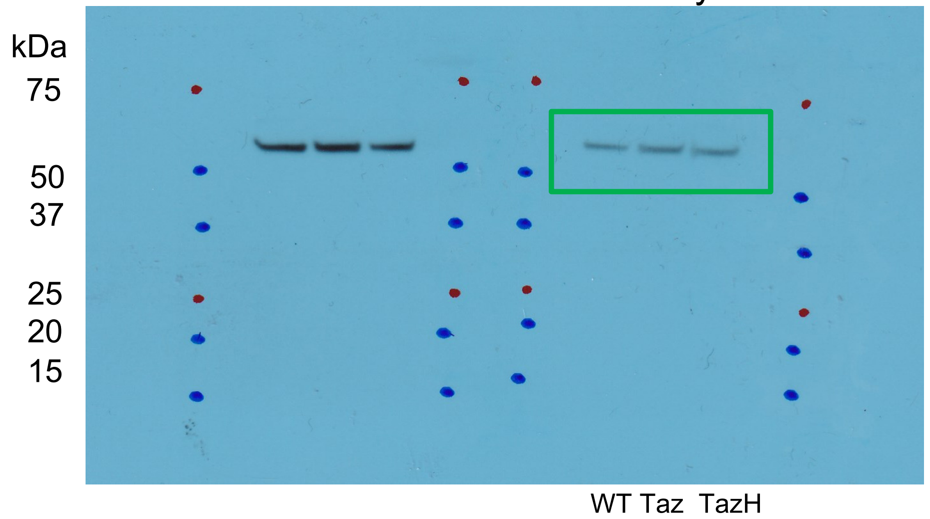

Same membrane as used for Figure 5D (n2)

Full unedited blots for Supplementary Figure 14B

n 3

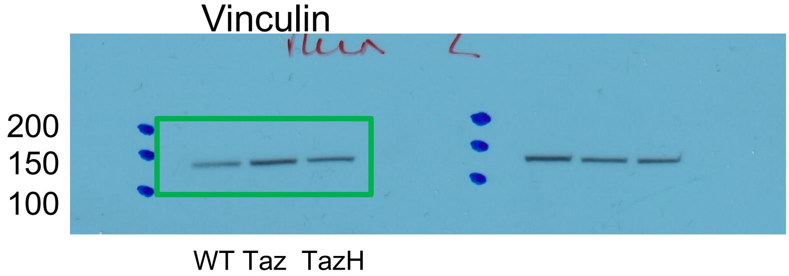

Cut between 75 and 100 kDa

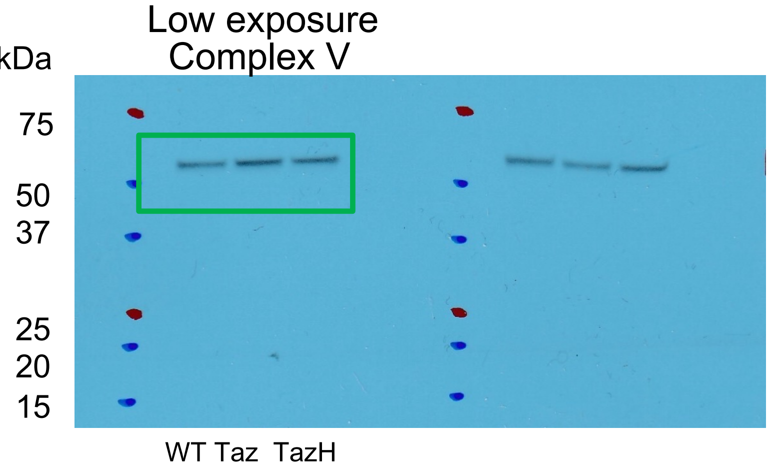

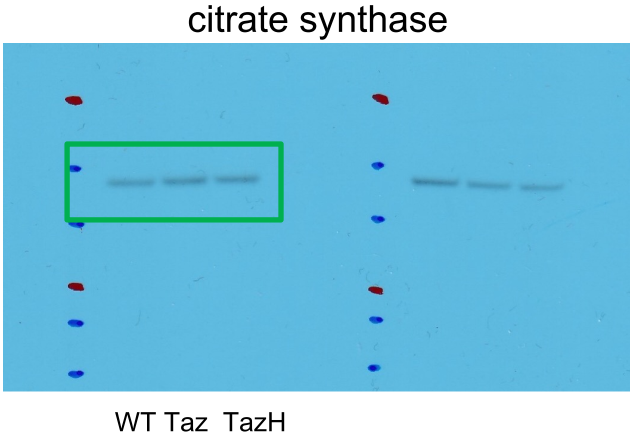

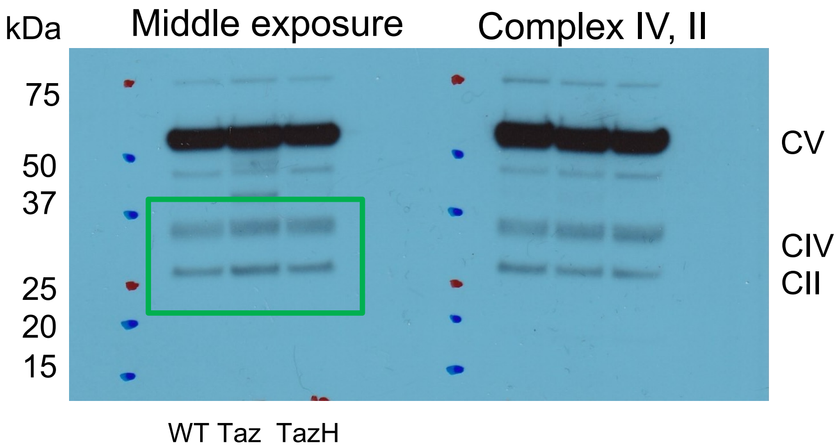

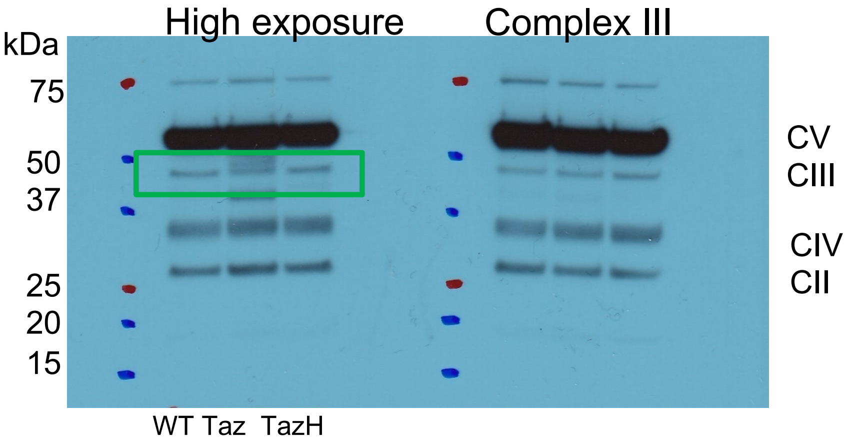

Same membrane as used for Figure 5D (n3)

Full unedited blots for Supplementary Figure 14

n 4

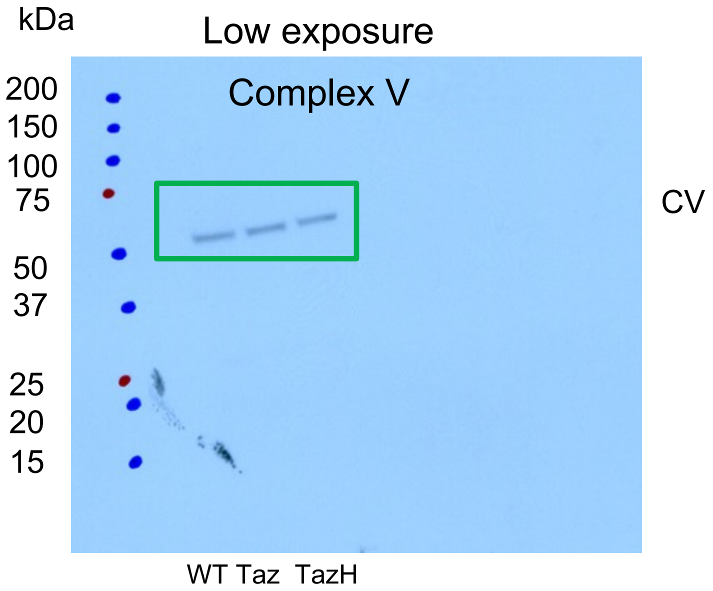

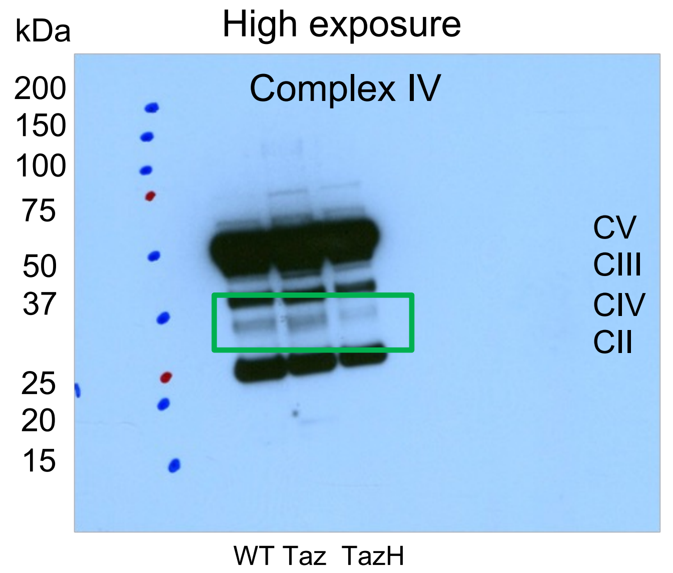

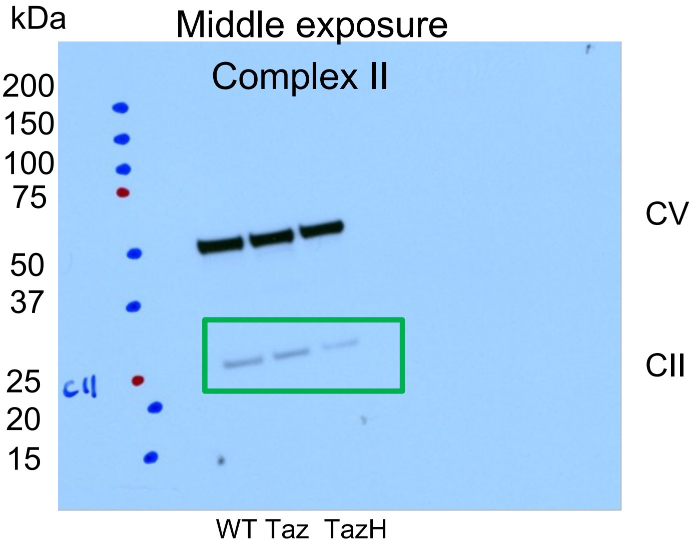

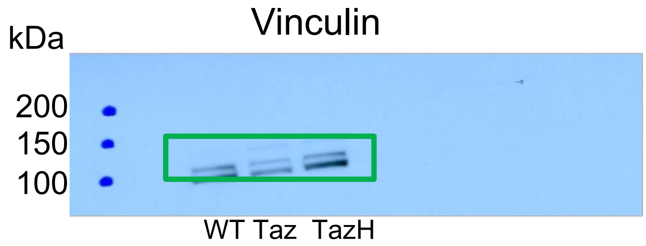

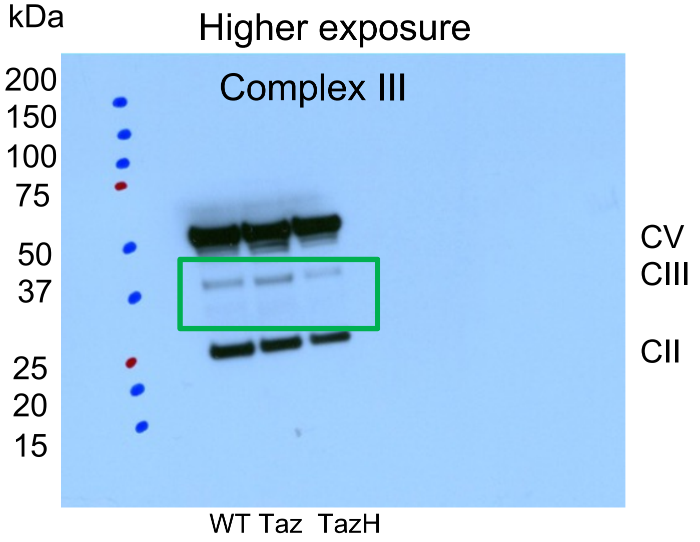

Cut between 75 and 100 kDa

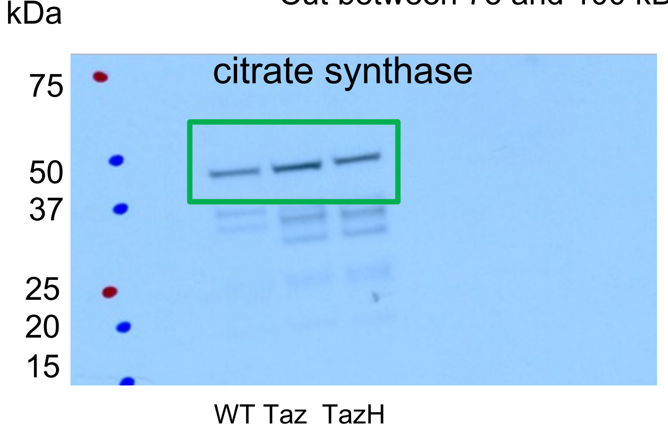

Same membrane as used for Figure 5D (n4)
