# Supplementary material for: Defective vascular smooth muscle cell tafazzin impairs mitochondrial function and promotes atherosclerosis in preclinical models
Source: Nat Commun. 2025 Dec 4;16:10909. doi: 10.1038/s41467-025-65873-y (PMC12678788; doi:10.1038/s41467-025-65873-y)
Supplement: Supplementary file 3 — Reporting Summary [file 41467_2025_65873_MOESM3_ESM.pdf]

nature portfolio

Corresponding author(s): Emma Yu

Last updated by author(s): 06/10/2025

Reporting Summary

Nature Portfolio wishes to improve the reproducibility of the work that we publish. This form provides structure for consistency and transparency in reporting. For further information on Nature Portfolio policies, see our Editorial Policies and the Editorial Policy Checklist.

Statistics

For all statistical analyses, confirm that the following items are present in the figure legend, table legend, main text, or Methods section.

n/a	Confirmed	
☐	☒	The exact sample size (*n*) for each experimental group/condition, given as a discrete number and unit of measurement
☐	☒	A statement on whether measurements were taken from distinct samples or whether the same sample was measured repeatedly
☐	☒	The statistical test(s) used AND whether they are one- or two-sided<br>*Only common tests should be described solely by name; describe more complex techniques in the Methods section.*
☒	☐	A description of all covariates tested
☐	☒	A description of any assumptions or corrections, such as tests of normality and adjustment for multiple comparisons
☐	☒	A full description of the statistical parameters including central tendency (e.g. means) or other basic estimates (e.g. regression coefficient) AND variation (e.g. standard deviation) or associated estimates of uncertainty (e.g. confidence intervals)
☐	☒	For null hypothesis testing, the test statistic (e.g. *F*, *t*, *r*) with confidence intervals, effect sizes, degrees of freedom and *P* value noted<br>*Give P values as exact values whenever suitable.*
☒	☐	For Bayesian analysis, information on the choice of priors and Markov chain Monte Carlo settings
☒	☐	For hierarchical and complex designs, identification of the appropriate level for tests and full reporting of outcomes
☒	☐	Estimates of effect sizes (e.g. Cohen's *d*, Pearson's *r*), indicating how they were calculated

Our web collection on statistics for biologists contains articles on many of the points above.

Software and code

Policy information about availability of computer code

Data collection	Software used for data collection:<br>qPCR: BioRad CFX Maestro 2.2<br>Platereader: Omega software 5.50R4<br>Confocal microscopy: LAS X software 37.2.22383<br>Histology: Image-Pro Insight 9.1<br>Flow cytometry: BD CSampler 1.0.264.21<br>Seahorse: Wave 2.6.1

Data analysis	Software used for data analysis<br>qPCR: BioRad CFX Maestro 2.2<br>Platereader: MARS 3.32 R5W<br>Confocal microscopy: LAS X software 37.2.22383, imageJ macro MitoMAPR<br>Histology: Image-Pro Insight 9.1, Qupath 0.4.4<br>Flow cytometry: BD CSampler 1.0.264.21<br>Seahorse: Wave 2.6.1<br>Western blot densitometry: ImageJ 1.54g<br>scRNAseq: Seurat v.4.0.1 in R v.4.0.4<br>lipidomics: Thermo Scientific Xcalibur (Version 4.1.31.9)<br>GraphPad Prism version 10.2.3

For manuscripts utilizing custom algorithms or software that are central to the research but not yet described in published literature, software must be made available to editors and reviewers. We strongly encourage code deposition in a community repository (e.g. GitHub). See the Nature Portfolio guidelines for submitting code & software for further information.

Data

Policy information about availability of data

All manuscripts must include a data availability statement. This statement should provide the following information, where applicable:

- Accession codes, unique identifiers, or web links for publicly available datasets
- A description of any restrictions on data availability
- For clinical datasets or third party data, please ensure that the statement adheres to our policy

All data supporting the findings of this study are available within the main text, Supplementary Information, and Source data. The mass spectrometry lipidomics data generated in this study have been deposited in the MassIVE database under accession code MSV000099336 [https://doi.org/doi:10.25345/C5RB6WF71]. The ScRNA-seq datasets from coronary and carotid lesions used in this study have been previously published and are available in the NCBI Gene Expression Omnibus database under accession numbers GSE131778 [https://www.ncbi.nlm.nih.gov/geo/query/acc.cgi?acc], GSE155512 [https://www.ncbi.nlm.nih.gov/geo/query/acc.cgi?acc=GSE155512], GSE159677 [https://www.ncbi.nlm.nih.gov/geo/query/acc.cgi?acc=gse159677]. The datasets GSE 131778, GSE155512 and GSE159677 were re-analyzed from integrated scRNA-seq data available at https://zenodo.org/records/1400746114. Source data are provided with this paper.

Research involving human participants, their data, or biological material

Policy information about studies with human participants or human data. See also policy information about sex, gender (identity/presentation), and sexual orientation and race, ethnicity and racism.

Reporting on sex and gender	Age and sex of donors for tissue samples were as as follows:aortas: 67M, 77M, 46M, 75M, 72F, 83F, 78F, 49M, 78M; carotids: 82M, 63M, 66F, 62M, 61M, 72F, 51M, 68M, 81F.<br>Age and sex of donors for cell isolates were as follows: 70F, 37M, 64M, 62M, 54F, 45F, 58M, 68M, 72M, 71M, 62F<br>.<br>Male and female tissue and cell isolates were used in this study with numbers per group indicated in the legends. Male and female data were used for the analyses; post hoc sex based statistical analysis has not been performed due to low sample size.The data disaggregated for sex is included in the source data.<br>Gender information is not available.
Reporting on race, ethnicity, or other socially relevant groupings	We have not reported on race, ethnicity or other socially relevant groupings as this information is not available. Tissue was obtained from anonymised donors.
Population characteristics	Plaque tissue was obtained from patients undergoing carotid endarterectomy and normal aortic tissue from patients undergoing aortic valve surgery or root replacement.
Recruitment	Informed consent was obtained from all participants involved in the study and recruitment performed by Papworth Tissue Bank. No compensation was received by tissue donors.
Ethics oversight	Samples were collected under written informed consent following protocols approved by the Huntingdon Research Ethics Committee and Cambridgeshire 1 Research Ethics Committee.

Note that full information on the approval of the study protocol must also be provided in the manuscript.

Field-specific reporting

Please select the one below that is the best fit for your research. If you are not sure, read the appropriate sections before making your selection.

☒ Life sciences ☐ Behavioural & social sciences ☐ Ecological, evolutionary & environmental sciences

For a reference copy of the document with all sections, see nature.com/documents/nr-reporting-summary-flat.pdf

Life sciences study design

All studies must disclose on these points even when the disclosure is negative.

Sample size	Sample size was determined using power calculations based on observed or expected variation with significance threshold 0.05, power 90%.
Data exclusions	No data were excluded from analyses.
Replication	For the experiments a minimum of 3 independent experiments was performed with consistent results between experiments. VSMC isolates were obtained from different human donors or independent animals for human or mouse VSMCs. The data shown for QPCR, luminescence and seahorse experiments are the averages of technical replicates for each independent sample.
Randomization	For the ApoE-/- and ApoE+/+ experiments mice were randomly allocated to experimental groups. To achieve randomization, SM22a-Taz/ApoE-/- or SM22a-TazH69Q/ApoE-/- were housed in the same cages as their littermate ApoE-/- controls during both breeding and during atherosclerosis study. Experimental groups always consisted of both SM22a-Taz/ApoE-/- or SM22a-TazH69Q/ApoE-/- mice with their littermate controls and these were sacrificed on the same day.
Blinding	investigators were blinded to group allocation during data collection and analysis.

Reporting for specific materials, systems and methods

We require information from authors about some types of materials, experimental systems and methods used in many studies. Here, indicate whether each material, system or method listed is relevant to your study. If you are not sure if a list item applies to your research, read the appropriate section before selecting a response.

Materials & experimental systems

n/a	Involved in the study	
☐	☒	Antibodies
☐	☒	Eukaryotic cell lines
☒	☐	Palaeontology and archaeology
☐	☒	Animals and other organisms
☒	☐	Clinical data
☒	☐	Dual use research of concern
☒	☐	Plants

Methods

n/a	Involved in the study	
☒	☐	ChIP-seq
☐	☒	Flow cytometry
☒	☐	MRI-based neuroimaging

Antibodies

Antibodies used

Antibodies used in the study: Target, Product number, Source, Dilution. Multiple lot numbers were used.
Western blotting
Tafazzin. Ab105104, Abcam. 1 in 1000
Tafazzin sc365810, Santa Cruz. 1 in 1000
Citrate synthase. Ab96600. Abcam. 1 in 1000
total OXPHOS rodent antibody. Ab110413. Abcam, 1:500
Tubulin. 2148. Cell Signaling Technologies. 1:500
Vinculin. Ab219649. Abcam. 1:1000.
phospho NFKB p65. 3031. Cell Signaling, 1:500.
NFKB p65. 8242. Cell Signaling, 1:1000.
MFN1. 14739. Cell Signaling. 1:1000.
MFN2. 11925. Cell Signaling. 1:1000.
Drp1. 611113. BD Transduction Laboratories. 1:1000.
Fis1. 10956-1-AP. Proteintech. 1:1000.
Opa1 612606. BD Transduction Laboratories. 1:1000.
NDUFB8. 147941-1-AP. Proteintech. 1:1000
SDHA. Ab14715. Abcam. 1:1000
UQCRC2. 14742-1-AP. Proteintech. 1:1000
MTCO2. 55070-1-AP. Proteintech. 1:1000
ATP5A1. 660371-1-Ig. Proteintech. 1:1000

Histology: IF
anti-smooth muscle actin. #14395-1-AP. Proteintech. 1 in 100.
tafazzin. sc365810. Santa Cruz Biotechnology.
anti-smooth muscle actin. SMA. 1A4, Dako,1:100
Mac3. 553322. BD Biosciences. 1:100.

Immunohistochemistry
anti-smooth muscle actin. SMA, 1A4, Dako. 1:500.

Mac3. 553322. BD Biosciences. 1:400.
Ki67.Ab 16667. Abcam.1:100.
Alexa FluorTM 488 anti-rabbit antibody.A-21206, Invitrogen, 1 :200
Alexa FluorTM 594 anti-mouse antibody. A-11032, Invitrogen, 1: 200
Alexa FluorTM 488 anti-mouse antibody. A-11001, Invitrogen, 1:200

Immunocytochemistry
Actin, α-smooth muscle-Cy3TM. C6198. Merck,1:200.
smooth muscle myosin heavy chain 11. Ab124679. Abcam. 1:100.
human CD68, M0814. Dako, 1:200.
mouse CD68. ab125212. Abcam. 1;200

Mitochondrial morphology
TOMM20. #311802-1-AP. Proteintech. 1 in 1000.
TOMM70. #14528-1-AP. Proteintech. 1 in 1000
Alexa FluorTM 488 anti-rabbit antibody A-11008, Invitrogen, 1: 1000

Validation

All antibodies used in the study are commercially available, except Ab105104 which was discontinued, and validated by the manufacturer and/or reported in the literature

western
https://www.scbt.com/p/tafazzin-antibody-f-7
https://www.abcam.com/en-us/products/primary-antibodies/citrate-synthetase-antibody-ab96600
https://www.abcam.com/en-us/products/panels/total-oxphos-rodent-wb-antibody-cocktail-ab110413
https://www.cellsignal.com/products/primary-antibodies/a-b-tubulin-antibody/2148
https://www.abcam.com/en-us/products/primary-antibodies/vinculin-antibody-epr20407-ab219649
https://www.cellsignal.com/products/primary-antibodies/phospho-nf-kb-p65-ser536-antibody/3031
https://www.cellsignal.com/products/primary-antibodies/nf-kb-p65-d14e12-xp-rabbit-mab/8242
https://www.cellsignal.com/products/primary-antibodies/mitofusin-1-d6e2s-rabbit-mab/14739
https://www.cellsignal.com/products/primary-antibodies/mitofusin-2-d1e9-rabbit-mab/11925
https://www.bdbiosciences.com/en-us/products/reagents/microscopy-imaging-reagents/immunofluorescence-reagents/purified-mouse-anti-dlp1.611113?tab=product_details
https://www.ptglab.com/products/FIS1-Antibody-10956-1-AP.htm
https://www.bdbiosciences.com/en-gb/products/reagents/microscopy-imaging-reagents/immunofluorescence-reagents/purified-mouse-anti-opa1.612606?tab=product_details
https://www.ptglab.com/products/NDUFB8-Antibody-14794-1-AP.htm
https://www.abcam.com/en-us/products/primary-antibodies/sdha-antibody-2e3gc12fb2ae2-ab14715
https://www.ptglab.com/products/UQCRC2-Antibody-14742-1-AP.htm
https://www.ptglab.com/products/COX2-Antibody-55070-1-AP.htm
https://www.ptglab.com/products/ATP5A1-Antibody-66037-1-Ig.htm

histology IF
https://www.ptglab.com/products/ACTA2-Antibody-14395-1-AP.htm
https://www.agilent.com/en/product/immunohistochemistry/antibodies-controls/primary-antibodies/actin-%28smooth-muscle%29-%28dako-omnis%29-76225
hhttps://www.bdbiosciences.com/en-gb/products/reagents/western-blotting-and-molecular-reagents/purified-rat-anti-mouse-cd107b.553322?tab=product_details

IHC
https://www.abcam.com/en-us/products/primary-antibodies/ki67-antibody-sp6-ab16667

Immunocytochemistry
https://www.sigmaaldrich.com/GB/en/product/sigma/c6198?srsltid=AfmBOooVfo0BiglUV9QdWIMYRiLXsgLrpciDfUyXODEMrCPeFk6jFiYX
https://www.abcam.com/en-us/products/primary-antibodies/smooth-muscle-myosin-heavy-chain-11-antibody-epr5335-ab124679
https://www.agilent.com/store/productDetail.jsp?catalogId=M081401-2
https://www.abcam.com/en-us/products/primary-antibodies/cd68-antibody-ab125212

Mitochondrial morphology
hhttps://www.ptglab.com/products/TOM20-Antibody-11802-1-AP.htm?srsltid=AfmBOopRUtOv8BFv4TXbIsyqeMC8-0dGqvXCvdqun8c2iyVl3TazyXTI
https://www.ptglab.com/products/TOM70-Antibody-14528-1-AP.htm

Eukaryotic cell lines

Policy information about cell lines and Sex and Gender in Research

Cell line source(s)

Primary human and mouse VSMCs were isolated from male and female donors, and cultured by our group as described in the Methods section of the manuscript. HEK293FT cells used for lentiviral generation were sourced from Invitrogen (R7007) and were originally established from a female fetus. THP-1 cells were sourced from American Type Culture Collection (TIB-202) and were originally established from a male donor.

Authentication	Human and mouse VSMCs have been authenticated by immunofluorescence staining for SMA and MYH11, as shown in Supplemental Figures. HEK293FT cells showed characteristic rapid growth rate, high transfection efficiency and generated functional lentivirus particles but were not formally authenticated. THP-1 cells could chracteristically be differentiated into macrophages using PMA and the differentiation was verified by immunofluorescence staining for CD68.
Mycoplasma contamination	Primary VSMCs, HEK293FT cells, and THP-1 cells were regularly tested for mycoplasma and found to be negative.
Commonly misidentified lines (See ICLAC register)	HEK293FT

Animals and other research organisms

Policy information about studies involving animals; ARRIVE guidelines recommended for reporting animal research, and Sex and Gender in Research

Laboratory animals	Mus musculus: All mice were housed in a sterile facility, with 12 hour light/dark cycle, 55+/-10% humidity, at 19-21 C. The Myh11-CreERt273 (strain # 019079, Jackson Laboratory), Rosa26-Confetti74 (strain # 013731, Jackson Laboratory) and ApoE-/- (strain 002052, Jackson Laboratory) mouse lines have been described. Myh11-CreERt2/Rosa26-Confetti/Apoe−/− mice18 used in this study were on a C57BL/6 background and generated through in house breeding. Myh11-CreERt2/Rosa26-Confetti/Apoe−/− were given tamoxifen injections at 6 to 8 weeks old, rested for 1 week, then fed high fat diet for 14-23 weeks. Apoe-/- mice on a C57BL/6 background were sourced from Jackson Laboratory (strain # 002052) and C57BL/6 ApoE wild type mice were purchased from Charles River Laboratories (Apoe+/+, strain # 027, Charles River Laboratories). The mice were fed chow diet until 6 weeks of age and then either sacrificed or fed high fat diet until 20 weeks of age. SM22a-Taz and SM22a-TazH69Q mice were generated by pro-nuclear injection of C57Bl6 embryos with subsequent crossing with ApoE-/- mice (strain 002052, Jackson Laboratory). The mice were fat fed from 6-20 weeks old.
Wild animals	No wild animals were used in this study.
Reporting on sex	Both male and female mice were used in the study The total sample size (male and females combined) per experimental group was determined by power calculation and male and female data used for the analyses. Post hoc sex based statistical analysis has not been performed due to low sample number. The data disaggregated for sex is included in the source data.
Field-collected samples	No field-collected samples were used in this study.
Ethics oversight	All animal experiments were performed under the Animals (Scientific Procedures) Act 1986 Amendment Regulations 2012 (project licence P452C9545) and approved by Cambridge University Animal Welfare and Ethical Review Body (AWERB).

Note that full information on the approval of the study protocol must also be provided in the manuscript.

Plants

Seed stocks	No plant material was used in the study.
Novel plant genotypes	No plant material was used in the study.
Authentication	No plant material was used in the study.

Flow Cytometry

Plots

Confirm that:

- ☐ The axis labels state the marker and fluorochrome used (e.g. CD4-FITC).
- ☐ The axis scales are clearly visible. Include numbers along axes only for bottom left plot of group (a 'group' is an analysis of identical markers).
- ☐ All plots are contour plots with outliers or pseudocolor plots.
- ☐ A numerical value for number of cells or percentage (with statistics) is provided.

Methodology

Sample preparation	Human VSMCs were cultured from aortic or carotid tissue; mouse VSMCs were isolated by enzymatic digestion of mouse aortas. For EdU assays, cells were incubated with 10µmol/L EdU for 24 hours. Cells were then washed, fixed and

permeabilized before incubation with the Click-iT reaction cocktail. For mitoSOX assays cells were incubated in 1µmol/L MitoSOX for 20 minutes at 37ºC. Cells were then washed twice with PBS before flow cytometric analysis. For positive controls cells were incubated with 10µmol/L antimycin A for 20 minutes at 37ºC. For annexin V assays, cells were treated with 30 µmol/L tert-butyl hydroperoxide (t-BHP) for 16 hours to generate positive controls. Cells were then harvested, washed with cold PBS and stained with propidium iodide and Annexin V Alexa Fluor 488 for 15 minutes at room temperature. Samples were then immediately analysed on the flow cytometer.

Instrument

BD Accuri C6 Flow Cytometer (BD Bioscience).

Software

Flow cytometry data was collected and analysed using BD CSampler software.

Cell population abundance

VSMCs that had been authenticated as described above were used for flow cytometry.

Gating strategy

For EdU and mitosox assays a live cell population was gated on FSC/SSC plots. The gate for EdU+ cells was determined based on cells without EdU added. The gate for Mitosox + cells was identifed based on cells that had not been incubated with Mitosox. For annexin V assays, FSC/SSC plots were used to identify all cells but exclude debris. The gate for annexin V/propide positive cells was determined based on unstained cells

☒ Tick this box to confirm that a figure exemplifying the gating strategy is provided in the Supplementary Information.
